# Combined intermittent fasting and ERK inhibition enhance the anti-tumor effects of chemotherapy via the GSK3β-SIRT7 axis

**DOI:** 10.1038/s41467-021-25274-3

**Published:** 2021-08-25

**Authors:** Xiaolong Tang, Guo Li, Lei Shi, Fengting Su, Minxian Qian, Zuojun Liu, Yuan Meng, Shimin Sun, Ji Li, Baohua Liu

**Affiliations:** 1 Shenzhen Key Laboratory of Systemic Aging and Intervention (SKL-SAI), School of Basic Medical Sciences, Shenzhen University, Shenzhen, China; 2 Guangdong Key Laboratory of Genome Stability and Human Disease Prevention, Marshall Laboratory of Biomedical Engineering, National Engineering Research Center for Biotechnology (Shenzhen), International Cancer Center, Shenzhen University, Shenzhen, China; 3 Department of Dermatology, Xiangya Hospital, Central South University, Changsha, China; 4 Shenzhen Bay Laboratory, Shenzhen, China

**Keywords:** Phosphorylation, Breast cancer

## Abstract

Dietary interventions such as intermittent fasting (IF) have emerged as an attractive strategy for cancer therapies; therefore, understanding the underlying molecular mechanisms is pivotal. Here, we find SIRT7 decline markedly attenuates the anti-tumor effect of IF. Mechanistically, AMP-activated protein kinase (AMPK) phosphorylating SIRT7 at T263 triggers further phosphorylation at T255/S259 by glycogen synthase kinase 3β (GSK3β), which stabilizes SIRT7 by decoupling E3 ligase UBR5. SIRT7 hyperphosphorylation achieves anti-tumor activity by disrupting the SKP2-SCF E3 ligase, thus preventing SKP2-mediated K63-linked AKT polyubiquitination and subsequent activation. In contrast, GSK3β-SIRT7 axis is inhibited by EGF/ERK2 signaling, with ERK2 inactivating GSK3β, thus accelerating SIRT7 degradation. Unfavorably, glucose deprivation or chemotherapy hijacks the GSK3β-SIRT7 axis via ERK2, thus activating AKT and ensuring survival. Notably, Trametinib, an FDA-approved MEK inhibitor, enhances the efficacy of combination therapy with doxorubicin and IF. Overall, we have revealed the GSK3β-SIRT7 axis that must be fine-tuned in the face of the energetic and oncogenic stresses in malignancy.

## Abstract (web summary)

The combination of intermittent fasting and chemotherapy can improve the response to treatment. Here, the authors show that SIRT7 activation is required to inactivate Akt during intermittent fasting and that the combination of intermittent fasting and inhibitors that block the Erk pathway can improve efficacy of treatment.

## Introduction

Solid tumor cells are inevitably exposed to energy stress during disease progression as a result of rapid proliferation and abnormal intratumoral vasculature^1^. For instance, poorly vascularized areas of tumors are almost completely deprived of glucose compared to normal tissues^2^. To survive, cancer cells have evolved multiple mechanisms to protect against stress-induced damage. AMP-activated protein kinase (AMPK) and AKT are among the modulators of the effects of metabolic stress^3^. Glucose deprivation (GD) activates AMPK to promote catabolic metabolism for energy production^4^. In contrast, AKT enhances anabolic metabolism for energy restoration in tumor cells^5^. AKT is overactivated in many cancer types and dictates cancer cell survival, metastasis, and chemoresistance^6^. Intriguingly, fasting has emerged as an attractive anti-cancer strategy through mechanisms involving the AMPK/AKT/GSK3β axis^7^. Recently, the anti-neoplastic effect of fasting was found to rely on glycogen synthase kinase 3β (GSK3β)^8^; fasting combined with hormone therapy effectively promotes long-lasting tumor regression and protects against drug resistance by suppressing AKT activation^9^. However, the intrinsic coordination between energetic and oncogenic stresses in tumor initiation and malignant progression remains unclear.

EGF/EGFR signaling is hyperactive in a broad array of human cancers including malignant breast cancer, skin tumor, non-small cell lung cancer (NSCLC), colorectal cancer, and pancreatic cancer^10^. Hyperactivated EGF/EGFR stimulates intracellular pathways, such as RAS/ERK and PI3K/AKT, which inactivate GSK3β by Ser9 phosphorylation^11^. As a serine/threonine kinase, GSK3β has a strong preference for substrates harboring a ‒S/TXXXS/T‒ motif (X, any amino acid), which is usually primed as a target for GSK3β by phosphorylation of a residue four or five amino acids to the C-terminal side of the motif^12^. The Ser9 phosphorylation competes for the phosphate-binding site of primed substrates, thus blocking GSK3β kinase activity^13^. GSK3β-mediated phosphorylation can render substrates as targets for proteasomal destruction, thus inactivating various oncogenic proteins, such as β-catenin and c-Myc, to suppress tumor development^11^.

SIRT7 is widely expressed in various organs/tissues and was initially reported to be localized in the nucleolus, thus regulating protein synthesis and cell proliferation^14^. Despite being the least well-characterized member of the sirtuin family of proteins, accumulating evidence suggests that SIRT7 acts as a stress regulator in physiological homeostasis^15–17^. *Sirt7*^−/−^ mice suffer genomic instability, cardiomyopathy, hepatic lipid metabolism dysregulation, osteopenia, and lifespan reduction^18–22^. Inactivation of SIRT7 compromises the regenerative capacity of hematopoietic stem cells by increasing mitochondrial protein-folding stress^23^. Under conditions of energy deprivation, SIRT7 is released from the nucleolus, resulting in repression of ribosomal DNA (rDNA) transcription^17,24^. In addition, SIRT7 promotes the DNA damage response and DNA repair through diverse mechanisms^18,25,26^. In this study, we identified SIRT7 as a substrate of GSK3β. SIRT7 underpins the anti-tumor effect of intermittent fasting (IF) by enhancing the effects of fasting on GSK3β activity and AMPK signaling. SIRT7 fine-tunes the balance between energy stress and oncogenic signals, thus providing a potential therapeutic strategy against multiple cancers and age-related diseases.

## Results

### SIRT7 underpins the anti-neoplastic effect of fasting

**Fig. 1:**
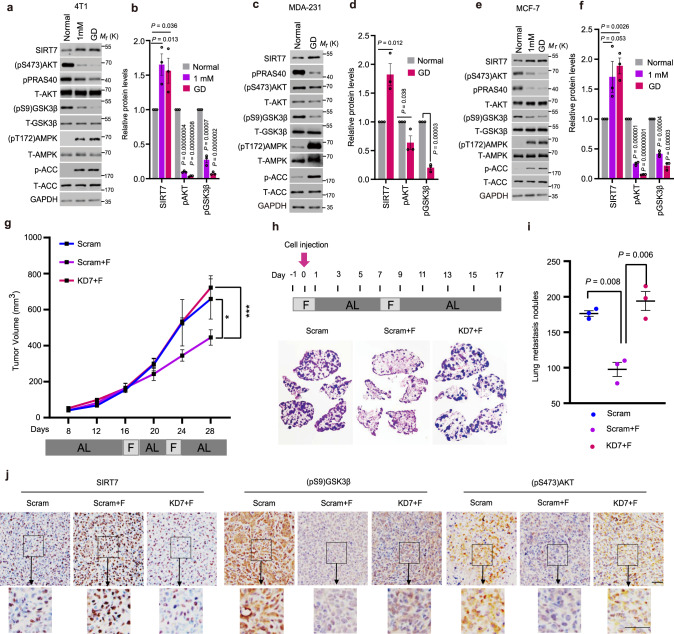
Intermittent fasting inhibits tumor growth and metastasis via SIRT7. **a**–**f** Immunoblotting and quantification of protein levels in 4T1 murine mammary cancer cells (**a**, **b**), MDA-231 (**c**, **d**), and MCF-7 human breast cancer cells (**e**, **f**) cultured with the indicated doses of glucose for short-term treatment. The relevant quantifications were collected from three independent experiments. **g** 4T1 cells stably transfected with Scramble (Scram) and *Sirt7* knockdown (KD7) shRNAs were injected into the mammary fat pad of female Balb/c mice. Mice in the Scram + F and KD7 + F groups were subjected to intermittent fasting (IF) for 48 h from day 16 and day 22, respectively; otherwise, all animals were fed ad libitum (*n* = 5 mice in the scramble group and *n* = 6 in each of the fasting groups). Tumor size was measured using Vernier calipers. **h**, **i** Scram and KD7-transfected 4T1 cells were injected into the tail vein of female Balb/c mice on day 0 (arrow) (*n* = 3 mice in each group). Mice were subjected to IF as indicated; otherwise, animals were fed ad libitum. Lung metastatic tumor nodules were analyzed by H&E staining (**h**) and the number of nodules was counted in the whole lung (**i**). **j** Immunohistochemistry (IHC) staining showing the levels of SIRT7, (pS473)AKT, and (pS9)GSK3β in tumor samples of Fig. 1g. Scale bar, 50 µm. Data represent means ± SEM (**b**, **d**, **f**, **g**, **i**). *P* values were calculated by two-tailed Student’s *t*-test (**b**, **d**, **f**, **i**) or two-way ANOVA analysis (**g**), *P = 0.03, ***P = 0.0000002. Representative results were obtained from at least three independent experiments. Source data are provided as a Source Data file.

Fasting elicits hypoglycemia to prevent tumor progress^7^. SIRT7 acts as a metabolic regulator^15^. To determine the effect of fasting on SIRT7, we cultured cancer cell lines under conditions of normal glucose concentration (mouse mammary tumor 4T1 cells, approximately 10 mM; human breast cancer MDA-231 and MCF-7 cells, approximately 5 mM), low-glucose concentration (1 mM) or GD to mimic the normal diet, hypoglycemia, or acute energy stress as previously reported^27,28^. Interestingly, increased SIRT7 levels were found in 4T1 cells, MDA-231, and MCF-7 cells cultured under short-term treatments of low or GD glucose conditions (Fig. 1a–f). The elevated SIRT7 levels were accompanied by increased (pT172) AMPKα levels and decreased (pS9)GSK3β and (pS473)AKT levels, representing activated AMPKα, inactivated GSK3β and inhibited AKT, respectively. A previous study showed that a ketogenic diet applied to reduce blood glucose levels combined with PI3K inhibitors has great potential for preventing insulin feedback, which in turn activates AKT for breast cancer therapy^29^. Similar results were observed in BT-549 and MDA-MB-468 breast cancer cells, both of which were used in the ketogenic diet study (Supplementary Fig. 1a, b), suggesting the widespread existence of common mechanism underlying the response to glucose insufficiency. Moreover, these findings are consistent with previous reports showing that fasting activates AMPK and GSK3β, but suppresses AKT activity^8,9^.

We next investigated the role of SIRT7 in fasting-induced tumor growth retardation. To that end, we generated tumor xenografts by implanting 4T1 cells that were stably transfected with *Sirt7* shRNA (KD7) or scramble shRNA (Scram) in female Balb/c mice subjected to IF for 48 h starting on day 16 and day 22, respectively; otherwise, all animals were provided with food ad libitum. Strikingly, the growth retardation effect of IF on 4T1 cell-derived tumors was markedly inhibited by KD7-mediated silencing of *SIRT7* expression (Fig. 1g and Supplementary Fig. 1c).

Following entry into the blood circulation, cancer cells directly sense the energy status. Thus, we investigated the role of SIRT7 in the mechanism by which fasting suppresses cancer lung metastasis. To this end, mice were divided into three groups: mice fed ad libitum and injected with Scram 4T1 via the tail vein (Scram), mice under IF injected with Scram 4T1 (Scram + F), and mice under IF injected with KD7 4T1 (KD7 + F) (Fig. 1h). At 17 days after cell injection, the mice were sacrificed and lung metastatic nodules were analyzed. Although IF resulted in a significant decrease in the number of lung metastatic nodules (Scram vs. Scram + F), KD7 significantly attenuated its efficacy (Scram + F vs. KD7 + F) (Fig. 1h, i). IHC analysis of tissue sections prepared from the samples used in Fig. 1g revealed that IF increased SIRT7 levels, while the levels of (pS9)GSK3β and (pS473)AKT were decreased (Fig. 1j). Interestingly, KD7 abolished the inhibition of AKT by IF, while GSK3β activation was merely reduced, indicating that under fasting conditions, GSK3β and AKT function upstream and downstream of SIRT7, respectively. Of note, the reduction in body weight observed during fasting was largely recovered after the re-introduction of food (Supplementary Fig. 1d, e). These data suggest that SIRT7 contributes to the anti-neoplastic effect of fasting.

### AMPK primes SIRT7 for phosphorylation by GSK3β under conditions of GD

**Fig. 2:**
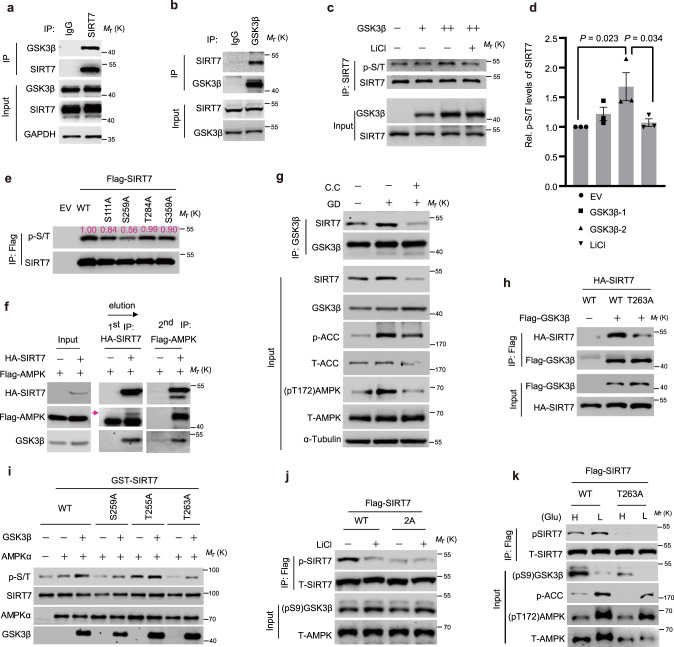
AMPK primes SIRT7 phosphorylation by GSK3β. **a**, **b** Immunoblots showing the interaction of endogenous SIRT7 and GSK3β by immunoprecipitation of the indicated proteins. **c**, **d** Immunoblotting and related quantification (*n* = 3 biologically independent samples) of SIRT7 pan p-S/T levels in HEK293 cells transfected with increasing amounts of GSK3β plasmids with or without LiCl (10 mM) treatment; error bars: means ± SEM; *P* values were calculated by one-tailed Student’s *t*-test. **e** SIRT7 and the indicated mutants were overexpressed in HEK293 cells and then extracted for immunoblotting analysis of phosphorylation levels with an anti-pan-p-S/T antibody. The relative intensity measured by Image J® and is shown the individual bands. **f** Immunoblotting analysis of the sequential immunoprecipitations based on anti-HA or anti-FLAG antibodies in HEK293 cells co-expressing ectopic HA-SIRT7 and Flag-AMPK. Arrow indicates the specific band for FLAG-AMPK. **g** Immunoblots showing the binding of endogenous SIRT7 to GSK3β in HEK293 cells under glucose starvation (1 h) or Compound C treatment (CC, 5 µM). **h** Immunoblots showing the binding of WT-SIRT7 or T263A to GSK3β in HEK293 cells transfected with the indicated plasmids. **i** Immunoblotting analysis of SIRT7 phosphorylation levels after in vitro kinase assays based on active GSK3β and AMPK complexes immunoprecipitated from AICAR-treated HEK293 cells. SIRT7 phosphorylation was detected by probing with an anti-pan p-S/T antibody. **j**, **k** Immunoblots showing SIRT7 phosphorylation levels in HEK293 cells treated as indicated. The anti-p-SIRT7 antibody was generated by immunization with a Thr255/Ser259 phosphorylated peptide. Representative results were obtained from at least three independent experiments. Source data are provided as a Source Data file.

The anti-neoplastic effect of fasting relies, at least partially, on GSK3β and AMPK^8,30^. Our observation of the concomitant activation of GSK3β and AMPK and SIRT7 upregulation following IF indicates the existence of potential links between SIRT7, GSK3β and AMPK. Interestingly, an interaction between endogenous SIRT7 and GSK3β was observed, in that GSK3β and SIRT7 were immunoprecipitated by anti-SIRT7 and anti-GSK3β, respectively, but not by the mouse/rabbit IgG control (Fig. 2a, b). This was verified by the interaction between HA-SIRT7 and Flag-GSK3β overexpressed in HEK293 cells (Supplementary Fig. 2a, b). GST-pulldown assays with recombinant GST-SIRT7 and His-GSK3β proteins confirmed a direct interaction between SIRT7 and GSK3β (Supplementary Fig. 2c). The truncation analysis revealed that the catalytic domains (CA) of SIRT7 and GSK3β mediated the interaction between the two proteins (Supplementary Fig. 2d, e).

GSK3β kinase recognizes a –S/TXXXpS/T– motif in its substrates^31^, wherein pS/T represents a priming phosphorylation catalyzed by an independent kinase^12^. Indeed, multiple, conserved GSK3β phosphorylation sites (S111, S259, T284, and S359) were predicted in the SIRT7 peptide (Supplementary Fig. 3a). Further assessment using anti-pan p-S/T antibodies showed that GSK3β overexpression enhanced SIRT7 phosphorylation in a dose-dependent manner, whereas this effect was apparently attenuated by inhibition of GSK3β kinase activity through lithium chloride (LiCl) treatment^32^ (Fig. 2c, d). To gain more experimental support, we mutated SIRT7 relevant S/T residues in the predicted motifs to A (i.e., S111A, S259A, T284A, and S359A), and evaluated phosphorylation levels. Interestingly, S259A failed to retain phosphorylation (Fig. 2e), suggesting that SIRT7 S259 is a site of phosphorylation mediated by GSK3β.

GSK3β activity usually requires a priming phosphorylation^12^. Interestingly, adjacent to S259, a C-terminal T263 residue was predicted to be a phosphorylation target of AMPK (Supplementary Fig. 3b, c). Indeed, AMPKα1 was also detected in the anti-SIRT7 immunoprecipitates together with GSK3β (Supplementary Fig. 2f). The sequential immunoprecipitation assay confirmed a complex consisting of AMPKα, GSK3β and SIRT7 (Fig. 2f). Treatment with the AMPKα inhibitor Compound C (CC)^30^, inhibited the binding of GSK3β and SIRT7 under conditions of GD that activate AMPK (Fig. 2g). Moreover, the T263A mutation greatly compromised the binding between SIRT7 and GSK3β (Fig. 2h), highlighting the importance of priming phosphorylation. Thus, we postulated that T255/S259 might be sequentially phosphorylated by GSK3β, with T263 as a priming phosphorylation site. To confirm this, we conducted in vitro kinase assays using recombinant active GSK3β and the AMPK complexes purified from HEK293 cells pre-treated with the AMPK activator AICAR^33^. GSK3β obviously increased SIRT7 phosphorylation levels in the presence of AMPK complex, whereas this effect was largely attenuated by T263A and S259A (Fig. 2i). We then generated an antibody against SIRT7 phosphorylated at T255/S259 (p-SIRT7) and confirmed its specificity using dephosphorylation assays with λ-PPase (Supplementary Fig. 3d). In accordance with this, p-SIRT7 was detected in the WT but not in SIRT7 T263A and 2A (T255A/S259A double mutation) mutants (Supplementary Fig. 3e). While p-SIRT7 levels were reduced by LiCl-mediated GSK3β inactivation and increased by AMPK activation under low-glucose conditions, the phosphorylation levels of SIRT7-2A and T263A mutants were not affected by these treatments (Fig. 2j, k). These data suggest that under conditions of fasting/GD, AMPK phosphorylates SIRT7 at T263 to prime GSK3β phosphorylation at T255/S259.

### GSK3β phosphorylation stabilizes SIRT7 by decoupling UBR5

**Fig. 3:**
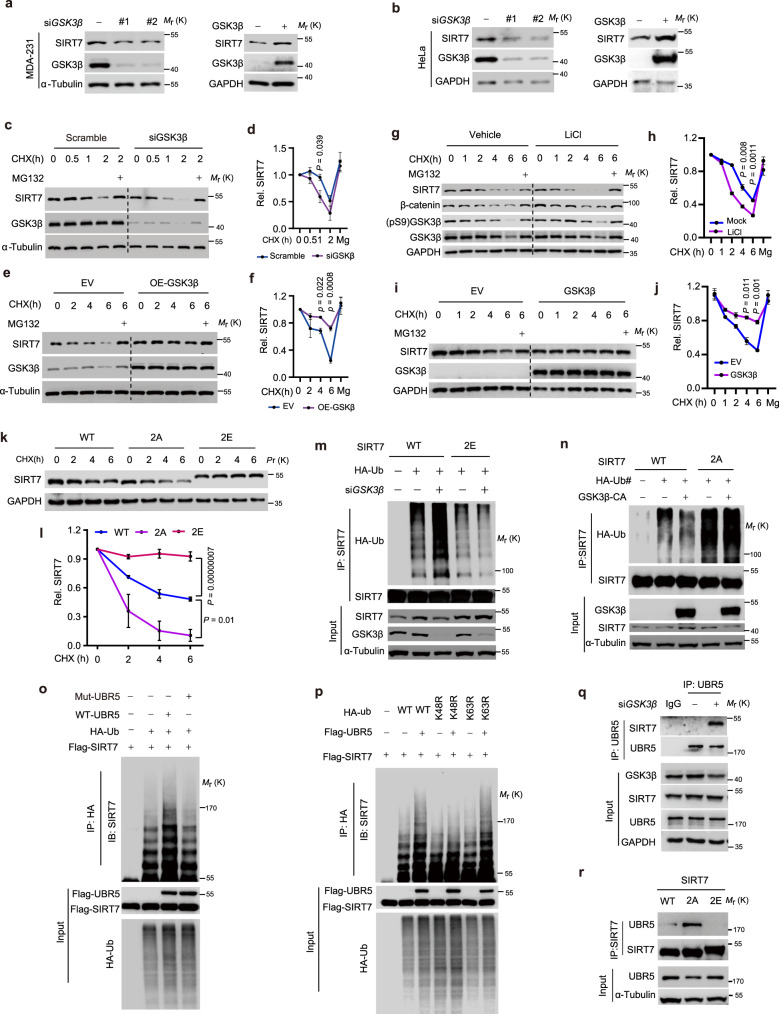
GSK3β stabilizes SIRT7 by phosphorylation-dependent decoupling of UBR5. **a**, **b** Immunoblots showing SIRT7 levels in MDA-231 (**a**) and HeLa cells (**b**) following *GSK3β* knockdown or overexpression. Scr, scrambled siRNA; #1 and #2, two independent siRNAs targeting *GSK3β* mRNA. **c**–**j** Immunoblots showing SIRT7 levels in MDA-231 (**c**–**f**) and HeLa cells (**g**–**j**) following the indicated treatments (**c**, **e**, **g**, **i**); the related quantifications (**d**, **f**, **h**, **j**) were based on three independent experiments; LiCl, 10 mM; MG132, 10 µM. **k**, **l** Immunoblots showing SIRT7 levels in CHX (50 µg/ml) chase assay of HEK293 cells transfected with SIRT7-WT/-2A/-2E (**k**) and the related quantification (**l**) was based on three independent experiments. **m**–**p** Immunoblots showing SIRT7 ubiquitination levels in HEK293 cells following transfection with the indicated plasmids/siRNAs. K63R or K48R represented the corresponding lysine was replaced by arginine on ubiquitin, respectively. **q** Binding of endogenous SIRT7 to UBR5 was assessed in cells with or without *GSK3β* knockdown. **r** Binding of SIRT7-WT and the indicated mutants with UBR5 was evaluated in HEK293 cells. Data represent means ± SEM (**d**, **f**, **h**, **j**, **l**). *P* values were determined by two-way ANOVA analysis. Representative results were observed from at least three independent experiments. Source data are provided as a Source Data file.

GSK3β-mediated phosphorylation is widely reported to modulate substrate protein stability^11^. Initially, we noticed that *GSK3β* knockdown (KD) or inhibition of kinase activity by LiCl downregulated SIRT7 protein levels (Supplementary Fig. 4a). Similar results were obtained in various cell lines, e.g., MDA-231 and BT-549 breast cancer cells, 4T1 murine mammary cancer cells (Fig. 3a, left panel and Supplementary Fig. 4c–e) and HeLa cells (Fig. 3b, left panel). In contrast, GSK3β overexpression increased SIRT7 levels (Fig. 3a, b, right panels). Given that *SIRT7* mRNA levels were barely changed following *GSK3β* KD or LiCl treatment (Supplementary Fig. 4b), we postulated that GSK3β enhances SIRT7 protein stability. Indeed, cycloheximide (CHX) chase assays showed that *GSK3β* KD significantly enhanced SIRT7 protein turnover in MDA-231 and HeLa cells (Fig. 3c, d and Supplementary Fig. 4f). To further investigate whether this effect was dependent on GSK3β kinase activity, LiCl-treated cells were subjected to CHX chase assays, which confirmed that GSK3β inactivation promoted SIRT7 degradation (Fig. 3g, h). Of note, the proteasome inhibitor MG132 largely restored SIRT7 expression in the *GSK3β* KD and LiCl-treated cells, indicating proteasome-related degradation. Conversely, SIRT7 was stabilized in these cells by overexpression of ectopic GSK3β (Fig. 3e, f, i, j). Importantly, silencing of *GSK3α*, the other isoform of GSK3, had little effect on SIRT7 protein level or stability (Supplementary Fig. 4g, h), suggesting that GSK3β plays a unique regulatory role in SIRT7 protein stability.

We next examined whether GSK3β-mediated T255/S259 phosphorylation is required for SIRT7 protein stabilization. CHX chase assays showed that the SIRT7 T255E/S259E mutant (2E), which mimics the hyperphosphorylated state, seemed to be more stable than the WT, whereas SIRT7-2A, which mimics the hypophosphorylated state, underwent more rapid turnover than the WT (Fig. 3k, l). Similarly, the turnover of SIRT7 T263A protein was more rapid than that of the WT, whereas T263D, which mimics the constitutively primed phosphorylation state, was more stable (Supplementary Fig. 4i, j). Furthermore, compared with the SIRT7-WT, SIRT7-2E displayed lower ubiquitylation levels, while the ubiquitylation of SIRT7-2A was more prominent (Fig. 3m, n), demonstrating that 2E was not affected by GSK3β silencing and 2A did not respond to GSK3β overexpression. These data confirmed T255/S259 as unique sites that are phosphorylated by GSK3β, thereby contributing to SIRT7 protein stabilization.

SIRT7 possesses NAD^+^-dependent deacetylase activity^34^. Accumulating evidence has demonstrated the expression of SIRT7 in multiple cellular locations, from the cytoplasm to the nucleus^16,17,35^. We then investigated the effect of phosphorylation by GSK3β on the intrinsic characteristics of SIRT7 protein. We generated MDA-231 stable cell lines overexpressing SIRT7-WT, 2A, and 2E. SIRT7-2A cells showed the highest SIRT7 mRNA expression, but the lowest protein level. Although SIRT7-2E cells had SIRT7 mRNA levels equivalent to those in the SIRT7-WT, the protein levels were much higher. These observations confirmed the existence of intrinsic differences in protein stability among the SIRT7 mutants (Supplementary Fig. 4k). Interestingly, GSK3β inactivation preferentially destabilized the cytoplasmic SIRT7, whereas the nuclear fraction was less prominently affected and even showed comparable levels of stability to the cells without GSK3β inactivation (Supplementary Fig. 4l). SIRT7-WT, 2A and 2E mutants indiscriminately deacetylated H3K18 (Supplementary Fig. 4m), a well-characterized substrate of SIRT7 (ref. ^36^), suggesting a less significant effect of the nuclear location and deacetylase activity. Additionally, SIRT7 mutants shared equivalent ability to deacetylate FKBP51 (Supplementary Fig. 6f). Taken together, these results indicate that SIRT7 phosphorylation by GSK3β dictates the fate of cytoplasmic SIRT7, with less impact on its deacetylase activity. In support of this, we found that aspects of cytoplasmic endogenous SIRT7, including protein expression, stability, and phosphorylation, were much more sensitive to GSK3β inactivation than the nuclear fraction (Supplementary Fig. 4n, o). The much higher abundance of cytoplasmic GSK3β per se (Supplementary Fig. 4n) lends weight to these results.

We then searched the published mass spectrometry database for potential SIRT7 interacting proteins, and noticed an E3 ligase UBR5 that is reported to modulate cellular response to glucose^37,38^. Indeed, an interaction between SIRT7 and UBR5, as well as increased SIRT7 ubiquitination following UBR5 overexpression was observed (Supplementary Fig. 5a–c). Loss of UBR5 restored SIRT7 protein levels in cells treated with LiCl or *GSK3β* siRNA (Supplementary Fig. 5d, e). In contrast, the E3 ligase β-TrCP1 that usually degrades GSK3β substrates had only a marginal effect on SIRT7 and again, *SIRT7* mRNA levels were barely changed (Supplementary Fig. 5d–f). In addition, *UBR5* KD prevented the SIRT7 degradation induced by GSK3β inactivation or KD (Supplementary Fig. 5g–i). Furthermore, the stability or expression of GSK3β protein was not significantly changed by UBR5 KD or overexpression, confirming the direct regulation of SIRT7 by UBR5 (Supplementary Fig. 5j, k). We then explored whether the mechanism by which UBR5 induces SIRT7 instability is dependent on E3 ligase activity. To that end, an E3 ligase activity-deficient mutant of UBR5 was generated by mutating the critical residue cysteine 2768 to alanine (UBR5C2768A, Mut-UBR5), as previously reported^39^. WT-UBR5 clearly facilitated SIRT7 polyubiquitination, while the mutant failed to do so (Fig. 3o). Moreover, the K63-linkage of ubiquitination was marginally affected, indicating that UBR5 mediates the K48-linked polyubiquitination of SIRT7 (Fig. 3p). This finding is consistent with a previous report that K63-modified ubiquitination does not contribute to SIRT7 instability^40^.

We further examined the mechanism by which GSK3β phosphorylation stabilizes SIRT7. Interestingly, *GSK3β* KD or LiCl-mediated inactivation of kinase activity increased the binding of SIRT7 with UBR5 (Fig. 3q and Supplementary Fig. 5l). Conversely, GSK3β-CA (constitutively activated, GSK3β-S9A) markedly suppressed the interaction of UBR5 and SIRT7, whereas the GSK3β-KD (kinase dead, GSK3β-K85M/K86I) or GSK3β-R96A (recognizes only non-primed substrates) had little effect (Supplementary Fig. 5m). Moreover, SIRT7-2A showed a profoundly enhanced interaction with UBR5, whereas the interaction was almost abolished by the 2E mutation (Fig. 3r). Overall, our data indicate that GSK3β-mediated phosphorylation decouples SIRT7 from UBR5 leading to protein stability.

### Boosting the GSK3β–SIRT7 axis mimics the anti-tumor effect of fasting

**Fig. 4:**
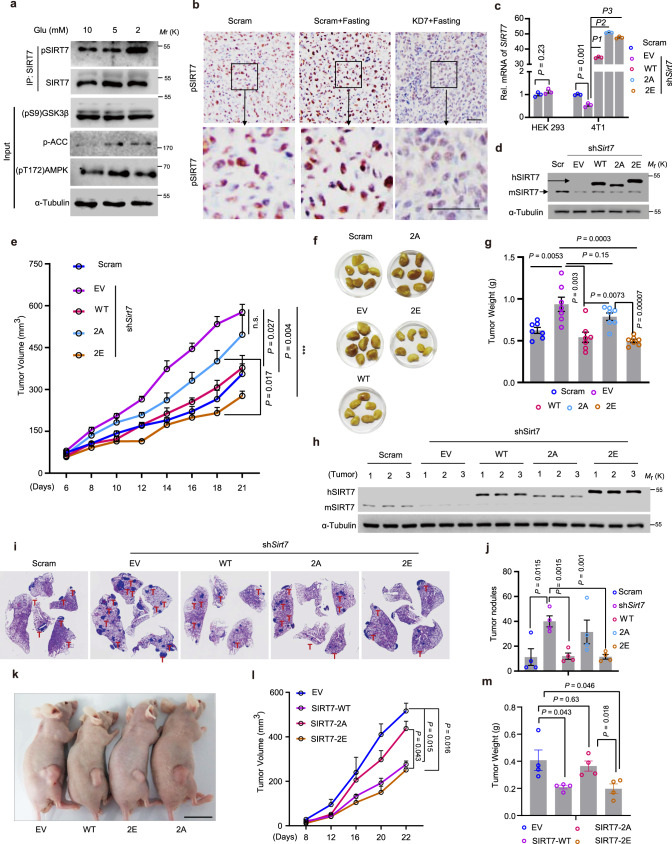
The GSK3β–SIRT7 axis underlies the anti-tumor effects of fasting. **a** Immunoblotting analysis of SIRT7 phosphorylation (p-SIRT7) levels in 4T1 murine mammary tumor cells cultured with different concentrations of glucose (Glu). **b** Representative images of IHC staining showing p-SIRT7 levels in isolated tumor samples (related to Fig. 1g, *n* = 3 mice for analysis per group); scale bar, 50 µm. **c**, **d** Quantitative PCR (**c**, *n* = 3 biologically independent samples) and immunoblots (**d**) showing the expression of hSIRT7 WT and indicated mutants in 4T1 cells in which endogenous m*Sirt7* was knocked down by sh*Sirt7*. HEK293 cells were transfected with sh*Sirt7* to confirm the species specificity of shRNA. EV empty vector, WT human SIRT7-WT, 2A human SIRT7-2A, 2E human SIRT7-2E. **e** 4T1 cells prepared in (**c**) were injected into the mammary fat pad of female Balb/c mice (*n* = 7 per group). Tumor size was measured using Vernier calipers. **f**, **g** Images showing tumor xenografts isolated from (**e**) at the end of experiment and tumor weight (**g**). **h** Immunoblotting analysis of SIRT7 expression in tumor samples extracted from (**e**). **i**, **j** Representative images showing lung metastatic tumor nodules in mice (*n* = 4 mice per group) injected with 4T1 cells as described in (**c**). Lung metastasized tumor nodules counted from whole-lung sections after H&E staining are shown in (**j**). **k**–**m** Representative images (**k**), tumor growth rate (**l**), and average tumor weight (**m**) of H1975 lung cancer cells expressing EV, SIRT7-WT, SIRT7-2A, or SIRT7-2E following inoculation of nude mice with cancer cells (*n* = 4 mice per group). Data represent means ± SEM (**c**, **e**, **g**, **j**, **l**, **m**). *P* values were determined by two-tailed Student’s *t*-test (**c**, **g**, **j**, **m**) or two-way ANOVA analysis (**e**, **l**), ***P = 0.00003, *P1* = 0.00000004, *P2* = 0.0000000024, *P3* = 0.000000031. Representative results were analyzed from at least three independent experiments. Source data are provided as a Source Data file.

Given that fasting activates GSK3β and AMPK, and *SIRT7* silencing significantly attenuated the anti-tumor effect of fasting (Fig. 1), we next examined the potential for manipulation of the GSK3β–SIRT7 axis to mimic the fasting effect. We found that GD increased pSIRT7 levels in 4T1 cells (Fig. 4a); fasting had a similar effect on SIRT7 in the established tumors shown in Fig. 1g (Fig. 4b). Moreover, *Gsk3β* KD reduced SIRT7 expression in 4T1 cells (Supplementary Fig. 4e). These results indicate that 4T1 cells respond to GD via the GSK3β–SIRT7 signaling axis and are suitable for recapitulation of the relevant physiological functions of SIRT7 phosphorylation. We next overexpressed human SIRT7-WT, SIRT7-2A, or SIRT-2E in 4T1 cells stably expressing KD7 (sh*Sirt7*) (Fig. 4c, d) and observed their effects on tumor progression. *Sirt7* KD significantly promoted tumor growth and while SIRT7-WT and SIRT7-2E apparently counteracted this effect, SIRT7-2A did not (Fig. 4e), indicating that the hyperphosphorylated form, SIRT7-2E, mediated the most effective inhibition. This observation was further confirmed by comparing the burden of tumors at the end of experiment (Fig. 4f, g). Importantly, analysis of the tumor xenografts showed that endogenous SIRT7 (mSIRT7) expression remained low, suggesting that this effect is determined by ectopic expression of relevant SIRT7 mutants (Fig. 4h). Moreover, *Sirt7* KD significantly accelerated lung metastasis and while SIRT7-WT and 2E antagonized lung colonization, SIRT7-2A did not (Fig. 4i, j). In another xenograft tumor model established using lung cancer H1975 cells in nude mice, SIRT7-WT and SIRT7-2E overexpression consistently retarded tumor growth (Fig. 4k, l) and significantly relieved tumor burden compared to the control (EV) and SIRT7-2A (Fig. 4m). Thus, our findings indicate that targeting the GSK3β–SIRT7 axis might recapitulate the effects of fasting on tumor growth and metastasis.

### The GSK3β–SIRT7 axis antagonizes EGF-driven AKT activation

**Fig. 5:**
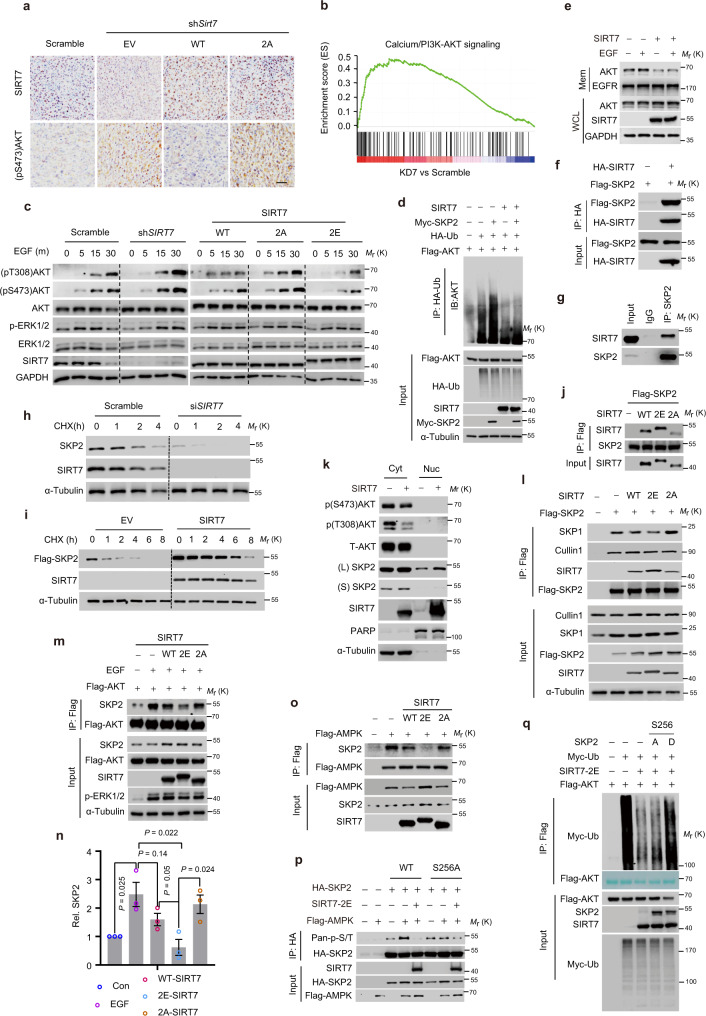
SIRT7 negatively regulates EGF-driven AKT activation. **a** Representative images of IHC staining showing SIRT7 and (pS473)AKT levels in tumor sections from Fig. 4e—tumors derived from murine mammary cancer 4T1 cells with *Sirt7* KD (sh*Sirt7*) and transfected with empty vector (EV), human SIRT7-WT (WT), and SIRT7-2 A (2A), *n* = 3 mice for analysis; scale bar, 50 µm. **b** Gene set enrichment analysis (GSEA) showing enrichment of PI3K/AKT pathways in *SIRT7* knocked down (KD7) BT-549 breast cancer cells; data represent NES = 1.574, nominal *P* = 0.0, FDR *q* = 0.16, determined by Kolmogorov–Smirnov test. **c** Immunoblotting analysis of cellular lysates harvested from MDA-231 breast cancer cells with the indicated manipulations of *SIRT7* expression and EGF (5 ng/ml) treatment; m minutes post-EGF treatment. **d** Ubiquitinated AKT levels assessed in HEK293 cells transfected with the indicated plasmids. **e** Membrane AKT levels assessed in MDA-231 breast cancer cells with or without ectopic *SIRT7* overexpression before and after EGF (5 ng/ml) treatment. Mem membrane protein, WCL whole cellular lysates. **f**, **g** Immunoblotting analysis of immunoprecipitates derived from cell lysates incubated with anti-HA or anti-SKP2 antibodies. **h**, **i** Immunoblotting analysis of SKP2 protein stability determined by CHX chase assays in MDA-231 breast cancer cells with *SIRT7* knockdown and in HEK293 cells transfected as indicated plasmids. **j** Immunoblotting analysis of SIRT7 levels in the anti-FLAG-SKP2 immunoprecipitates. **k** Immunoblots showing cytoplasmic and nuclear levels of SKP2 in MDA-231 breast cancer cells with or without overexpression of SIRT7; L long exposure; S short exposure. **l** Immunoblots showing the protein levels in the anti-FLAG-SKP2 immunoprecipitates derived from protein lysates of HEK293 cells transfected with indicated plasmids. **m**, **n** Immunoblots of anti-FLAG-AKT immunoprecipitates of HEK293 cells transfected with empty vector (EV), human SIRT7-WT (WT), and SIRT7-2A (2A) and with or without EGF (5 ng/ml) treatment. The relevant quantification (*n* = 3 independent experiments) is presented in (**n**), error bars: means ± SEM. **o** Immunoblotting analysis of anti-FLAG-AMPK immunoprecipitates derived from cell lysates of HEK293 cells transfected with the indicated plasmids. **p** Immunoblots showing the levels of phospho-SKP2 by probing with the anti-pan p-S/T antibody in the anti-HA-SKP2 immunoprecipitates of HEK293 cells transfected with the indicated plasmids. **q** Ubiquitinated AKT was evaluated by immunoblotting analysis of anti-FLAG-AKT immunoprecipitates prepared from cell lysates of HEK293 cells transfected with the indicated plasmids. Note: total FLAG-AKT in immunoprecipitates are displayed as membrane stained with Fast Green solution. *P* values were determined by two-tailed Student’s *t*-test. Representative results were obtained from at least three independent experiments. Source data are provided as a Source Data file.

Previous reports showed that PI3K/AKT activation can compromise the anti-tumor effects of dietary restriction^41^. The above data indicate that SIRT7 antagonizes AKT activation (Fig. 1j). Therefore, we reasoned that the GSK3β–SIRT7 axis might inhibit tumor progression via AKT inactivation. Indeed, the *Sirt7* KD cell-derived tumors showed increased (pS473)AKT levels, and this effect was counteracted by SIRT7-WT but not SIRT7-2A (Fig. 5a). In addition, gene set enrichment analysis (GSEA) of the published RNA-seq data^42^ in *SIRT7* KD BT-549 breast cancer cells revealed a significant enrichment in the PI3K/AKT pathway (Fig. 5b). Therefore, we hypothesized that GSK3β-mediated SIRT7 phosphorylation negatively regulates AKT activity. We found that *SIRT7* KD markedly activated AKT, as indicated by the increased (pT308)AKT and (pS473)AKT levels in EGF-stimulated MDA-231 cells (Fig. 5c, left panel). Overexpression of both ectopic SIRT7-WT and SIRT7-2E attenuated AKT activation, while SIRT7-2A did not (Fig. 5c, right panel). This effect seems universal as overexpression of SIRT7-WT and SIRT7-2E also inhibited AKT phosphorylation in HEK293 cells (Supplementary Fig. 6a). We noticed that ERK1/2 activation was barely affected, suggesting independent regulation of AKT by SIRT7. As UBR5 enhanced SIRT7 degradation, we investigated its effects on AKT activity. Indeed, *UBR5* KD markedly attenuated EGF-mediated AKT activation in MDA-231 and BT-549 cells in a SIRT7-dependent manner (Supplementary Fig. 6b–d). Interestingly, although insulin signaling activates AKT^6^, the UBR5–SIRT7 axis had little effect on it (Supplementary Fig. 6e). Recently, an important study showed that SIRT7 deacetylates FKBP51 to promote the PHLPP-mediated dephosphorylation of AKT at Ser473, which sensitizes breast cancer cells to chemotherapy^16^. We found that SIRT7-2A, SIRT7-2E, and SIRT7-WT possessed equivalent ability to deacetylate FKBP51 (Supplementary Fig. 6f), suggesting that SIRT7 T255/S259 phosphorylation does not promote FKBP51 deacetylation. As a scaffold protein, FKBP51 recruits PHLPP to dephosphorylate (pSer473) AKT^43^, whereas the GSK3β–SIRT7 axis simultaneously regulates phosphorylation of AKT at Thr308 and Ser473, thus suggesting the existence of other regulators.

K63-linked polyubiquitination of AKT mediated by SKP2-SCF E3 ligase is crucial for its membrane recruitment and subsequent activation in response to EGF^44^. SIRT7 overexpression prominently decreased K63-linked ubiquitination of AKT (Supplementary Fig. 6g). SKP2-mediated polyubiquitination of AKT was largely inhibited by SIRT7 (Fig. 5d). As a result, the membrane AKT levels were markedly decreased following SIRT7 overexpression (Fig. 5e). Additionally, KD of *SKP2* but not *FKBP51* clearly counteracted the AKT overactivation in *SIRT7* KD cells exposed to EGF (Supplementary Fig. 6h). Taken together, these results indicate that SKP2 plays a critical role in the GSK3β–SIRT7 signaling. We then investigated the mechanism by which SIRT7 regulates SKP2. Initially, we did not observe significant changes in *SKP2* mRNA levels in MDA-231 cells following *SIRT7* KD or overexpression (Supplementary Fig. 6i, j), thus ruling out transcriptional regulation. Interestingly, SKP2 and SIRT7 proteins were easily detected in immunoprecipitates using specific antibodies (Fig. 5f, g), suggesting the existence of a protein complex. SKP2 acetylation, which is critical for its cytoplasmic retention and stabilization, is deacetylated by SIRT3 (refs. ^45,46^). However, SIRT7-WT and relevant mutants, e.g., SIRT7-2A, 2E and the deacetylase-dead mutant (SIRT7-HY), had less effect on SKP2 acetylation (Supplementary Fig. 6k, l), suggesting a deacetylation-independent mechanism. Notably, the overexpression of SIRT7-WT and 2E increased SKP2 protein levels to a greater extent (Supplementary Fig. 6k, l, input). We therefore suspected that SIRT7 phosphorylation stabilizes SKP2. This notion was supported by CHX chase assays showing that SKP2 protein turnover was accelerated by *SIRT7* KD, and stabilized by ectopic SIRT7 overexpression (Fig. 5h, i). We then investigated potential links between SKP2 stabilization and SIRT7 phosphorylation by GSK3β. For that purpose, we compared the stability of SKP2 in cells expressing relevant SIRT7 mutants. With the exception of SIRT7-2A and SIRT7-2A-H187Y, other SIRT7s (SIRT7-WT, -2E, and their related H187Y mutants) all exerted similar ability to stabilize SKP2 (Supplementary Fig. 6m), suggesting that SKP2 is regulated by SIRT7 via a phosphorylation-dependent but deacetylase activity-independent mechanism. Critically, SIRT7-2E had a much greater capacity for binding to SKP2 compared to SIRT7-2A (Fig. 5j), indicating that phosphorylated SIRT7 sequesters, and thus stabilizes SKP2. Similarly, both SKP2 and SIRT7 protein levels were decreased following exposure to EGF (Supplementary Fig. 6n, o), indicating that SKP2 protein turnover is required for EGF-elicited AKT activation, which is at least partially modulated by SIRT7. Interestingly, there was a progressive enrichment of nuclear SIRT7 post-EGF incubation (Supplementary Fig. 6n), suggesting existence of certain mechanisms which might boost the SIRT7 nuclear fraction. Further, as retention of SKP2 in cytoplasm also plays a critical role in AKT activation, we evaluated the cytoplasmic and nuclear distribution of SKP2. Despite significant AKT inhibition, SIRT7 overexpression increased levels of SKP2 in both the cytoplasm and nucleus (Fig. 5k). Thus, it is unlikely that SIRT7-mediated AKT regulation is mediated by disruption of cytoplasmic SKP2 retention. We concluded that SIRT7 phosphorylation by GSK3β enhances its ability to bind and stabilize SKP2, which is detrimental to the EGF-induced SKP2 protein turnover and subsequently compromises AKT activation.

SKP2 is an F-Box protein that forms a complex with SKP1 and Cullin-1 (SKP2-SCF complex) to mediate E3 ligase activity, which catalyzes K63-linked ubiquitination of AKT^44^. Given that the integrity of the complex is critical for E3 ligase activity, we then evaluated the impact of SIRT7 on complex formation. Interestingly, SIRT7-WT apparently compromised the interaction of SKP2 with SKP1 and this effect was greatly enhanced by SIRT7-2E, while SIRT7-2A induced a less marked change (Fig. 5l). Consequently, the binding of SKP2 to AKT was profoundly compromised (Fig. 5m, n), which is consistent with previous observations that SIRT7 prevented AKT K63-linked ubiquitination (Fig. 5d). Another critical issue that requires clarification is why and how the GSK3β–SIRT7 axis specifically targets SKP2. It is known that AMPK phosphorylates SKP2 at S256 to promote the formation and integrity of the SKP2-SCF complex, which is critical for GD-induced AKT activation^27^. We found that GD induced the interaction of SKP2 with AMPK and SIRT7 (Supplementary Fig. 7a). Furthermore, SIRT7-2E, which mimics the hyperphosphorylated state, profoundly inhibited the binding of AMPK to SKP2 (Fig. 5o), suggesting that SIRT7 phosphorylation prevents the phosphorylation of SKP2 by AMPK. In support of this view, we found that SIRT7-2E almost abolished the AMPK-mediated SKP2 phosphorylation at S256, while the effect on the SKP2-S256A mutant was less marked (Fig. 5p). Furthermore, if the GSK3β–SIRT7 axis modulates AKT activity by inhibiting AMPK-mediated SKP2 phosphorylation, the SKP2-S256D mutant, which mimics the phosphorylated state, was expected to restore K63-linked ubiquitination of AKT in the presence of SIRT7-2E. As shown in Fig. 5q, SKP2-S256D greatly counteracted the effect of SIRT7-2E-mediated AKT polyubiquitination inhibition, while SKP2-S256A did not. Thus, we concluded that GD-mediated sequential phosphorylation of SIRT7 by AMPK and GSK3β facilitates its binding to SKP2, which prevents SKP2 S256 phosphorylation by AMPK. Consequently, this compromises the interaction of SKP2 with SKP1 leading to destruction of the integrity of the SCF-SKP2 E3 ligase complex that is critical for K63-linked ubiquitination of AKT, resulting in suppression of AKT activation.

We then aimed to clarify whether these molecular events are functional in physiological activities. HIF1α is a major AKT downstream effector that regulates glycolysis^47^. We found that overexpression of SIRT7-WT and SIRT7-2E, but not SIRT7-2A, impaired the expression of HIF1α and multiple glycolytic genes under hypoxia (Supplementary Fig. 7b, e). Similarly, KD of *SIRT7* or *GSK3β* apparently increased the expression of HIF1α and glycolytic genes (Supplementary Fig. 7c, d). EGF-driven activation of AKT promotes cancer cell motility^6^. In this study, overexpression of SIRT7-WT and SIRT-2E suppressed EGF-induced cell migration, while SIRT7-2A overexpression did not (Supplementary Fig. 7f, g). Similarly, UBR5 silencing suppressed cell migration (Supplementary Fig. 7h). These data indicated that the GSK3β–SIRT7 axis counteracts EGF-mediated AKT activation and regulates cellular functions.

### SIRT7 decline promotes tumor progression

**Fig. 6:**
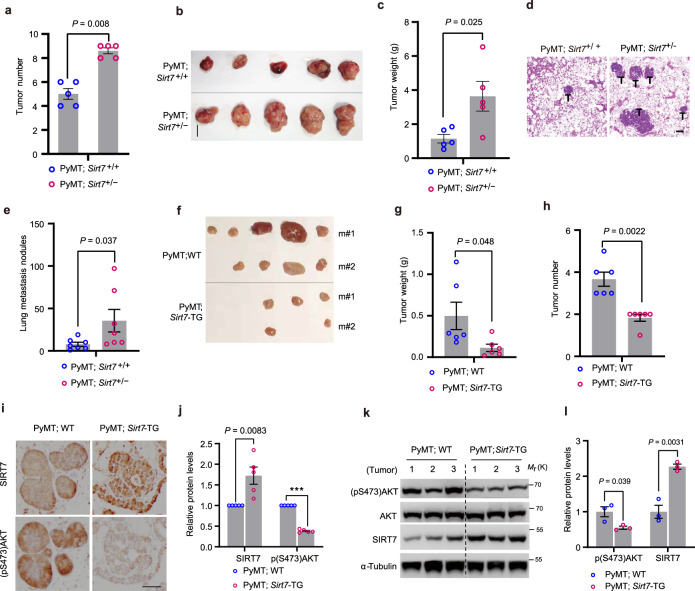
SIRT7 suppresses tumor progression. **a**, **b** Total tumor number (**a**), largest tumor (**b**), and whole tumor weight (**c**) were analyzed in PyMT;*Sirt7*^+/+^ and PyMT;*Sirt7*^+/−^ mice aged 4 months (*n* = 5 mice per group); scale bar, 1 cm. **d** Representative images of H&E staining showing lung metastatic nodules (labeled as T) in PyMT;*Sirt7*^+/+^ and PyMT;*Sirt7*^+/−^ mice, analyzed from *n* = 5 mice per group; scale bar, 500 µm. **e** The number of lung metastatic nodules was counted in H&E-stained whole-lung sections (*n* = 7 mice per group). **f**–**h** Tumor burden (**f**, **g**) and number (**h**) in PyMT;WT and PyMT;*Sirt7*-TG mice (*n* = 6 per genotype). **i**, **j** Representative images showing IHC staining of (pS473)AKT in mammary tumors isolated from PyMT;WT and PyMT;*Sirt7*-TG mice, analyzed from *n* = 5 mice per group. Scale bar, 50 µm. Quantification of the relative expression levels of SIRT7 and (pS473)AKT is shown in (**j**), *n* = 5 mice per group. **k**, **l** Immunoblots (**k**) and related quantification (**l**) showing the (pS473)AKT and SIRT7 levels in tumor samples isolated from PyMT;WT and PyMT;*Sirt7*-TG mice, *n* = 3 mice per group. Data represent means ± SEM (**a**, **c**, **e**, **g**, **h** **j**, **l**). *P* values were calculated by two-tailed Student’s *t*-test. ***P = 0.0000000003. Source data are provided as a Source Data file.

Tumorigenesis is usually accompanied by an imbalance between oncogenic signals and energy stresses^48^. Given that SIRT7-modulated AKT activity, we investigated the role of SIRT7 in tumor initiation via oncogenic EGF/AKT signaling. To that end, we applied transgenic *Sirt7*^+/−^ and *Sirt7*-ΤG mouse lines^42^. *Sirt7*^−/−^ mice suffer from multi-tissue/organ dysfunction, while heterozygous mice are indistinguishable from their WT littermates^18^. We therefore generated the tumorigenic model PyMT;*Sirt7*^+/−^ mice by crossing MMTV (mouse mammary tumor virus)-PyMT (polyoma middle T-antigen) transgenic mice with *Sirt7*^+/−^ mice. MMTV-driven PyMT oncoprotein expression activates the Ras and PI3K/AKT oncogenic pathways to induce mammary tumors^49^. We then analyzed mammary tumors generated in these mice at 4 months of age and the number of tumors in all mammary fat pads of each mouse was counted. Notably, the expression of SIRT7 in PyMT;*Sirt7*^+/−^ mammary tumors was further suppressed (Supplementary Fig. 8a, b, <30% of that in PyMT;*Sirt7*^+/+^ mice), indicating a further loss of SIRT7 in the cancerous context. By contrast, SIRT7 was expressed in the liver of *Sirt7*^+/−^ mice at almost half the level of their *Sirt7*^+/+^ littermates (Supplementary Fig. 8a, b). As a result, PyMT;*Sirt7*^+/−^ mice developed much more numerous primary mammary tumors than their PyMT;*Sirt7*^+/+^ littermates, indicating that loss of *Sirt7* accelerates tumor generation (Fig. 6a). The largest tumors from PyMT;*Sirt7*^+/−^ mice were much larger than those from PyMT;*Sirt7*^+/+^ mice (Fig. 6b). The tumor burden was generally much greater in PyMT;*Sirt7*^+/−^ mice as shown by the greater tumor weight (Fig. 6c). PyMT-induced mammary tumors primarily metastasize to the lung. Compared with PyMT;*Sirt7*^+/+^ mice, PyMT;*Sirt7*^+/−^ mice developed more metastatic foci in the lungs (Fig. 6d, e), supporting previous reports that *Sirt7* loss promotes metastasis^42,50^. In accordance with the hyperactivation of the oncogenic EGF/Ras signaling pathway and the rapid tumor progression, *Vegfa* and *Hif1α* expression was significantly upregulated in PyMT;*Sirt7*^+/−^ tumors (Supplementary Fig. 8c). Furthermore, TPA (12-*O*-tetradecanoyl-phorbol-13-acetate) activates the Ras downstream effector of the mitogen-activated MEK-ERK and AKT signaling pathways. To consolidate our understanding of the role of SIRT7 in oncogenesis, we induced skin carcinogenesis in mice through a two-stage treatment with DMBA (7,12-dimethylbenz (alpha) anthracene) and TPA^51^. Compared with *Sirt7*^+/+^ mice, *Sirt7*^+/−^ mice generated much larger papillomas and in greater numbers following continuous exposure of the dorsal skin to TPA (Supplementary Fig. 9a–c). This suggests that *Sirt7* downregulation favors tumor progression.

We next investigated the ability of *Sirt7* overexpression to suppress tumor growth in *Sirt7*-TG mice, in which ectopic Sirt7 expression was induced by oral administration of doxycycline (DOX, 2 g/l in water). Analysis of mammary tumors generated in PyMT;*Sirt7*-TG mice aged 3 months showed a significant decrease in tumor number and tumor burden compared with those generated in the PyMT;WT littermates (Fig. 6f–h). SIRT7 overexpression was verified by IHC staining and immunoblotting of isolated tumors (Supplementary Fig. 8d, e). Moreover, PyMT;*Sirt7*-TG mammary tumors showed much lower AKT activity (Fig. 6i–l), again supporting the hypothesis that SIRT7 inhibits AKT activation. Moreover, an unbiased analysis of the prognosis of various cancers according to *SIRT7* mRNA level using a public online tool (https://kmplot.com/analysis) revealed that higher *SIRT7* expression was associated with a good prognosis in cancers with EGF/Ras signaling-dependent progression, including bladder, breast, lung, pancreatic, and thyroid cancers and sarcoma (Supplementary Fig. 10a). Similarly, a pattern of decreased SIRT7 expression was observed in higher grade malignant invasive breast cancer samples (Supplementary Fig. 10b, c). Taken together, these data indicated that SIRT7 functions as a tumor suppressor, suggesting the potential anti-tumor benefits of boosting SIRT7 expression.

### Oncogenic EGF signaling hijacks the GSK3β–SIRT7 axis via ERK2

**Fig. 7:**
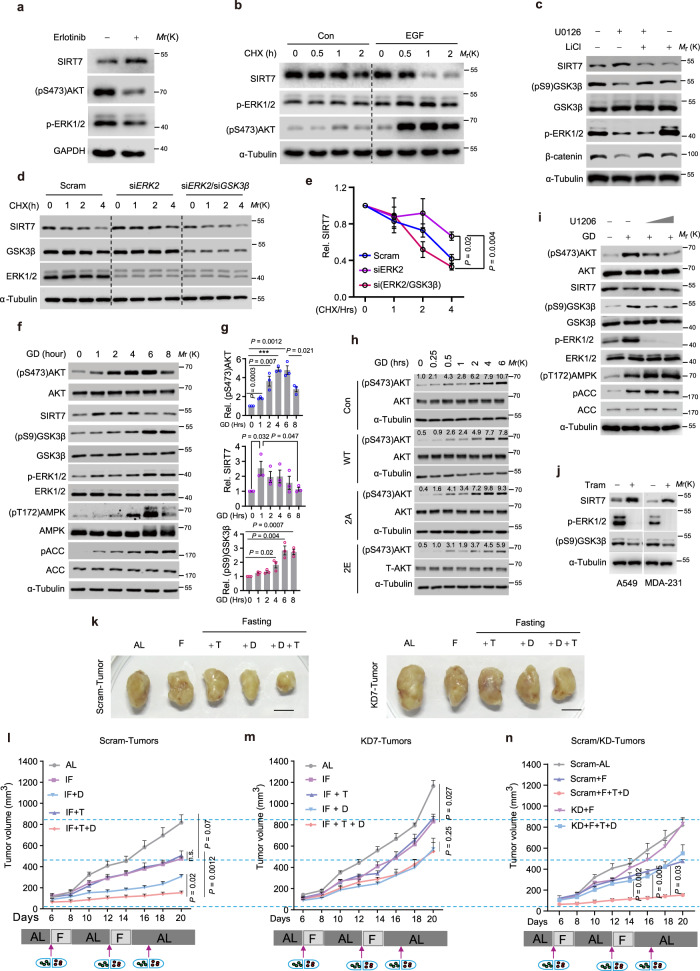
Combining trametinib and fasting enhances the anti-neoplastic efficacy of DXR. **a** Immunoblotting analysis of lysates of MDA-231 breast cancer cells treated with or without erlotinib (20 nM). **b** Immunoblotting analysis of SIRT7 protein stability in MDA-231 breast cancer cells treated with or without EGF (5 ng/ml), determined by CHX (50 µg/ml) chase assay. **c** Immunoblotting analysis of SIRT7 protein levels in MDA-231 cells treated with U0126 (2 µM) or LiCl (10 mM). **d**, **e** Immunoblotting (**d**) analysis of SIRT7 protein stability in MDA-231 cells transfected with the indicated siRNA, determined by CHX (50 µg/ml) chase assay. **e** Curves showing the related quantification, *n* = 3 independent experiments. **f**, **g** Immunoblotting (**f**) analysis of lysates of MDA-231 cells subjected to glucose deprivation (GD) for the indicated periods. Bar charts in (**g**) showed the related quantifications from three independent experiments. **h** Immunoblots showing AKT activation in MDA-231 cells expressing empty vector (EV) and relevant SIRT7 mutants with or without glucose deprivation (GD). **i** Immunoblotting analysis of lysates of MDA-231 cells cultured under glucose deprivation (GD) in the presence or absence of the MEK-ERK inhibitor (ERKi) U0126 (2 µM). **j** Immunoblots showing SIRT7 levels in lung cancer A549 cells and breast cancer MDA-231 cells with or without trametinib (20 nM) treatment. **k**–**n** Representative images (**k**) and growth curves (**l**–**n**) of tumors generated from Scram or *Sirt7* KD 4T1 cells as indicated; Note: all groups of mice in (**l**) and (**m**) were simultaneously manipulated and curves of (**n**) were derived from (**l**) and (**m**). *n* = 5 mice per group in (**l**); n = 4 mice per group in (**m**); F intermittent fasting, T trametinib, D doxorubicin; arrows indicated drug application. Scale bars, 1 cm. Representative results were observed from at least three independent experiments or samples. Data represent means ± SEM (**e**, **g**, **l**–**n**). *P* values were calculated by two-tailed Student’s *t*-test (**e**, **g**) or two-way ANOVA analysis (**l**–**n**), ***P = 0.00003, n.s. no significance. Source data are provided as a Source Data file.

EGF/EGFR signaling inactivates GSK3β via RAS/ERK and/or PI3K/AKT^11^. Our data showing that Sirt7 expression was decreased during tumor progression (Supplementary Figs. 8a and 10) suggest that the GSK3β–SIRT7 axis might be hijacked by oncogenic signaling to facilitate tumor initiation and malignancy. Indeed, a markedly increased SIRT7 expression was observed when EGF signaling activity was blocked in MDA-231 breast cancer cells by erlotinib, whereas EGF stimulation accelerated SIRT7 protein turnover (Fig. 7a, b). EGF treatment markedly increased SIRT7 ubiquitination, and this effect was blocked by *UBR5* KD, leading to restoration of the SIRT7 protein levels following EGF exposure (Supplementary Fig. 11a–c). Similarly, EGF or TPA treatment led to a sharp decrease in p-SIRT7 levels (Supplementary Fig. 11d). Interestingly, treatment with U0126, an inhibitor of ERK1/2 (ref. ^52^), increased SIRT7 protein levels, while wortmannin and SP600125, which inhibits PI3K/AKT and JNK, respectively^53,54^, failed to restore SIRT7 expression (Supplementary Fig. 11e, f). Moreover, U0126 administration increased p-SIRT7 but attenuated the SIRT7 ubiquitination level (Supplementary Fig. 11g, h). Furthermore, *GSK3β* KD and kinase inactivation by LiCl completely abolished the impact of U0126, indicating that the effect of EGF on SIRT7 is GSK3β-dependent (Fig. 7c and Supplementary Fig. 11e, f, i, j). These data suggest that ERK1/2 underlies the mechanism by which EGF promotes SIRT7 degradation.

We next determined the key regulator of the GSK3β–SIRT7 axis by siRNA-mediated KD of each kinase (i.e., si*ERK1*, and si*ERK2*). The EGF-induced downregulation of p-SIRT7 was reduced by ablation of ERK2, but not ERK1 (Supplementary Fig. 12a–d). *GSK3β* KD counteracted the stabilization of SIRT7 induced by ERK2 silencing in MDA-231 breast cancer cells and A549 lung cancer cells (Fig. 7d, e and Supplementary Fig. 12e, f). In addition, *ERK2* KD greatly inhibited the binding of SIRT7 to UBR5 and the EGF-facilitated SIRT7 ubiquitination; these effects were reversed by *GSK3β* KD and LiCl treatment (Supplementary Fig. 12g, h). Thus, these data indicate that EGF/ERK2 signaling substantially determines the integrity of the GSK3β–SIRT7 axis.

### Combined fasting and ERK inhibition enhance the effects of chemotherapy

Fasting inevitably causes acute energy stress in regional tumors, resulting in activation of AKT to protect against apoptosis^27^; therefore, we investigated the role of SIRT7 in this process. SIRT7 protein/phosphorylation levels were initially increased under GD (0‒2 h) and decreased at later time-points, accompanied by sharp increases in ERK1/2 and AKT activation and GSK3β inactivation (Fig. 7f, g and Supplementary Fig. 13a). This suggests that inhibition of the GSK3β–SIRT7 axis is required for AKT activation under acute stress conditions. Indeed, SIRT7-2E overexpression significantly retarded AKT activation under GD (Fig. 7h). We therefore examined whether the GSK3β–SIRT7 axis is crucial for cancer cell survival under long-term GD. To that end, BT-549 breast cancer cells were stably transfected with SIRT7-WT, -2A, -2E, or EV and cultured in glucose-free medium. The viability of BT-549 cells overexpressing SIRT7-WT (33.70 ± 0.37%) or 2E (30.29 ± 10.32%) was significantly lower than the cells overexpressing 2 A (76.79 ± 4.33%) or EV (62.20 ± 1.09%); similar result was obtained in MDA-231 cells (Supplementary Fig. 13b, c). AKT hyperactivation usually dictates chemotherapy resistance in malignant cancers^55^. Interestingly, the genotoxic drug doxorubicin (DXR) suppressed SIRT7 protein and phosphorylation levels in MDA-231 cells, with concomitant AKT activation (Supplementary Fig. 14a, b). Cancer cells overexpressing ectopic SIRT7-WT or SIRT7-2E displayed much higher sensitivity to DXR treatment than SIRT7-2A cells (Supplementary Fig. 14c, d).

These findings indicate that mild energy stress activates the GSK3β–SIRT7 axis, thereby antagonizing tumor growth and metastasis. In contrast, acute energetic stress might boost AKT activation via ERK2-mediated inhibition of the GSK3β–SIRT7 axis, leading to tumorigenesis, malignant disease progression, and drug resistance. Undoubtedly, acute energetic stress restricts the anti-neoplastic efficacy of fasting. Indeed, IF shows limited efficacy against already established tumors^56^. Considering the converging roles of the GSK3β–SIRT7 axis in oncogenic and energetic stresses, combining ERK inhibition might mitigate the impairment of SIRT7 phosphorylation by IF-elicited energy stress. Interestingly, blockade of ERK activity by U1206 restored p-SIRT7 levels and suppressed AKT activation during GD (Fig. 7i and Supplementary Fig. 13d). Furthermore, treatment with the FDA-approved drug trametinib (Tram), a potent MEK1/2 kinase inhibitor indicated for the treatment of melanoma with the BRAF V600E/K mutation^57^, greatly increased SIRT7 levels in A549 cells, MDA-231 cells, and 4T1 cells (Fig. 7j and Supplementary Fig. 13e). Importantly, 4T1 cells were almost eliminated by fasting-mimic treatment combined with Tram and DXR in vitro, indicating a synergistic anti-neoplastic effect (Supplementary Fig. 14e). We further assessed the efficacy of combined therapy comprising fasting and standard DXR plus Tram in vivo. Groups of female Balb/c mice were inoculated with Scram or KD7 4T1 cells as follows: Scram/KD7, Scram/KD7−DXR (4 mg/kg), Scram/KD7−Tram (0.3 mg/kg), and Scram/KD7−DXR+Tram and then subjected to IF or not. For mice bearing tumors derived from Scram 4T1 cells, IF-only achieved a significant inhibition of tumor growth (Scram vs. Scram+IF) and treatment with Tram plus IF had a similar effect (Scram+IF vs. Scram-Tram+IF). DXR significantly facilitated the effect of IF on tumor retardation (Scram-DXR +IF vs. Scram+IF) and strikingly, combined treatment with IF, DXR, and Tram almost completely blocked tumor progress, thus exhibiting the most marked anti-tumor efficacy (Fig. 7k, l). By contrast, tumors derived from *Sirt7* KD cells exhibited significant resistance to this combined therapy regimen (Fig. 7m, n). It was noticeable that single therapy or combined Tram and DXR showed less marked anti-tumor efficacy when mice were fed ad libitum (Supplementary Fig. 14f), highlighting the dominant role of IF in combination therapy. Overall, these results indicate that targeting SIRT7 has great potential to substantially enhance the anti-tumor efficacy of combined chemotherapy and IF.

## Discussion

**Fig. 8:**
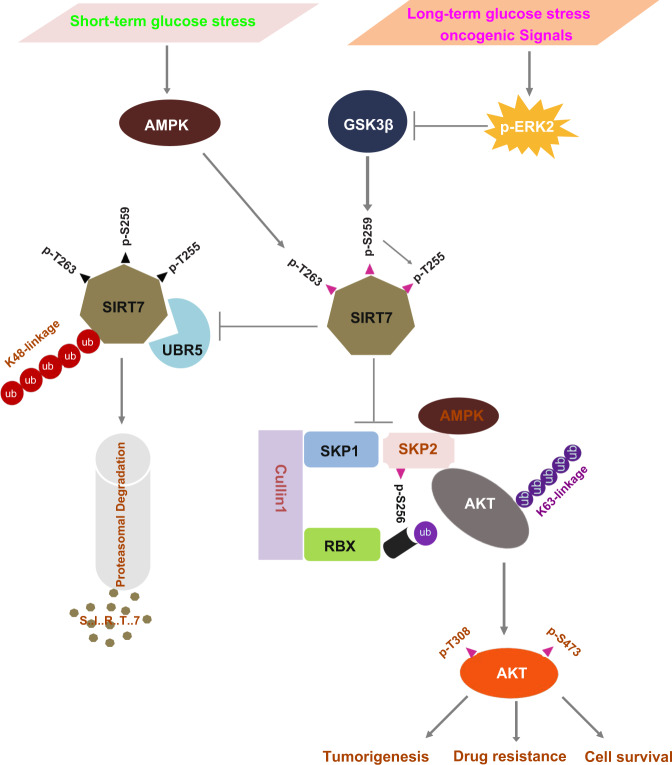
Oncogenic and energetic stress signals converge at the GSK3β–SIRT7 axis. Short term and mild energetic stress activating AMPK to prime the phosphorylation of Thr263 leads SIRT7 to be further phosphorylated at Thr255/Ser259 by GSK3β, which prevents UBR5-mediated SIRT7 K48-polyubiquitination and subsequent proteasomal degradation. As a result, phosphorylated SIRT7 inhibits the integrity of the SCF-SKP2 E3 ligase complex which facilitates AKT K63 polyubiquitination and thus activation. In contrast, long term or extreme energetic stress and/or oncogenic signals hijack the GSK3β–SIRT7 axis by activating ERK2, leading to SIRT7 loss of phosphorylation, and AKT overactivation, which contributes to tumorigenesis, drug resistance, and cell survival. Thus, SIRT7 serves as a central hub that senses oncogenic signals and energetic stress and manipulates malignant progress.

Numerous studies and preclinical data as well as several on-going clinical trials have provided solid evidence that fasting or a fasting-mimic diet is a promising intervention that can achieve broad tumor regression^56,58,59^. However, fasting shows limited efficacy against established tumors^56^, and might cause acute regional energy stress, which rather activates AKT to promote cancer cell survival^27^ and apparently restricts the anti-neoplastic efficacy of fasting. Therefore, understanding the molecular events underlying the benefit of fasting and through which cancer cells manipulate the challenges of both oncogenic signal and energetic stress merits in-depth investigation. In this study, we show that fasting leads to energetic stress that activates the metabolic regulators AMPK and GSK3β. AMPK phosphorylates SIRT7 at T263 to prime subsequent phosphorylation at T255/S259 by GSK3β, decoupling SIRT7 from UBR5 E3 ligase, and thereby preventing K48-linked polyubiquitination and proteasomal degradation of SIRT7. SIRT7 phosphorylation by GSK3β disrupts the integrity of the SCF-SKP2 E3 ligase complex that mediates K63 polyubiquitination and activation of AKT. Unfavorably, energetic stress and/or oncogenic signals hijack the GSK3β–SIRT7 axis, leading to AKT overactivation, and thus, tumorigenesis, drug resistance, and cell survival. Our data highlight SIRT7 as a central hub on which energetic and oncogenic signals converge to prevent malignant transformation and underpin the anti-neoplastic effect (Fig. 8).

Aberrant regulation of growth signaling is a hallmark of cancer that often occurs through activation of growth factor receptors or their downstream effectors^60^. The identification of genes involved in oncogenic signaling pathways is essential for new targeted therapeutics, especially considering the frequent occurrence of drug resistance when targeting overactivated oncogenic receptors and kinases. Our findings shed light on the role of SIRT7 in antagonizing tumorigenesis through the suppression of oncogenic EGF-driven AKT activation. We hypothesize that targeting SIRT7 might be beneficial for treating EGF signaling-dependent cancers, e.g., triple-negative breast cancers with increased EGFR expression and activity, colorectal and pancreatic cancers that harbor Ras mutations and NSCLCs with EGFR mutations. In accordance with the hypothesis, we observed that SIRT7 overexpression suppressed the growth of xenografted H1975 lung cancer cells, which possess the constitutively activated EGFR L858R/T790M mutation. As the point at which EGF signaling and the energetic stress response converge, the GSK3β–SIRT7 axis represents an ideal therapeutic target in various cancers. Indeed, anti-tumor efficacy is optimized by the combination of trametinib, doxorubicin, and fasting. Mechanistically, trametinib blocks oncogenic EGF signaling and concomitantly ensures the integrity of the GSK3β–SIRT7 axis, which guarantees subsequent activation by fasting and enhances the efficacy of DXR treatment.

Although the synergistic effects of combining fasting with chemotherapy have been confirmed for clinical agents such as metformin^8^, vitamin C^61^, and hormone therapy^9^, certain cancers are tolerant to the anti-neoplastic effects of fasting due to distinct signaling characteristics. For instance, tumors with constitutive PI3K activation are resistant to dietary restriction^41^, and colorectal cancers without KRAS mutation show less significant synergized efficacy of the combination of fasting and vitamin C^61^. Thus, the critical challenges to further potentiation of the efficacy of fasting are to identify tumors for which the greatest benefits of fasting can be achieved and to elucidate the underlying molecular mechanisms. In this study, we reveal that SIRT7 may serve as a molecular marker, with high expression being associated with a better response to a fasting regimen. Indeed, SIRT7 missense and insertion mutations were identified in the region containing the AMPK and GSK3β motifs (COSMIC database).

Our study reveals the function of SIRT7 as a hub on which the key energy regulators AMPK and GSK3β converge. Under energetic stress conditions, AMPK and GSK3β cooperatively and sequentially phosphorylate SIRT7, resulting in long-term SIRT7 stabilization. AMPK also phosphorylates SIRT7 at T153 to modulate rDNA transcription^17^. Furthermore, SIRT7 R388 hypomethylation is coupled to AMPK activation, which regulates mitochondrial biogenesis, but not rDNA transcription in response to glucose availability^15^. GSK3β phosphorylates a series of substrates, including β-catenin and Myc, resulting in degradation and/or inactivation, and thereby inhibiting cancer cell proliferation and metastasis^62^. GSK3β also promotes cell survival by activating multiple pro-proliferative pathways or factors, including NF-κB, mTOR, and C/EBPβ^63^. Thus, the role of GSK3β in malignancy is determined by the context of substrates as well as tumor types. Recently, an elegant study demonstrated that the combination of fasting and metformin impairs tumor growth by boosting GSK3β activity^8^. This is consistent with our finding that GSK3β acts as a tumor suppressor, especially in the earlier stage of tumorigenesis, by stabilizing SIRT7 to suppress AKT activation. Interestingly, the roles of SIRT7 in tumor development are also hotly debated. Although accumulating evidence shows that SIRT7 expression is increased in many subtypes of cancers, and SIRT7 ablation suppresses tumor growth^64^, many studies have also shown that SIRT7 loss promotes malignant progression through metastasis^42,50^, resistance to chemotherapy^16,65^, and tumor survival under energy stress^16,17^. SIRT7 overexpression inhibits HIF1/2α and Myc activity^21,66^. Considering the substantial oncogenic characteristics of HIF and Myc in malignant cancers^67^, this strongly indicates that SIRT7 negatively regulates tumorigenesis. We attribute these apparent inconsistencies to the diversely oncogenic pathways and lack of a relevant animal tumor model, with current studies focusing mainly on cancer cell lines, which ignore the intratumoral complexity. Using transgenic tumor models, we show that SIRT7 insufficiency promotes the initiation, progression, and metastasis of breast and skin tumors. In contrast, SIRT7 overexpression significantly attenuates breast tumor development and lung metastasis. At the molecular level, SIRT7 suppresses SCF-SKP2 E3 ligase-mediated AKT ubiquitination, thus antagonizing the function of AKT in tumor growth and distal organ metastasis. Of note, a previous study showed that SIRT7 deacetylates FKBP51 and uniquely suppresses AKT S473 activity^16^. Another study which supports our conclusion showed that *Sirt7* loss results in AKT hyperphosphorylation at both S473 and T308 (ref. ^68^), although the mechanism was not clarified. This suggests that the SIRT7–AKT axis is regulated by multiple mechanisms in different signaling contexts.

As an E3 ligase, SKP2 possesses amounts of substrates, regulating multiple aspects of tumors, including cell proliferation, metabolic reprogramming, and malignant metastasis^69^. Our finding that SIRT7 confers SKP2 stabilization further complicates the abovementioned debates on the roles of SIRT7 in tumorigenesis. As a bona fide substrate, the degradation of P27 in nucleus emphasizes SKP2 as a tumor driver, by which it facilitates cell cycle entry and proliferation in many cancers^70,71^. Several studies found that SIRT7 overexpression decreases P27 level and accelerates cell proliferation^72,73^, supporting SIRT7 as an SKP2 related oncogenic driver. During tumor progression, the majority of SKP2 protein is translocated to cytoplasm, while the function has not been well addressed^70^. We found that SIRT7 compromises SKP2-mediated AKT activation in the cytoplasm, thus inhibiting malignant tumor progression. Therefore, in future study, it would be worthwhile to thoroughly evaluate the role of SIRT7 subcellular distribution when considering its regulation on distinct substrates. Interestingly, SIRT6, another sirtuin member, can deacetylate SKP2 on K73 and K77 to enhance its further phosphorylation S72 and S75 mediated by AKT and CK1, respectively, thus preventing the APC-Cdh1 E3 ubiquitin-ligase-mediated degradation^74,75^. In our study, though we did not observe an obvious deacetylation of SKP2 by SIRT7, in future study, it would be interesting to evaluate whether SIRT7 also contributes to SKP2 protein stability via APC-Cdh1 E3 ubiquitin-ligase.

SIRT7 is a member of the pro-longevity sirtuin family. Recently, several studies have demonstrated that SIRT7 expression is decreased during aging and SIRT7 loss accounts for age-related diseases, such as osteopenia^22^, vascular dysfunction^68^, cardiac hypertrophy^76^, and stem cell aging^23^. Therefore, it can be speculated that enhancing the activity or expression level of SIRT7 will be beneficial in ameliorating age-associated diseases. Since cancer is widely recognized as an age-related disease and the incidence of most cancers increases with age^77^, the roles of SIRT7 in cancers should be clearly elucidated if considering its further therapeutic application for aging. We show that SIRT7 suppresses breast/skin tumor development, thus indicating that targeting SIRT7 might have the dual benefits of anti-tumor activity and extended healthy lifespan. Importantly, given that mild fasting results in energy stress and a decline in the levels of growth factors, leading to increased AMPK and GSK3β activity, it can be predicted that fasting would boost SIRT7 functions in normal tissues/organs, as has been reported previously in liver^20^. More importantly, caloric restriction (CR) is the only widely accepted manipulation that brings overall health benefits in most species. Therefore, a comprehensive understanding of the contribution of SIRT7 to the effects of CR is vital.

In this study, we have revealed the GSK3β–SIRT7 axis that must be fine-tuned in response to energetic and oncogenic stresses in malignancy. We provide evidence that the anti-tumor efficacy of doxorubicin is enhanced by combination therapy with the MEK inhibitor trametinib, which strengthens GSK3β activity, thus blocking oncogenic signaling. Fasting coordinates AMPK and GSK3β activity to ensure the stabilization of SIRT7, which attenuates AKT activity by disrupting the integrity of the SCF-SKP2 E3 ligase complex. Our data highlight the potential benefits of targeting SIRT7 using small-molecule activators in cancer therapy and age-related diseases.

## Methods

### Reagents and antibodies

The antibodies used in this study are listed in Supplementary Table 1. Trametinib (HY-10999) was purchased from MCE Technologies, USA. CHX (No. 0970) and MG132 (No. 1748/5) were from TOCRIS Bioscience, UK. AICAR (S1802), A-769662 (S2697), and CC (S7306) were from Selleck, USA. LiCl (A100416) was obtained from Sangon Biotech (Shanghai, China). Puromycin (P8833), Pierce™ Protein A/G plus agarose (20423), and Pierce™ GST agarose (20211) were from Thermo Fisher, USA. Anti-FLAG-beads (F2426) and anti-HA-beads (IP0010) were purchased from Sigma-Aldrich, USA. Recombinant human GSK3β protein (active) (ab60863) was purchased from Abcam, UK.

### Cell culture and lentivirus generation

HeLa, HEK293, MDA-MB-231, MCF-7, and A549 cells were cultured in Dulbecco’s modified Eagle’s medium (DMEM, high glucose) supplemented with 10% fetal bovine serum (FBS, PAN-Biotech GmbH, Germany). H1975 and BT-549 cells were maintained in RPMI-1640 supplemented with 10% FBS (PAN-Biotech GmbH). Cell lines were routinely tested for mycoplasma contamination detected by PCR (TaKaRa, Japan). Transfection of plasmid/siRNA was performed using Lipo3000^®^ (Thermo Fisher) according to the manufacturer’s instructions. To generate lentivirus for the generation of the indicated stable cell lines, HEK293 cells were plated in 10-cm dishes and transfected with lentiviral plasmids and the lentiviral packaging plasmids pMD2.G and psPAX2 at a ratio of 1:0.5:1. At 48 h after transfection, the supernatant was collected, filtered (0.22 µm filter, Millipore, USA), and later used to infect the indicated cells. At 24 h after infection, cells were further passaged for 1–2 generation and then selected by culture in the presence of puromycin (1 µg/ml) or sorted for green fluorescent protein-positive cells (BD FACSMelody™ Cell Sorter, USA).

### Knockdown of gene expression by shRNA and siRNA

Lentiviral shRNA plasmids and siRNA oligos were ordered from GenePharma Company (Shanghai, China). The shRNA and siRNA sequences are as follows: shRNA against human *SIRT7*-1, 5′-TAGCCATTTGTCCTTGAGGA-3′; shRNA against human *SIRT7*-2, 5′-CACCTTTCTGTGAGAACGGAA-3′; siRNA against human *SIRT7* siRNA-1, 5′-CUCACCGUAUUUCUACUACUAdTdT-3′; siRNA against human *SIRT7* siRNA-2, 5′-CACCUUUCUGUGAGAACGGAAdTdT-3′; shRNA against mouse *Sirt7*-1, 5′-TGCATCCCTAACAGAGAGTAT-3′; shRNA against mouse *Sirt7*-2, 5′-CCTCCCTCTTTCTACTCCTTA-3′; siRNA against human *GSK3β*-1, 5′-GGACAAGAGAUUUAAGAAUdTdT-3′; siRNA against human *GSK3β*-2, 5′-GUAUUGCAGGACAAGAGAUdTdT-3′; siRNA against human *UBR5*-1, 5′-CAACUUAGAUCUCCUGAAAdTdT-3′; siRNA against human *UBR5*-2, 5′-AGACAAAUCUCGGACUUGAdTdT-3′; siRNA against human ERK1, 5′-CGUCUAAUAUAUAA-AUAUAdTdT-3′; siRNA against human ERK2, 5′- GUUCGAGUAGCUAUCAAGAdTdT-3′; siRNA against human MEK1, 5′- GUGAAUAAAUGCUUAAUAAdTdT-3′; siRNA against human MEK2, 5′- GCAUUUGCAUGGAACACAUdTdT-3′; siRNA against human *GSK3α*-1, 5′-GUUCAAGUUCCCUCAGAUUAA-3′; siRNA against human *GSK3α*-2, 5′- ACUAGAGGGCAGAGGUAAAU-3′.

### Plasmid constructs

FLAG-UBR5 (#37188), HA-GSK3β (#14753), HA-GSK3β-S9A (#14754), and HA-GSK3β-K85A expression plasmids were from Addgene (USA). FLAG-AMPKα1 (CH805185), FLAG-SKP2 (CH896343), and FLAG-AKT1 (CH846646) were purchase from Vigenebio (Shangdong, China). SIRT7 and GSK3β deletion mutants were constructed into the p3× FLAG-CMV10 vector (Sigma). GST-tagged SIRT7 or SIRT7-S259A/Thr263A/Thr255A was constructed by cloning into pGEX-4T-3 (GE Healthcare). The other SIRT7 mutants were generated by the site-directed mutagenesis using the KOD-Plus-Neo DNA polymerase (TOYOBO). All plasmids were confirmed by DNA sequencing (Genewiz). Details of the primers used for the generation of these plasmids or other experiments are shown in Supplementary Table 2.

### RNA extraction and quantitative PCR analysis

Total RNA was extracted from cells using TRIzol^®^ (TaKaRa) and then reverse-transcribed into cDNA using the PrimeScript RT-PCR Kit (TaKaRa, RR014) according to the manufacturer’s instructions. The cDNA products were used for quantitative PCR analysis by SYBR Mix (TaKaRa, RR064). Details of the sequences of the primers used are presented in Supplementary Table 2.

### Protein extraction, immunoprecipitation, and immunoblotting

Whole-cell proteins were extracted using immunoprecipitation lysis buffer (25 mM Tris-HCl pH 7.4, 150 mM NaCl, 1% NP-40, 1 mM EDTA) containing a cocktail of protease and phosphatase inhibitors (Sigma, complete™ Protease Inhibitor Cocktail and PhosSTOP™). Immunoprecipitation and immunoblotting with the appropriate antibodies were performed as described in Supplementary Table 1. Image Lab Software (Bio-Rad, V5.2.1 build 11) was used for immunoblotting analysis. Image J software (V1.52) was used for quantification analysis. Uncropped and unprocessed blots are presented in the Supplementary Information file.

### GST-tagged SIRT7 protein purification

GST-SIRT7-WT, GST-SIRT7-T255A, GST-SIRT7-S259A, and GST-SIRT7-T255A/S259A in the bacterial expression plasmid pGEX-4T-3 were expressed in *Escherichia coli* BL21 following induction by 0.1 mM isopropyl β-d-1-thiogalactopyranoside at 25 °C. Bacterial pellets were resuspended in GST-binding buffer (25 mM Tris-HCl pH 7.5, 150 mM NaCl, 1 mM EDTA) containing protease inhibitors. The proteins were purified using glutathione agarose according to the manufacturer’s protocol.

### In vitro kinase assay

To prepare the AMPK kinase complex, FLAG-AMPK was transfected into HEK293 cells which were treated with AICAR (10 mM) for 12 h to stimulate AMPK activation and then immunoprecipitated (IPed) using anti-FLAG agarose beads according to the manufacturer’s instructions. The in vitro kinase assays were conducted in a kinase reaction buffer (50 mM Tris-HCl pH 7.4, 1 mM dithiothreitol (DTT), 10 mM MgCl_2_, 0.5 M ATP) containing the IPed AMPK complex and active GSK-3β (100 ng) plus 2 µg soluble various forms of GST-SIRT7 proteins. Reactions were incubated at 30 °C for 1 h and then terminated by the addition of sodium dodecyl sulfate (SDS) sample buffer. The phosphorylated SIRT7 was assessed by western blotting.

### In vivo ubiquitylation assay

HEK293 cells were co-transfected with the indicated plasmids or siRNA oligos for 48 h, and then treated with relevant activators or inhibitors. Briefly, cells were stimulated with EGF for 6 h or pre-treated with inhibitors for 1 h before further treatment. Cells were lysed using RIPA lysis buffer (50 mM Tris-HCl pH 7.4, 150 mM NaCl, 1% NP-40, 0.5% deoxycholic acid, 0.1% SDS, 10 mM NaF, 1 mM DTT, 0.2 mM Na_3_VO_4_, 1 mM phenylmethylsulfonyl fluoride, and a protease/phosphatase inhibitor cocktail). Cell lysates were immunoprecipitated using the indicated antibodies and washed three times with RIPA buffer. Immunoprecipitates were finally eluted with SDS sample buffer and subjected to immunoblotting analysis.

### Cell viability assay

4T1 and MDA-MB-231 cells (1 × 10^4^) were seeded on 96-well plates for the indicated treatments. Cell viability was analyzed by using Cell Counting Kit-8 (CCK8) (MCE, HY-K0301) according to the manufacturer’s instructions.

### Collective migration

For wound-healing assays, which evaluate the collective migration ability, NCI-H1975 cells were cultured in 12-well plates coated with 0.1% gelatin in DMEM supplemented with 10% FBS. When the cells reached 70% confluence, cells were starved overnight before a scratch (wound) was introduced at the center of the cell monolayer using a sterile plastic pipette tip. Debris was removed by washing with PBS and cells were cultured in fresh complete medium for 12 h. The wound was photographed at the indicated times.

### Transwell migration assays

Cell motility in response to EGF was evaluated in Transwell migration assays. Cells were first cultured for 24 h in FBS-free medium containing 10 ng/ml EGF or not. Subsequently, 2 × 10^4^ cells suspended in 200 µl FBS-free medium were placed in the upper insert (8 µm pore size, Corning) of a 24-well Transwell plate and 500 µl 2% FBS medium was added to the lower chamber as a chemoattractant. After incubation for 4–6 h, the inner membrane of the insert was wiped with cotton swabs to remove the non-migrated cells, and the migrated cells were stained with 0.1% crystal violet. The number of migrated cells was counted to evaluate the migration ability.

### DMBA/TPA-induced skin carcinogenesis in mice

The dorsal hair of *Sirt7*^+/+^ and *Sirt7*^+/−^ mice (aged 6 weeks) was shaved 2 days before the topical application of 200 nM DMBA (in 200 μl acetone). One week later, 20 nM TPA (dissolved in 50 μl acetone) was applied topically every 3 days for 35 weeks. Finally, the number of papillomas per mouse was recorded and the related size was measured using Vernier calipers. Mice were maintained at 21–23 °C, in 40–60% humidity, and with a 12 h light/12 h dark light cycle. Experimental mice were housed and handled in accordance with protocols approved by the Committee on the Use of Live Animals in Teaching and Research of Shenzhen University (China).

### Murine mammary cancer models

FVB/N-Tg (MMTV-PyMT)/Nju mice were purchased from the Model Animal Research Center of Nanjing University (China). Tet-on *Sirt7* transgenic mice (*Sirt7*-TG) or *Sirt7* knockout mice were obtained as described in our published work^42^. PyMT mice were crossed with *Sirt7*-TG mice to obtain PyMT;WT and PyMT;*Sirt7*-TG females or *Sirt7*^+/−^ mice to generate PyMT;*Sirt7*^+/+^ and PyMT;*Sirt7*^+/−^ mice. To achieve Sirt7 overexpression, PyMT;*Sirt7*-TG mice were fed with doxycycline (2 mg/ml) in the drinking water from the age of 3 weeks. At the end of experiment, all mice were sacrificed and the tumor volume, weight, and extent of lung metastasis were recorded. Mice were kept at 21–23 °C, in 40–60% humidity, and with a 12 h light/12 h dark light cycle. Mice were housed and handled in accordance with protocols approved by the Committee on the Use of Live Animals in Teaching and Research of Shenzhen University (China).

### In vivo xenograft model

Pathogen-free female BALB/c mice (aged 5–6 weeks) were purchased from the Shanghai Laboratory Animal Center, CAS (Shanghai, China). The fourth mammary fat pad of the mice was injected with 4T1 cells (5 × 10^5^). Tumor diameters were measured and the tumor volume (mm^3^) was calculated according to the formula: volume = 0.5 × length × width^2^. The lung metastatic nodules were counted after hematoxylin and eosin (H&E) staining of the whole lung. Mice were maintained at 21–23 °C, in 40–60% humidity, and with a 12 h light/12 h dark light cycle. Experimental animals were housed and handled in accordance with protocols approved by the Committee on the Use of Live Animals in Teaching and Research of Shenzhen University (China).

### Immunohistochemical analysis of human breast cancer tissues

Human breast cancer tissue arrays (BR963c) were bought from Alena bio (Shanxi, China). Pathological diagnosis was based on the manufacturer’s instructions. The immunohistochemical (IHC) staining of tissues was scored as negative (0) when <10% of tumor cells showed expression. Positive scores (1+ to 3+) were assigned according to the percent of tumor cells and positive staining intensity. In the Chi-squared analysis, scores were defined as follows: low = 0/1+, medium = 2+, high = 3+.

### Statistical analysis

Excel (Microsoft Office) were used for statistical analyses. Results are based on at least three independent experiments for most experiments. The data are presented as the mean ± standard error of the mean (SEM) or the mean ± standard deviation (SD) as indicated in the figure legends. The statistical *P* values were determined by Student’s *t*-test, two-way ANOVA analysis or Chi-squared test as indicated in the manuscript.

### Reporting summary

Further information on research design is available in the Nature Research Reporting Summary linked to this article.

## Supplementary information

Supplementary Information

Reporting Summary

## Data Availability

The survival analysis of cancer patients is based on a public online tool (https://kmplot.com/analysis). The RNA-sequencing data for GSEA analysis are available in GEO with the accession code GSE99596. SIRT7 phosphorylation sites by GSK3β is predicated by GPS 5.0 (http://gps.biocuckoo.cn/). SIRT7 mutations are identified in the COSMIC database (https://cancer.sanger.ac.uk/cosmic). Other data supporting the findings of this study are available in the main figures, supplementary information, and the source data file. Source data are provided with this paper.

## Source data

Source Data

## References

[1] Forster JC, Harriss-Phillips WM, Douglass MJ, Bezak E (2017). A review of the development of tumor vasculature and its effects on the tumor microenvironment. Hypoxia (Auckl.).

[2] Finicle BT, Jayashankar V, Edinger AL (2018). Nutrient scavenging in cancer. Nat. Rev. Cancer.

[3] Zhao Y (2017). ROS signaling under metabolic stress: cross-talk between AMPK and AKT pathway. Mol. Cancer.

[4] Lin SC, Hardie DG (2018). AMPK: sensing glucose as well as cellular energy status. Cell Metab..

[5] Saha M (2018). AMPK-Akt double-negative feedback loop in breast cancer cells regulates their adaptation to matrix deprivation. Cancer Res..

[6] Song M, Bode AM, Dong Z, Lee MH (2019). AKT as a therapeutic target for cancer. Cancer Res.

[7] Lee C, Longo VD (2011). Fasting vs dietary restriction in cellular protection and cancer treatment: from model organisms to patients. Oncogene.

[8] Elgendy M (2019). Combination of hypoglycemia and metformin impairs tumor metabolic plasticity and growth by modulating the PP2A-GSK3beta-MCL-1 axis. Cancer Cell.

[9] Caffa I (2020). Fasting-mimicking diet and hormone therapy induce breast cancer regression. Nature.

[10] Cipriano R (2012). FAM83B mediates EGFR- and RAS-driven oncogenic transformation. J. Clin. Invest..

[11] Doble BW, Woodgett JR (2003). GSK-3: tricks of the trade for a multi-tasking kinase. J. Cell Sci..

[12] Sutherland C (2011). What are the bona fide GSK3 substrates?. Int. J. Alzheimers Dis..

[13] Frame S, Cohen P, Biondi RM (2001). A common phosphate binding site explains the unique substrate specificity of GSK3 and its inactivation by phosphorylation. Mol. Cell.

[14] Ford E (2006). Mammalian Sir2 homolog SIRT7 is an activator of RNA polymerase I transcription. Genes Dev..

[15] Yan, W. W. et al. Arginine methylation of SIRT7 couples glucose sensing with mitochondria biogenesis. *EMBO Rep*. **19**, 10.15252/embr.201846377 (2018). 10.15252/embr.201846377 PMC6280788 30420520

[16] Yu J (2017). Regulation of serine-threonine kinase Akt activation by NAD(+)-dependent deacetylase SIRT7. Cell Rep..

[17] Sun L (2016). Regulation of energy homeostasis by the ubiquitin-independent REGgamma proteasome. Nat. Commun..

[18] Vazquez BN (2016). SIRT7 promotes genome integrity and modulates non-homologous end joining DNA repair. EMBO J..

[19] Yoshizawa T (2014). SIRT7 controls hepatic lipid metabolism by regulating the ubiquitin-proteasome pathway. Cell Metab..

[20] Ryu D (2014). A SIRT7-dependent acetylation switch of GABPbeta1 controls mitochondrial function. Cell Metab..

[21] Shin J (2013). SIRT7 represses Myc activity to suppress ER stress and prevent fatty liver disease. Cell Rep..

[22] Fukuda M (2018). SIRT7 has a critical role in bone formation by regulating lysine acylation of SP7/Osterix. Nat. Commun..

[23] Mohrin M (2015). Stem cell aging. A mitochondrial UPR-mediated metabolic checkpoint regulates hematopoietic stem cell aging. Science.

[24] Chen S (2013). Repression of RNA polymerase I upon stress is caused by inhibition of RNA-dependent deacetylation of PAF53 by SIRT7. Mol. Cell.

[25] Tang M (2019). SIRT7-mediated ATM deacetylation is essential for its deactivation and DNA damage repair. Sci. Adv..

[26] Li L (2016). SIRT7 is a histone desuccinylase that functionally links to chromatin compaction and genome stability. Nat. Commun..

[27] Han F (2018). The critical role of AMPK in driving Akt activation under stress, tumorigenesis and drug resistance. Nat. Commun..

[28] Lee C (2012). Fasting cycles retard growth of tumors and sensitize a range of cancer cell types to chemotherapy. Sci. Transl. Med.

[29] Hopkins BD (2018). Suppression of insulin feedback enhances the efficacy of PI3K inhibitors. Nature.

[30] Zhou G (2001). Role of AMP-activated protein kinase in mechanism of metformin action. J. Clin. Invest.

[31] Fiol CJ, Mahrenholz AM, Wang Y, Roeske RW, Roach PJ (1987). Formation of protein kinase recognition sites by covalent modification of the substrate. Molecular mechanism for the synergistic action of casein kinase II and glycogen synthase kinase 3. J. Biol. Chem..

[32] Stambolic V, Ruel L, Woodgett JR (1996). Lithium inhibits glycogen synthase kinase-3 activity and mimics wingless signalling in intact cells. Curr. Biol..

[33] Inoki K, Zhu T, Guan KL (2003). TSC2 mediates cellular energy response to control cell growth and survival. Cell.

[34] Blank MF, Grummt I (2017). The seven faces of SIRT7. Transcription.

[35] Kiran S (2013). Intracellular distribution of human SIRT7 and mapping of the nuclear/nucleolar localization signal. FEBS J..

[36] Barber MF (2012). SIRT7 links H3K18 deacetylation to maintenance of oncogenic transformation. Nature.

[37] Tsai YC, Greco TM, Boonmee A, Miteva Y, Cristea IM (2012). Functional proteomics establishes the interaction of SIRT7 with chromatin remodeling complexes and expands its role in regulation of RNA polymerase I transcription. Mol. Cell Proteomics.

[38] Jiang W (2011). Acetylation regulates gluconeogenesis by promoting PEPCK1 degradation via recruiting the UBR5 ubiquitin ligase. Mol. Cell.

[39] Wang Z (2019). AKT drives SOX2 overexpression and cancer cell stemness in esophageal cancer by protecting SOX2 from UBR5-mediated degradation. Oncogene.

[40] Jiang L (2017). Ubiquitin-specific peptidase 7 (USP7)-mediated deubiquitination of the histone deacetylase SIRT7 regulates gluconeogenesis. J. Biol. Chem..

[41] Kalaany NY, Sabatini DM (2009). Tumours with PI3K activation are resistant to dietary restriction. Nature.

[42] Tang X (2017). SIRT7 antagonizes TGF-beta signaling and inhibits breast cancer metastasis. Nat. Commun..

[43] Wang L (2011). FKBP51 regulation of AKT/protein kinase B phosphorylation. Curr. Opin. Pharmacol..

[44] Chan CH (2012). The Skp2-SCF E3 ligase regulates Akt ubiquitination, glycolysis, Herceptin sensitivity, and tumorigenesis. Cell.

[45] Inuzuka H (2012). Acetylation-dependent regulation of Skp2 function. Cell.

[46] Zhang S (2017). Hippo signaling suppresses cell ploidy and tumorigenesis through Skp2. Cancer Cell.

[47] Meijer TW, Kaanders JH, Span PN, Bussink J (2012). Targeting hypoxia, HIF-1, and tumor glucose metabolism to improve radiotherapy efficacy. Clin. Cancer Res..

[48] Ying H (2012). Oncogenic Kras maintains pancreatic tumors through regulation of anabolic glucose metabolism. Cell.

[49] Pylayeva Y (2009). Ras- and PI3K-dependent breast tumorigenesis in mice and humans requires focal adhesion kinase signaling. J. Clin. Invest..

[50] Li W, Zhu D, Qin S (2018). SIRT7 suppresses the epithelial-to-mesenchymal transition in oral squamous cell carcinoma metastasis by promoting SMAD4 deacetylation. J. Exp. Clin. Cancer Res.

[51] Abel EL, Angel JM, Kiguchi K, DiGiovanni J (2009). Multi-stage chemical carcinogenesis in mouse skin: fundamentals and applications. Nat. Protoc..

[52] Favata MF (1998). Identification of a novel inhibitor of mitogen-activated protein kinase kinase. J. Biol. Chem..

[53] Bennett BL (2001). SP600125, an anthrapyrazolone inhibitor of Jun N-terminal kinase. Proc. Natl Acad. Sci. USA.

[54] Liu Y (2005). Wortmannin, a widely used phosphoinositide 3-kinase inhibitor, also potently inhibits mammalian polo-like kinase. Chem. Biol..

[55] Wendel HG (2004). Survival signalling by Akt and eIF4E in oncogenesis and cancer therapy. Nature.

[56] Nencioni A, Caffa I, Cortellino S, Longo VD (2018). Fasting and cancer: molecular mechanisms and clinical application. Nat. Rev. Cancer.

[57] Flaherty KT (2012). Combined BRAF and MEK inhibition in melanoma with BRAF V600 mutations. N. Engl. J. Med..

[58] Vernieri C, Ligorio F, Zattarin E, Rivoltini L, de Braud F (2020). Fasting-mimicking diet plus chemotherapy in breast cancer treatment. Nat. Commun..

[59] de Groot S (2020). Fasting mimicking diet as an adjunct to neoadjuvant chemotherapy for breast cancer in the multicentre randomized phase 2 DIRECT trial. Nat. Commun..

[60] Bos JL (1989). ras oncogenes in human cancer: a review. Cancer Res..

[61] Di Tano M (2020). Synergistic effect of fasting-mimicking diet and vitamin C against KRAS mutated cancers. Nat. Commun..

[62] Phukan S, Babu VS, Kannoji A, Hariharan R, Balaji VN (2010). GSK3beta: role in therapeutic landscape and development of modulators. Br. J. Pharmacol..

[63] Cohen P, Goedert M (2004). GSK3 inhibitors: development and therapeutic potential. Nat. Rev. Drug Discov..

[64] Wu D, Li Y, Zhu KS, Wang H, Zhu WG (2018). Advances in cellular characterization of the Sirtuin isoform, SIRT7. Front. Endocrinol. (Lausanne).

[65] Tang, X. et al. HDAC8 cooperates with SMAD3/4 complex to suppress SIRT7 and promote cell survival and migration. *Nucleic Acids Res.*10.1093/nar/gkaa039 (2020). 10.1093/nar/gkaa039 PMC7102950 31970414

[66] Hubbi ME, Hu H, Kshitiz, Gilkes DM, Semenza GL (2013). Sirtuin-7 inhibits the activity of hypoxia-inducible factors. J. Biol. Chem..

[67] Dang CV, Kim JW, Gao P, Yustein J (2008). The interplay between MYC and HIF in cancer. Nat. Rev. Cancer.

[68] Vakhrusheva O (2008). Sirt7 increases stress resistance of cardiomyocytes and prevents apoptosis and inflammatory cardiomyopathy in mice. Circ. Res..

[69] Cai Z (2020). The Skp2 pathway: a critical target for cancer therapy. Semin Cancer Biol..

[70] Drobnjak M (2003). Altered expression of p27 and Skp2 proteins in prostate cancer of African-American patients. Clin. Cancer Res.

[71] Ben-Izhak O (2003). Inverse relationship between Skp2 ubiquitin ligase and the cyclin dependent kinase inhibitor p27Kip1 in prostate cancer. J. Urol..

[72] Zhao Y (2020). Sirtuin 7 promotes nonsmall cell lung cancer progression by facilitating G1/S phase and epithelialmesenchymal transition and activating AKT and ERK1/2 signaling. Oncol. Rep..

[73] Mo Y (2017). SIRT7 deacetylates DDB1 and suppresses the activity of the CRL4 E3 ligase complexes. FEBS J..

[74] Lee N (2014). Comparative interactomes of SIRT6 and SIRT7: implication of functional links to aging. Proteomics.

[75] Santos-Barriopedro I (2018). SIRT6-dependent cysteine monoubiquitination in the PRE-SET domain of Suv39h1 regulates the NF-kappaB pathway. Nat. Commun..

[76] Yamamura S (2020). Cardiomyocyte Sirt (Sirtuin) 7 ameliorates stress-induced cardiac hypertrophy by interacting with and deacetylating GATA4. Hypertension.

[77] White MC (2014). Age and cancer risk: a potentially modifiable relationship. Am. J. Prev. Med..

