# Supplementary material for: Combined intermittent fasting and ERK inhibition enhance the anti-tumor effects of chemotherapy via the GSK3β-SIRT7 axis
Source: Nat Commun. 2021 Aug 25;12:5058. doi: 10.1038/s41467-021-25274-3 (PMC8387475; doi:10.1038/s41467-021-25274-3)
Supplement: Supplementary file 1 — Supplementary Information [file 41467_2021_25274_MOESM1_ESM.pdf]

Supplementary information

This file contains Supplementary Figures 1 to 14, Supplementary Tables 1 to 2, and uncropped gels and blots.

Combined intermittent fasting and ERK inhibition enhance the anti-tumor effects of chemotherapy via the GSK3β-SIRT7 axis

Xiaolong Tang[1,2], Guo Li[3], Lei Shi[2], Fengting Su[1], Minxian Qian[1,2], Zuojun Liu[1,2], Yuan Meng[1], Shimin Sun[1], Ji Li[3], Baohua Liu[1,2,4*]

[1]Shenzhen Key Laboratory of Systemic Aging and Intervention (SAI), School of Basic Medical Sciences, Shenzhen University, Shenzhen 518055, China

[2]Guangdong Key Laboratory of Genome Stability and Human Disease Prevention, Marshall Laboratory of Biomedical Engineering, National Engineering Research Center for Biotechnology (Shenzhen), International Cancer Center, Shenzhen University, Shenzhen 518055, China

[3]Department of Dermatology, Xiangya Hospital, Central South University, Changsha, China

[4]Shenzhen Bay Laboratory, Shenzhen, China

*Correspondence should be addressed to Dr Baohua Liu (ppliew@szu.edu.cn).

Supplementary Fig. 1

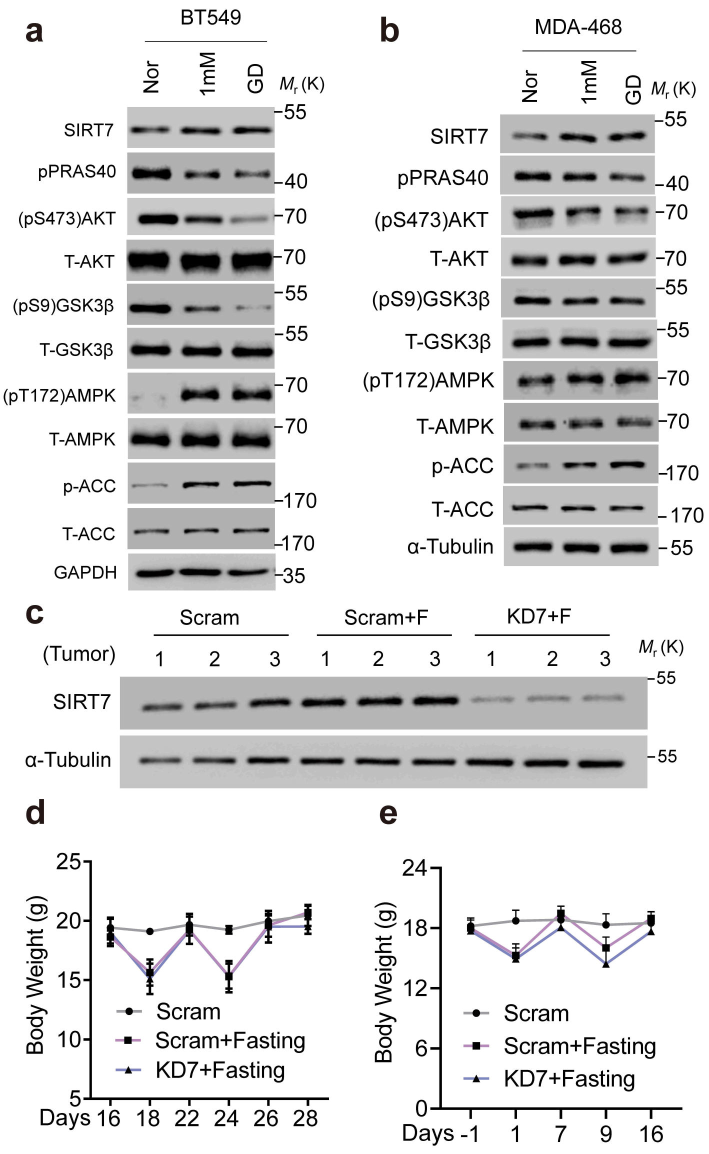

Supplementary Fig. 1 Analysis of glucose deprivation and intermittent fasting

a–b Immunoblots showing the indicated protein levels in BT-549 and MDA-MB-468 breast cancer cells treated with various glucose concentrations, Representative results were obtained from at least three independent experiments. **c** Immunoblotting analysis of SIRT7 protein levels in tumor xenografts isolated from Fig. 1g, n = 3 mice per group. **d–e** Body weight was measured in the experimental groups shown in Figure 1g (d) and h (e); data represent means ± SEM (d and e). Source data are provided as a Source Data file.

Supplementary Fig. 2

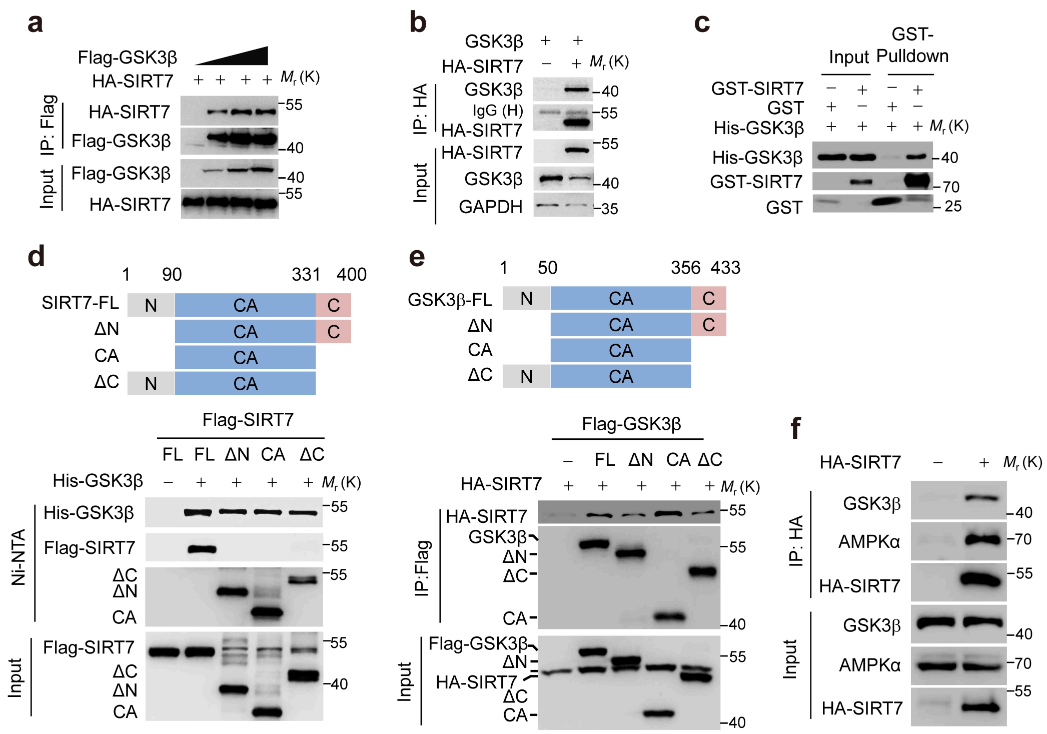

Supplementary Fig. 2 GSK3β interacts with SIRT7

a–b Co-immunoprecipitation (Co-IP) and immunoblotting analysis of the interaction between ectopic HA-SIRT7 and Flag-GSK3β in HEK293 cells transfected with the indicated plasmids. **c** Immunoblotting analysis of the GST-pulldown eluate derived from incubation of recombinant GST-SIRT7 and His-GSK3β *in vitro*. **d–e** Immunoblots showing the interaction of truncated SIRT7 with GSK3β (d) or truncated GSK3β with SIRT7 (e) in HEK293 cells. **f** Immunoblotting analysis of the anti-HA-SIRT7 immunoprecipitates from HEK293 cells overexpressing ectopic HA-SIRT7. Representative results were obtained from at least three independent experiments.

Supplementary Fig. 3

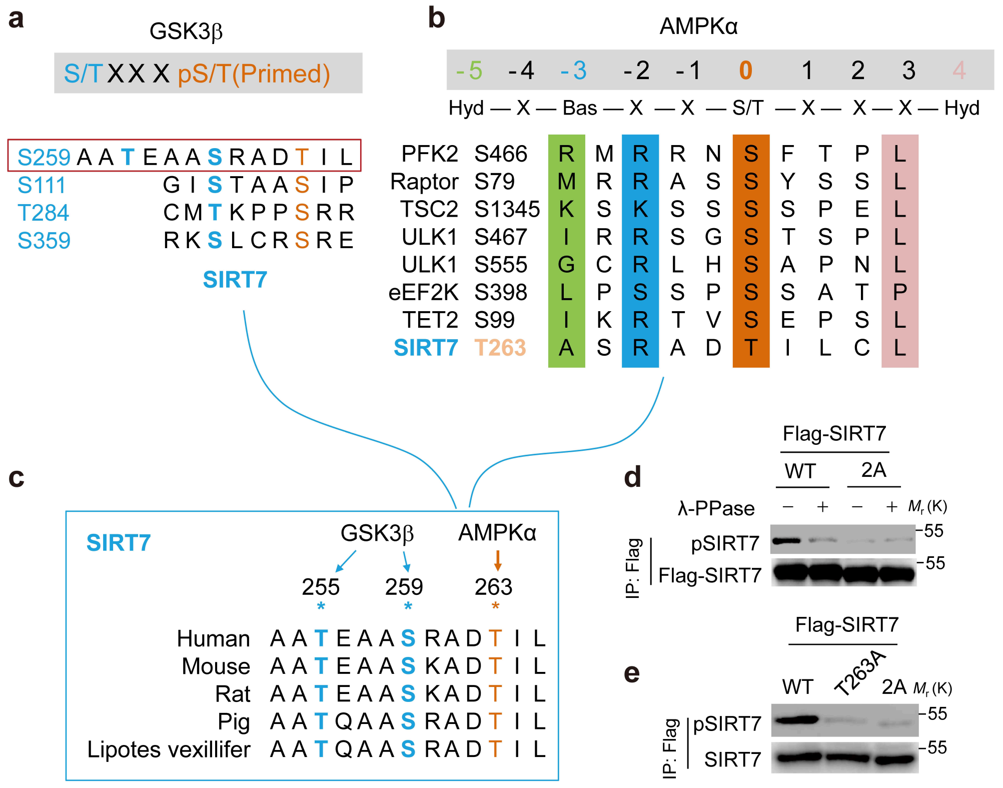

Supplementary Fig. 3 GSK3β phosphorylates SIRT7 at T255/S259 after priming phosphorylation at T263 by AMPK

a Peptide sequence showing potential residues (red) recognized by GSK3β. **b** Conserved motif recognized by AMPK, highlighting SIRT7 T263 as a potential target. **c** Conserved SIRT7 motifs targeted by GSK3β and AMPK in different species. **d–e** Lambda protein phosphatase (λ-PPase) mediated *in vitro* dephosphorylation assay (d) and immunoblotting analysis of cells expressing the indicated SIRT7 mutants (e), showing the specificity of the anti-p(T255/S259)SIRT7 (p-SIRT7) antibody.

Supplementary Fig. 4

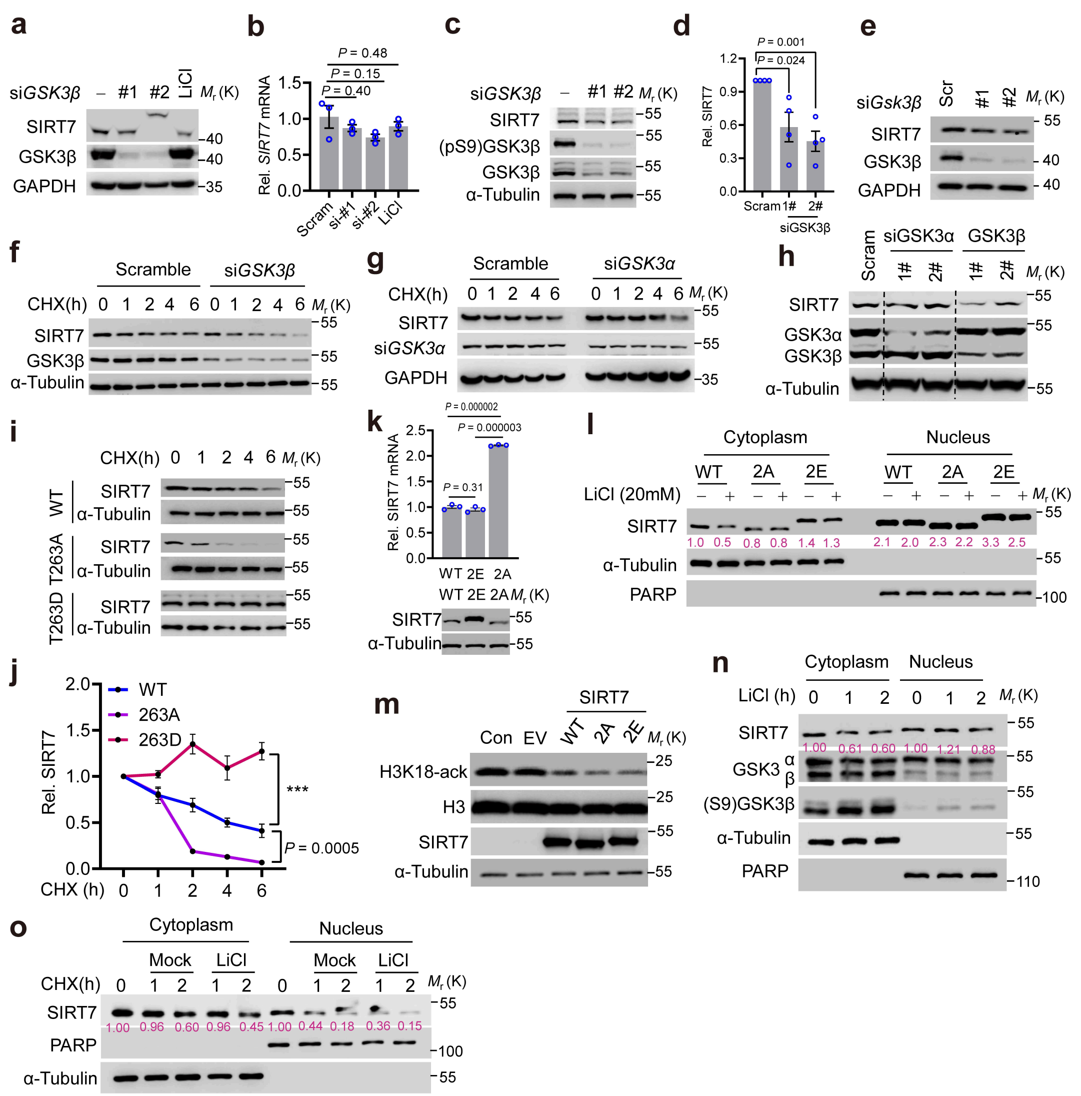

Supplementary Fig. 4 GSK3β plays critical roles in SIRT7 stabilization

a–b Immunoblotting and quantitative PCR analysis (n = 3 biologically independent samples) of SIRT7 levels in breast cancer MCF-7 cells treated with *GSK3β* siRNA or LiCl (10 mM). **c** Immunoblotting analysis (c) of SIRT7 protein expression in BT549 breast cancer cells with or without *GSK3β* knockdown and the related quantification (d) was based on four biologically independent samples. **e** Immunoblots showing the levels of SIRT7 in 4T1 cells treated with *GSK3β* siRNAs or not. **f** Immunoblotting analysis of SIRT7 stability determined by CHX (50 μg/ml) chase assay in HeLa cells treated with or without *GSK3β* siRNA. **g** Immunoblotting analysis of SIRT7 protein stability determined by CHX (50 μg/ml) chase assay in HeLa cells treated with *GSK3α* siRNA or not. **h** Immunoblots showing SIRT7 levels in HeLa cells treated

with GSK3α or GSK3β siRNA. **i–j** Immunoblotting analysis (i) of SIRT7 WT, 263A and T263D protein stability determined by CHX (50 μg/ml) chase assay in HEK293 cells and (j) showing the related quantification from three biologically independent samples. **k** SIRT7 expression was evaluated by quantitative PCR (upper, n = 3 samples) and immunoblotting analysis (below) in MDA-231 breast cancer cells. **l** Immunoblots showing the cytoplasmic or nuclear SIRT7 levels in cells exposed to LiCl or not. **m** Immunoblotting analysis of acetylated H3K18 levels in cells expressing indicated SIRT7 mutants. **n** Immunoblots showing the endogenous SIRT7 expression in the presence of LiCl (10 mM) for the indicated time. **o** Immunoblotting analysis of the stability of cytoplasmic or nuclear SIRT7 using CHX chase assay in cells with or without LiCl treatment (10 mM). Data represent means ± SEM (b, d, j and k). *P*-values were determined by two-tailed Student's *t*-test (b, d and k) or two-way ANOVA analysis (j), *** P = 0.0000007. Representative results were obtained from at least three independent experiments. Source data are provided as a Source Data file.

Supplementary Fig. 5

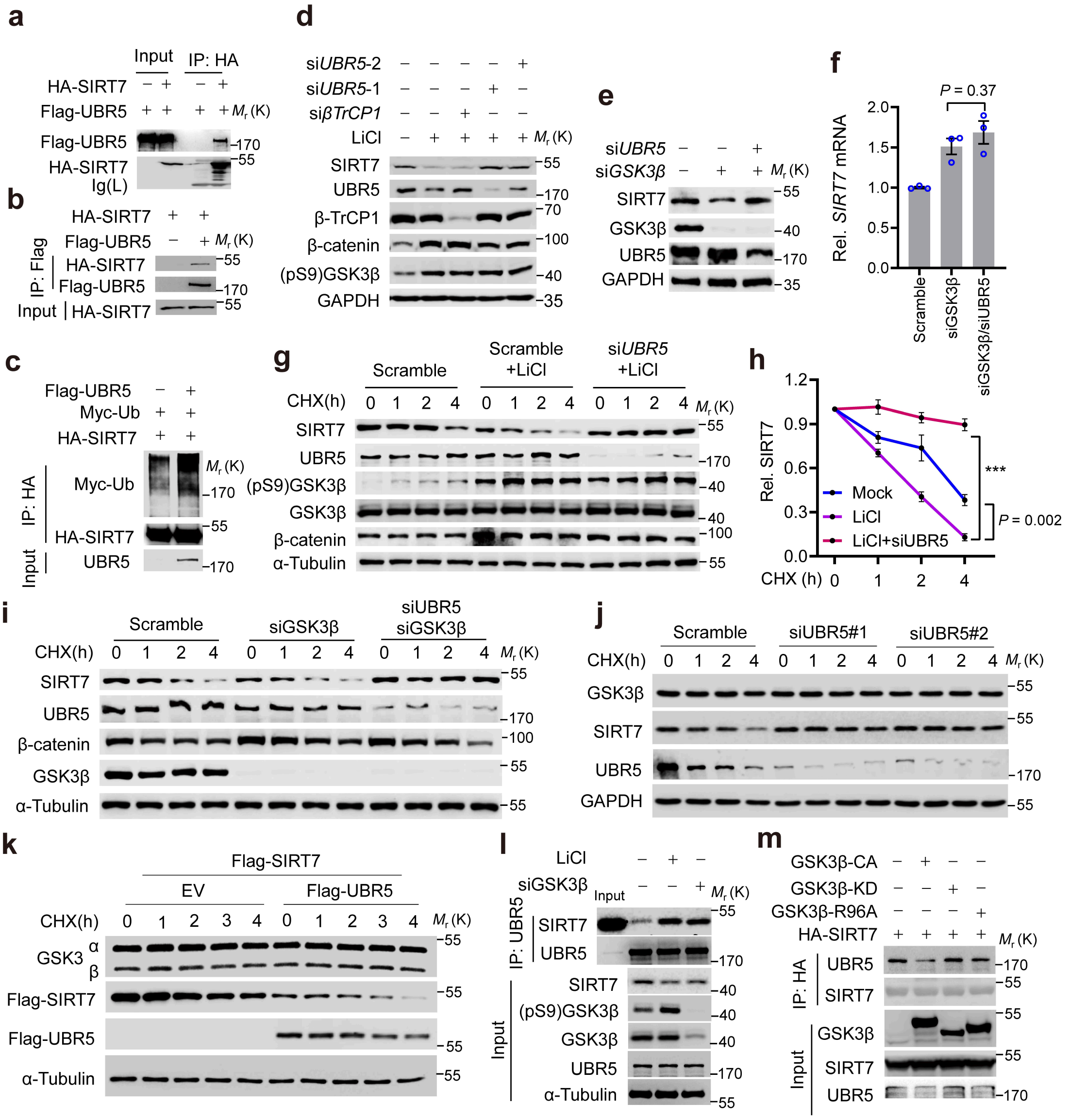

Supplementary Fig. 5 UBR5 contributes to SIRT7 degradation

a–b Immunoblotting of immunoprecipitates showing the interaction between ectopic HA-SIRT7 and FLAG-UBR5 in HEK293 cells. **c** Ubiquitinated SIRT7 levels were analyzed in HEK293 cells with or without overexpression of UBR5. **d** Immunoblots showing SIRT7 levels in HeLa cells treated with siRNA or LiCl (10 mM). **e** Immunoblots showing SIRT7 levels in HeLa cells treated with *GSK3β* and/or *UBR5* siRNA. **f** Quantitative PCR analysis (n = 3 biologically independent samples) of *SIRT7* mRNA levels (related to e). **g–i** SIRT7 protein stability determined by CHX (50 µg/ml) chase assay of HeLa cells transfected with Scramble or *UBR5* siRNAs (si*UBR5*) and treated with LiCl (10 mM) (g–h) or GSK3β siRNA (i) as indicated; quantification (h) was derived from three independent experiments. **j–k** Evaluation of

GSK3β protein stability by CHX (50 μg/ml) chase assays in MDA-231 cells with *UBR5* knockdown (j) or overexpression (k). **l** Immunoblotting of immunoprecipitates showing binding of endogenous SIRT7 and UBR5 in HEK293 cells following GSK3β knockdown or treatment with LiCl (10 mM). **m** Immunoblotting analysis of anti-HA-SIRT7 immunoprecipitation eluate derived from HEK293 cells transfected with the plasmids, GSK3β-CA (constitutively active), GSK3β-KD (kinase dead, GSK3β-K85M/K86I) or GSK3β-R96A (specific recognition of the non-priming phosphorylation substrates) as indicated. Data represent means ± SEM (f and h). *P*-values were determined by two-tailed Student's *t*-test (f) or two-way ANOVA analysis (h), *** $P = 0.00000000003$. Representative results were obtained from at least three independent experiments. Source data are provided as a Source Data file.

Supplementary Fig. 6

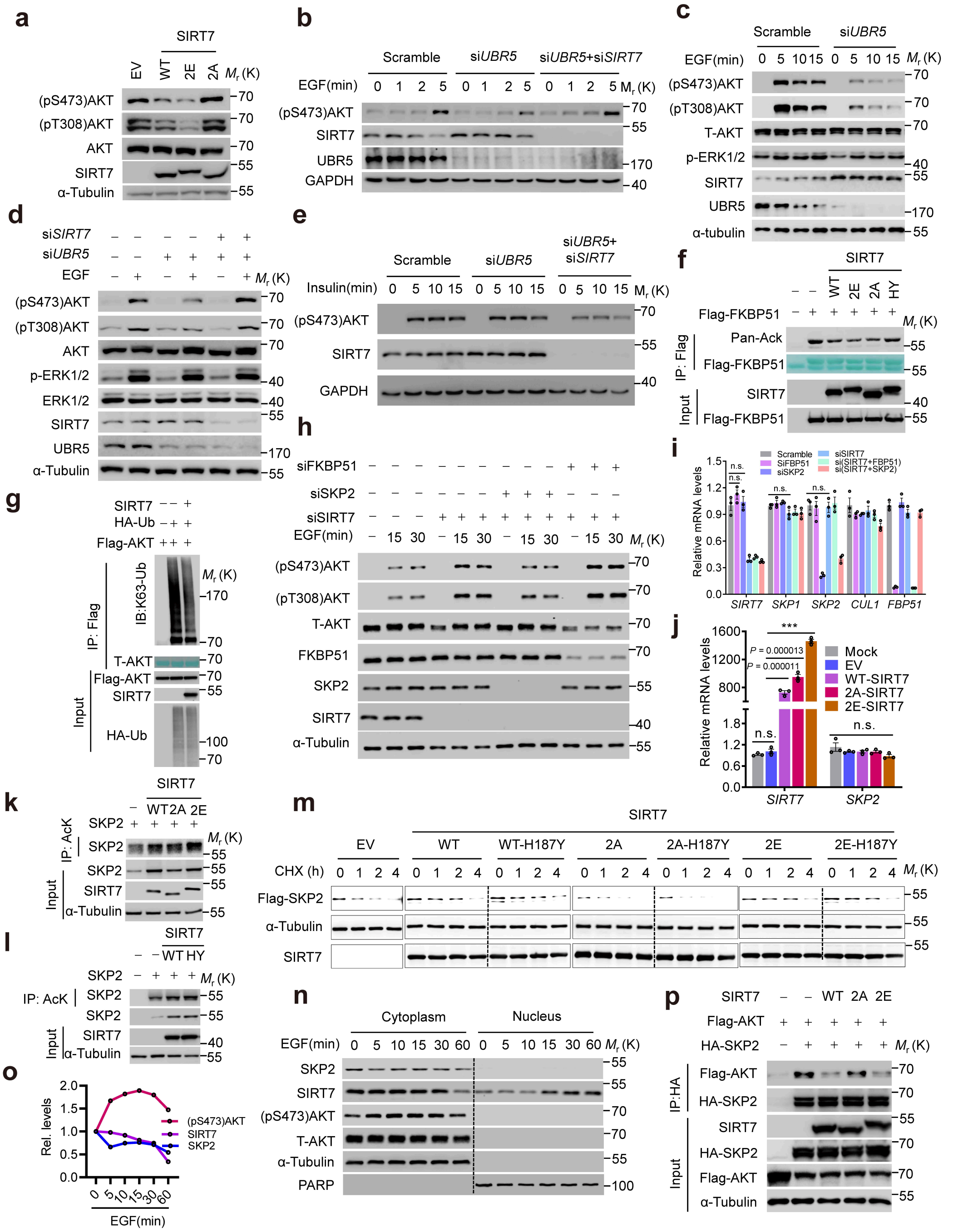

Supplementary Fig. 6 SIRT7 suppresses EGF-driven AKT activation

a Immunoblots showing AKT phosphorylation levels in HEK293 cells transfected with empty vector (EV), SIRT7-WT, SIRT7-2E and SIRT7-2A. **b–d** AKT activation assessed in MDA-231 (b) and BT-549 (c and d) breast cancer cells as indicated (EGF, 5 ng/ml). **e** AKT activation was assessed in BT-549 breast cancer cells with UBR5 or SIRT7 knockdown in the presence of insulin (10 μg/ml). **f** FKBP51 acetylation was detected by anti-pan-ack antibody in the

anti-FLAG-FKBP51 immunoprecipitates of from lysates of HEK 293 cells expressing the indicated proteins. **g** Immunoblotting analysis of ubiquitinated AKT by probing with anti-K63 ubiquitin antibody. **h** Immunoblots detecting AKT activation in MDA-231 breast cancer cells with the indicated *SKP2*, *FKBP51* or *SIRT7* knockdown in the presence or absence of EGF (5 ng/ml). **i–j** Quantitative PCR showing gene expression levels in MDA-231 cells treated as indicated, n = 3 biologically independent samples for each group. **k–l** Immunoblotting analysis of acetylated SKP2 in anti-ACK immunoprecipitates of HEK 293 cells transfected as indicated by probing with an anti-SKP2 antibody. **m** Analysis of SKP2 protein stability by CHX chase assays in HEK293 cells transfected with the indicated plasmids. **n–o** Immunoblots (n) showing protein expression levels in the lysates of MDA-231 breast cancer cells exposed to EGF (5 ng/ml) at the indicated time-points. The curve (o) represents the related quantification. **p** Immunoblots showing the binding of FLAG-AKT and HA-SKP2 in HEK293 cells overexpressing SIRT7-WT, SIRT7-2E and SIRT7-2A. Note: total Flag-FKBP51 and AKT in immunoprecipitates of (f and g) are shown as membrane stained with Fast Green solution. Data represent means ± SEM (i and j). *P*-values were determined by two-tailed Student's *t*-test (i and j), *** *P* = 0.000001, n.s., no significance. Representative results were obtained from at least three independent experiments. Source data are provided as a Source Data file.

Supplementary Fig. 7

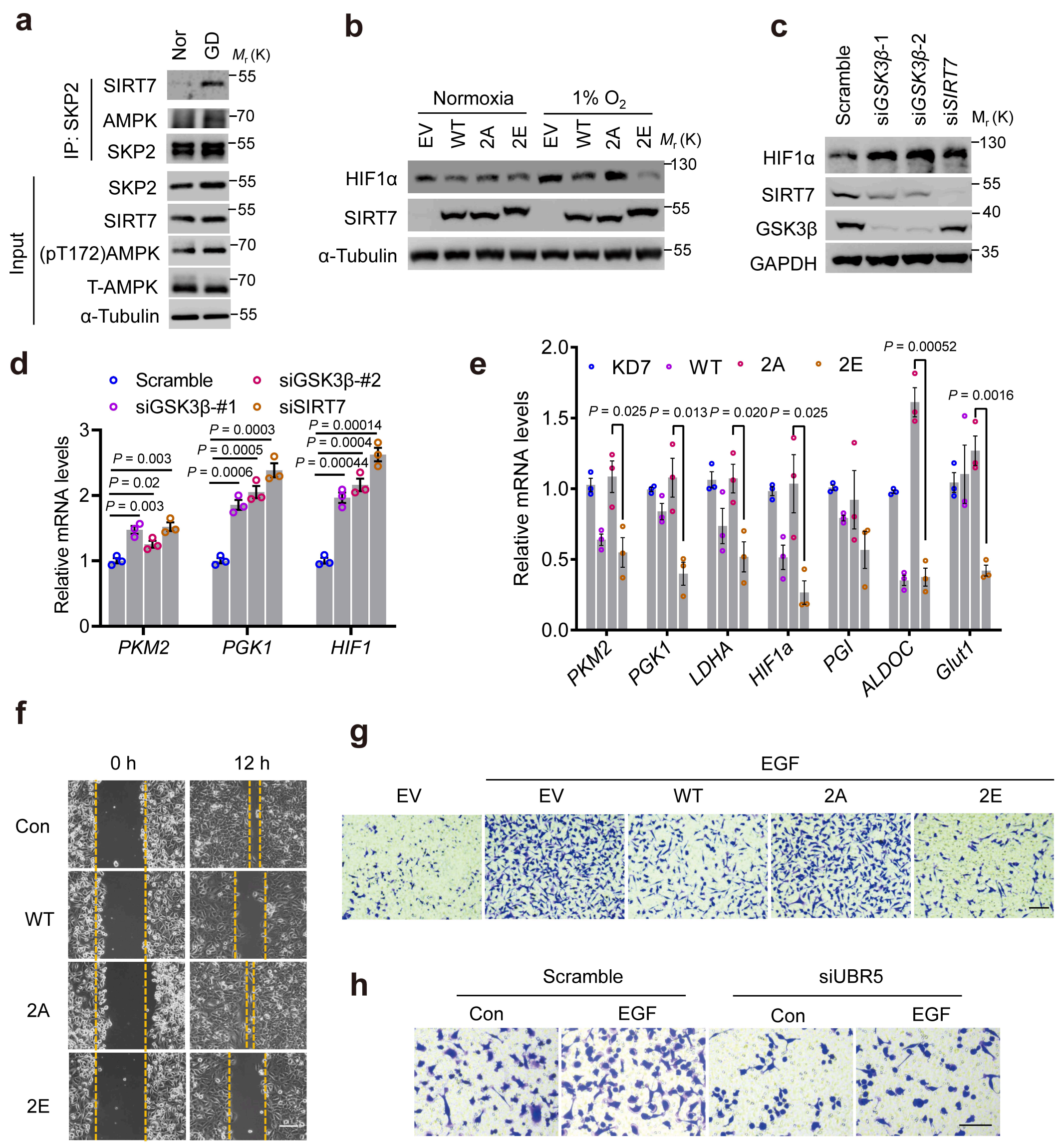

Supplementary Fig. 7 SIRT7 inhibits SKP2-mediated AKT activation

a Immunoblotting analysis of protein levels in the anti-SKP2 immunoprecipitates derived from lysates of MDA-231 breast cancer cells subjected to GD for 30 min. **b** Immunoblot showing HIF1α levels in MDA-231 breast cancer cells expressing empty vector (EV), SIRT7-WT, SIRT7-2E and SIRT7-2A cultured under normoxia or hypoxia (1% O_2). **c** Immunoblots showing HIF1α levels in breast cancer MDA-231 cells with *GSK3β* or *SIRT7* knockdown and cultured under hypoxia. **d** Quantitative PCR showing mRNA levels of glycolytic genes in cells related to (c), n= 3 biologically independent samples. **e** Quantitative PCR analysis of glycolytic genes in breast cancer MDA-231 cells expressing SIRT7 WT or the indicated mutants under hypoxia

(1% O_2), n= 3 biologically independent samples. **f** Would-healing assay showing the collective migration ability of H1975 lung cancer cells expressing empty vector (EV), SIRT7-WT, SIRT7-2E and SIRT7-2A. **g** Transwell migration assay showing the migratory ability of MDA-231 breast cancer cells expressing empty vector (EV), SIRT7-WT, SIRT7-2E and SIRT7-2A in response to EGF (10 ng/ml) treatment. Scale bar, 200 μm. **h** Transwell migration assay of the motility of MDA-231 breast cancer cells transfected with si*UBR5* or scramble siRNA in response to EGF (10 ng/ml) treatment. Scale bar, 100 μm. Data represent means ± SEM (d and e). *P*-values were determined by two-tailed Student's *t*-test (d and e). Representative results were obtained from at least three independent experiments. Source data are provided as a Source Data file.

Supplementary Fig. 8

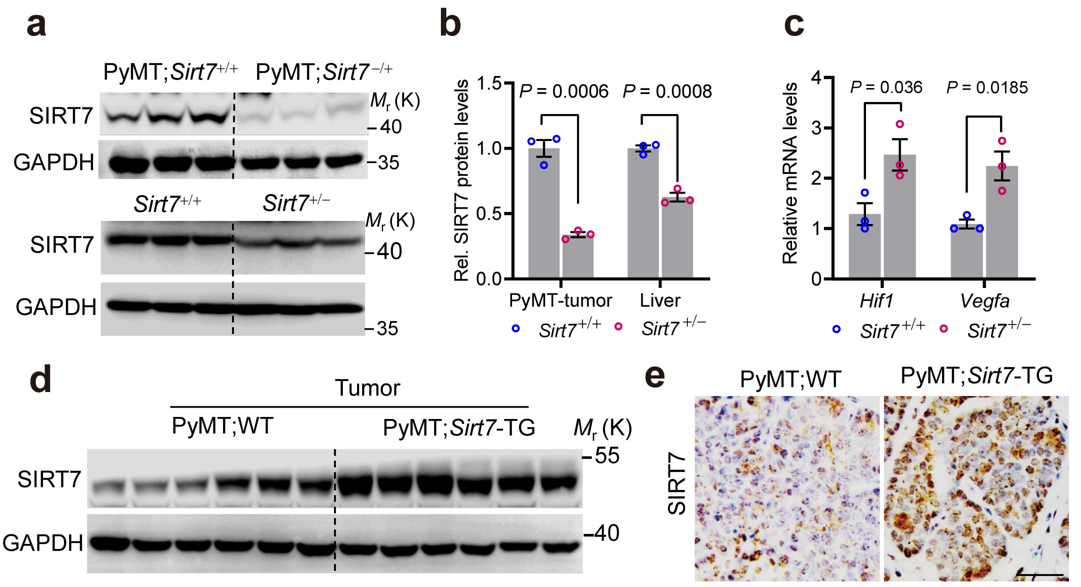

Supplementary Fig. 8 *Sirt7* insufficiency accelerates tumor progression

a–b Immunoblots showing Sirt7 expression in tumors of PyMT;*Sirt7*$^{+/+}$ and PyMT;*Sirt7*$^{+/-}$ mice (a, upper) or livers of normal *Sirt7*$^{+/+}$ and *Sirt7*$^{+/-}$ mice (a, lower). The related quantification is shown in b (n = 3 mice per genotype). **c** Quantitative PCR analysis (n = 3 mice per group) of *Hif1α* and *Vegfa* mRNA levels in mammary tumors isolated from mice of Figure 6b. **d** Immunoblots showing Sirt7 protein levels in mammary tumors of PyMT;WT and PyMT;*Sirt7*-TG mice (n = 6 mice per genotype). **e** Representative images of IHC staining showing SIRT7 expression in tumors of PyMT;WT and PyMT;*Sirt7*-TG mice, analyzed from n = 3 mice for each genotype. Scale bar, 50 μm. Data represent means ± SEM (b and c). *P*-values were determined by two-tailed Student's *t*-test (d and e). Source data are provided as a Source Data file.

Supplementary Fig. 9

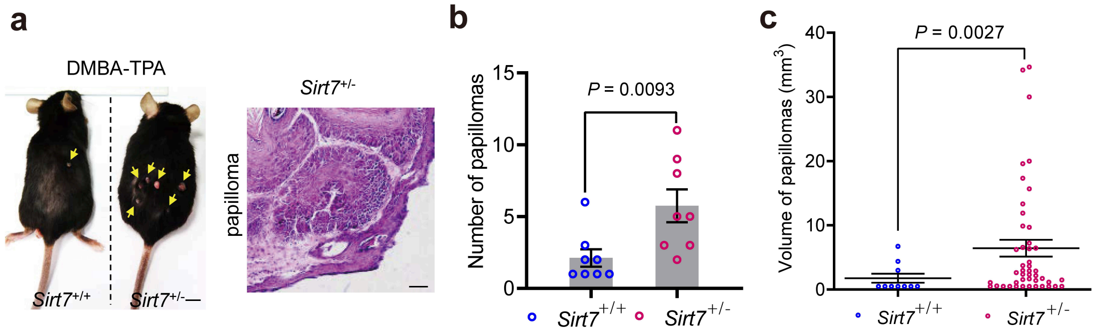

Supplementary Fig. 9 *Sirt7* insufficiency promotes skin carcinogenesis

a Representative images showing skin carcinogenesis induced in C57/BL6 mice (n = 8 mice per genotype) using the two-stage DMBA-TPA model. Both *Sirt7*$^{+/+}$ and *Sirt7*$^{+/-}$ mice were treated with DMBA followed by exposure of the dorsal skin to TPA for 35 weeks. Yellow arrows indicate papillomas. **b–c** Statistical analysis showing the average papilloma number (b) and volume (c, total papillomas) (n = 8 mice per genotype). Data represent means ± SEM (b and c). *P*-values were determined by two-tailed Student's *t*-test (d and e). Source data are provided as a Source Data file.

Supplementary Fig. 10

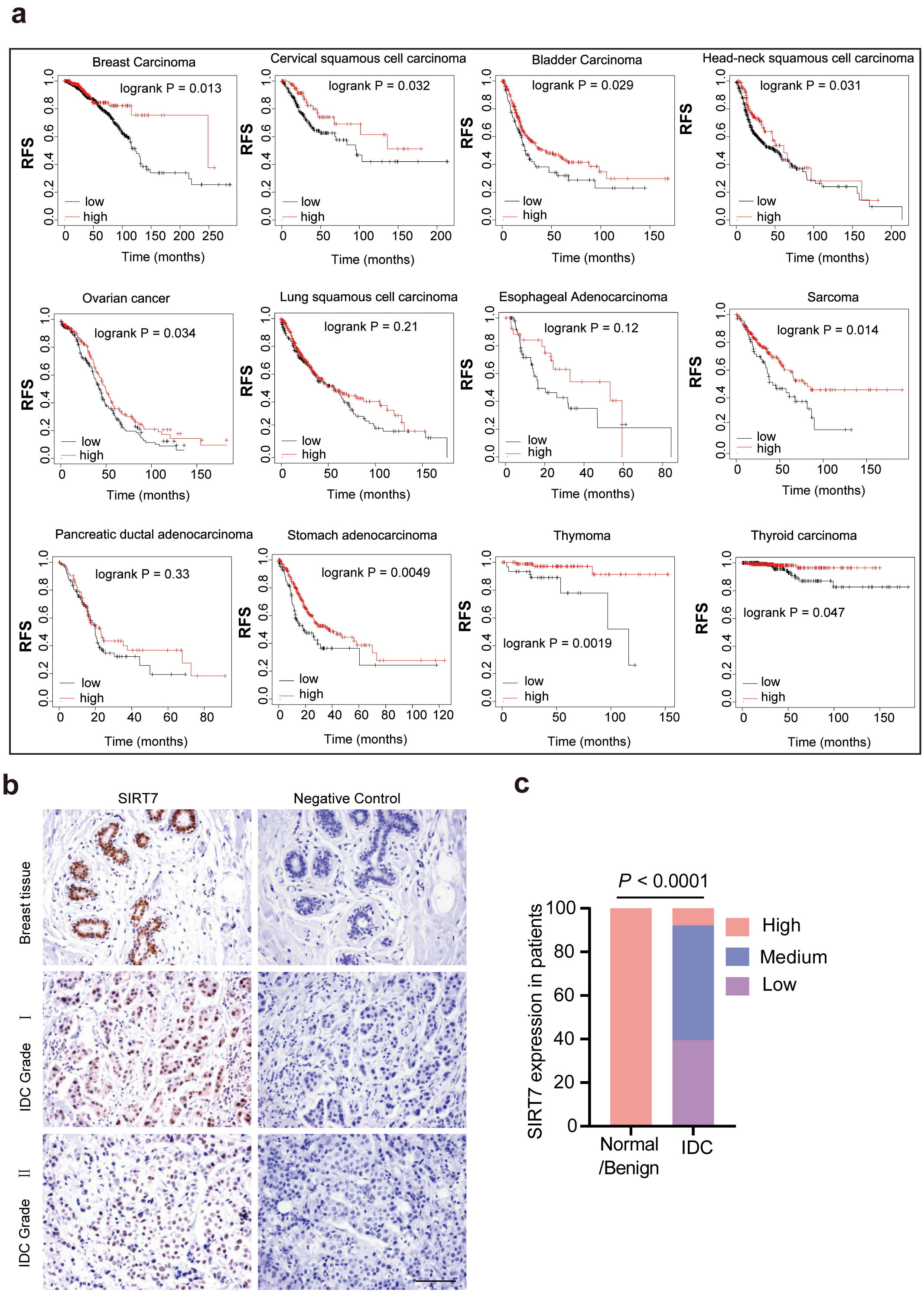

Supplementary Fig. 10 Higher SIRT7 level is associated with a better prognosis in cancer patients

a Analysis of recurrence free survival (RFS) in patients based on *SIRT7* expression. **b** Representative images showing IHC staining of SIRT7 expression in samples of normal or

precancerous breast tissues (n = 9) and malignant invasive ductal breast cancers (IDCs, n = 38), scale bar, 100 μm. **c** The association of SIRT7 expression and breast cancer progression was analyzed according to scores of the IHC staining from (b). *P*-values were determined by *Chi*-squared test, $P = 0.0000001$ for (c).

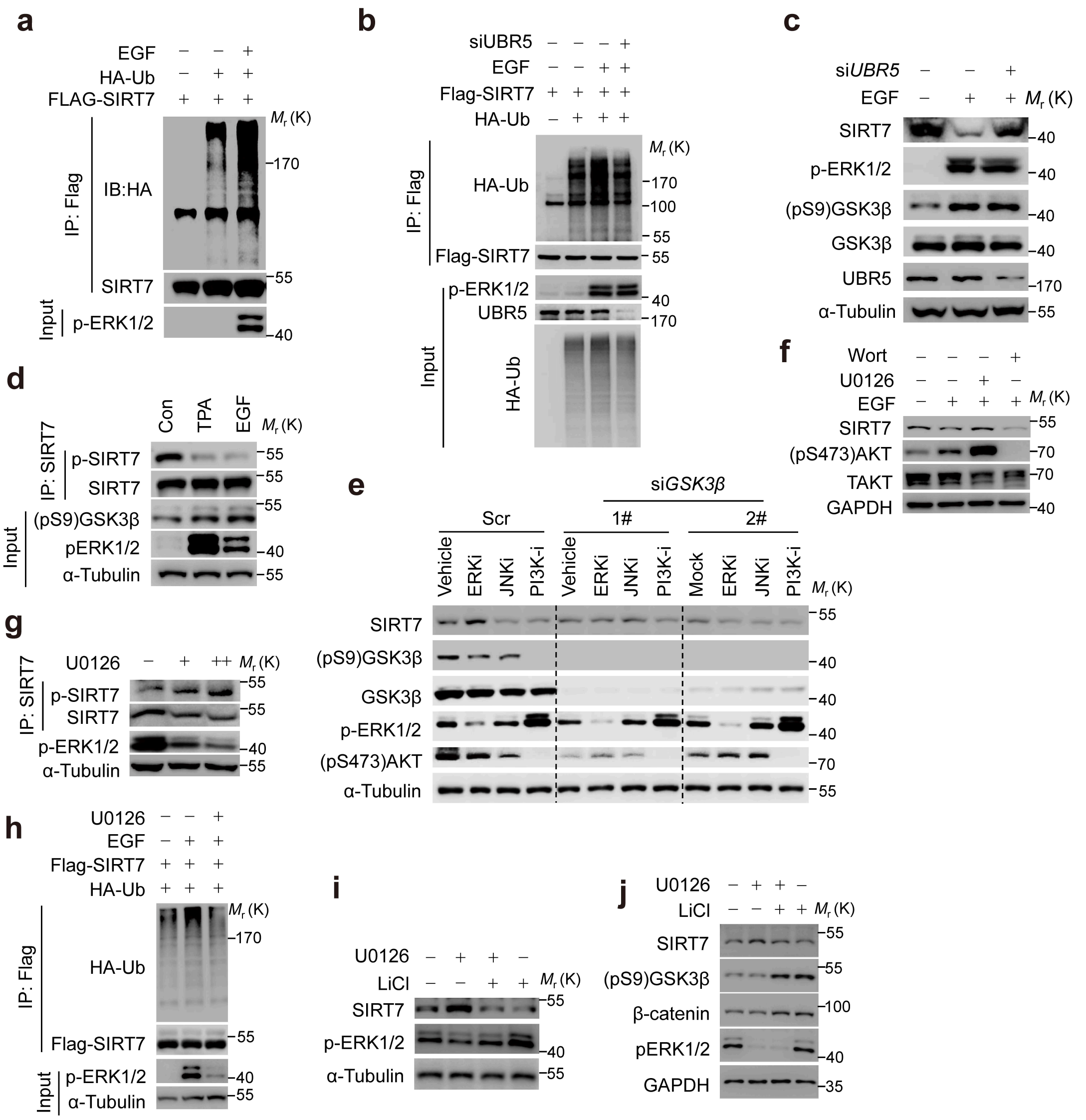

Supplementary Fig. 11 EGF regulates the GSK3β-SIRT7 axis

a–b Ubiquitinated SIRT7 levels were evaluated in HEK293 cells transfected with the indicated constructs and with or without EGF (10 ng/ml) treatment. **c** SIRT7 levels were detected in HEK293 cells with or without *UBR5* knockdown or EGF incubation (10 ng/ml). **d** Immunoblots showing p-SIRT7 levels in HEK293 cells treated as indicated, EGF (10 ng/ml), 15 min; TPA (1 μM), 10 min. **e** Immunoblotting analysis of lysates of cells treated with the indicated inhibitors or siRNA; ERKi, MEK-ERK inhibitor U0126 (2 μM); JNKi, JNK1-3 inhibitor SP600125 (5 μM); PI3Ki, PI3K inhibitor wortmannin (1 μM). **f** Immunoblotting analysis of SIRT7 levels in MDA-231 breast cancer cells treated as indicated, EGF (10 ng/ml), U0126 (2 μM) and

wortmannin (5 μM). **g** p-SIRT7 levels were assessed in MDA-231 cells in the presence of U0126 (+, 1 μM; ++, 2 μM). **h** Immunoblot showing ubiquitinated SIRT7 levels in HEK293 cells treated and transfected as indicated transfections, U0126, 2 μM; EGF, 10 ng/ml. **i–j** SIRT7 levels were detected in cells incubated with U0126 (2 μM) and LiCl (10 mM). Representative results were obtained from at least three independent experiments.

Supplementary Fig. 12

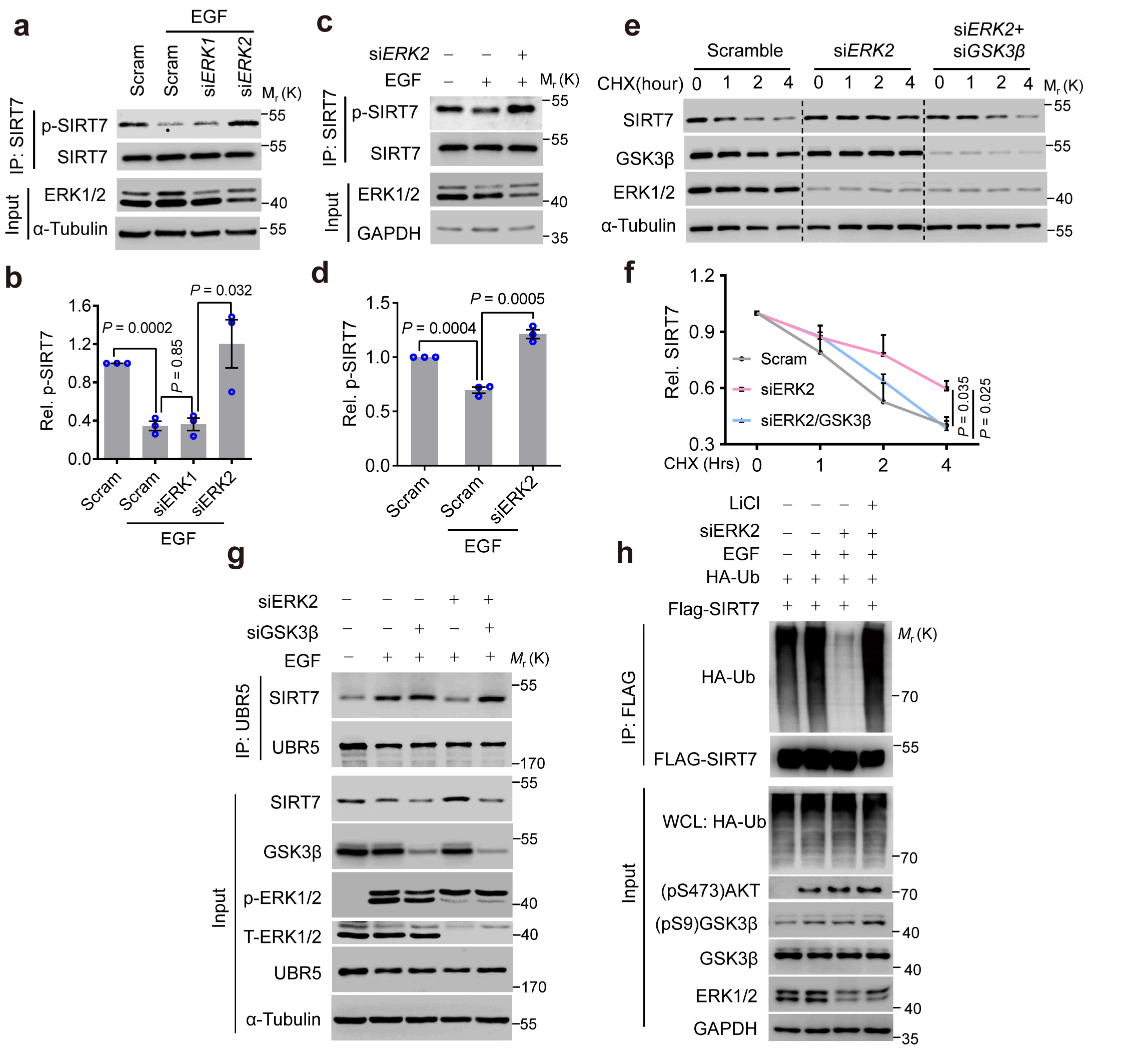

Supplementary Fig. 12 EGF modulates the GSK3β-SIRT7 axis via ERK2

a–b Immunoblotting analysis (a) of p-SIRT7 levels in MDA-231 breast cancer cells treated with or without EGF (5 ng/ml) and/or the indicated siRNA; bar chart (b) showing the related quantification from three biologically independent samples. **c–d** Immunoblotting analysis (c) of p-SIRT7 levels in *ERK2* knocked down MDA-231 cells treated with or without EGF (5 ng/ml); bar chart (d) showing the related quantification, n = 3 biologically independent samples. **e–f** Immunoblotting (e) analysis of SIRT7 protein stability in A549 cells transfected with the indicated siRNA, determined by CHX (50 μg/ml) chase assay; Curve (f) showing the related quantification from three biologically independent samples. **g** The binding of SIRT7 to UBR5 assessed in MDA-231 cells treated as indicated. **h** Ubiquitinated SIRT7 levels assessed in

HEK293 cells transfected with the indicated siRNA and/or plasmids; U0126 (2 μM) and EGF (10 ng/ml). Data represent means ± SEM (b, d and f). *P*-values were determined by two-tailed Student's *t*-test (b and d) or two-way ANOVA analysis (f). Representative results were observed from at least three independent experiments. Source data are provided as a Source Data file.

Supplementary Fig. 13

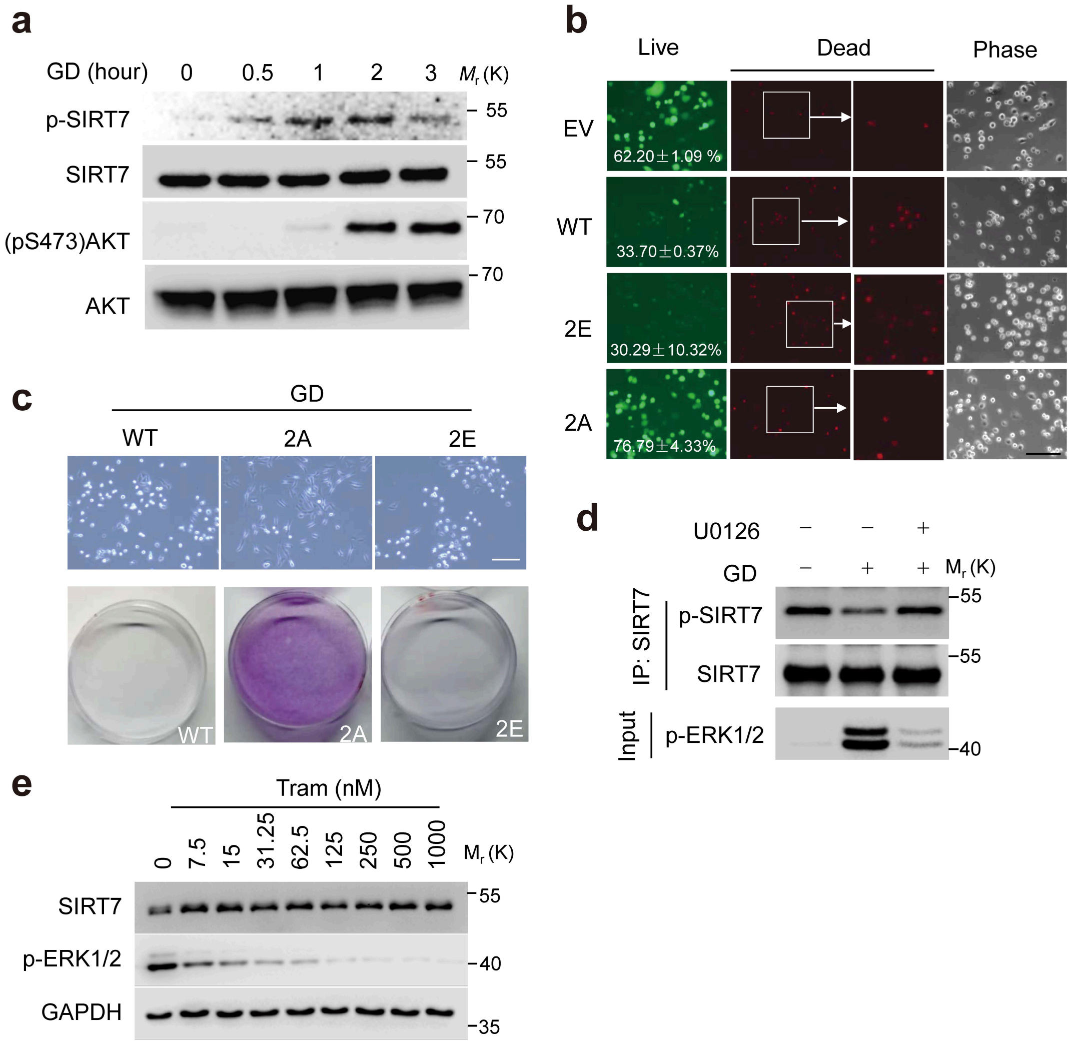

Supplementary Fig. 13 Long-term glucose deprivation inhibits GSK3β-SIRT7 signaling to promote cell survival

a Immunoblotting analysis of p-SIRT7 levels in MDA-231 breast cancer cells cultured under glucose deprivation (GD) for the indicated times. **b** Cell viability analysis of MDA-231 cells expressing empty vector (EV) or the indicated SIRT7s and cultured under glucose deprivation. Green and red fluorescence indicate dead and live cells, respectively. Scale bar, 200 μm. **c** Representative images (upper) showing the morphology of MDA-231 cells cultured under glucose deprivation for 12 h. The remaining cells were then cultured in nutrient rich medium for 7 days and stained with crystal violet. Scale bar, 200 μm. **d** Detection of p-SIRT7 in MDA-231 breast cancer cells cultured under glucose deprivation with or without U0126 (2 μM). **e** Immunoblots showing SIRT7 levels in 4T1 cells incubated with the indicated doses of

trametinib (Tram). Representative results were observed from at least three independent experiments.

Supplementary Fig. 14

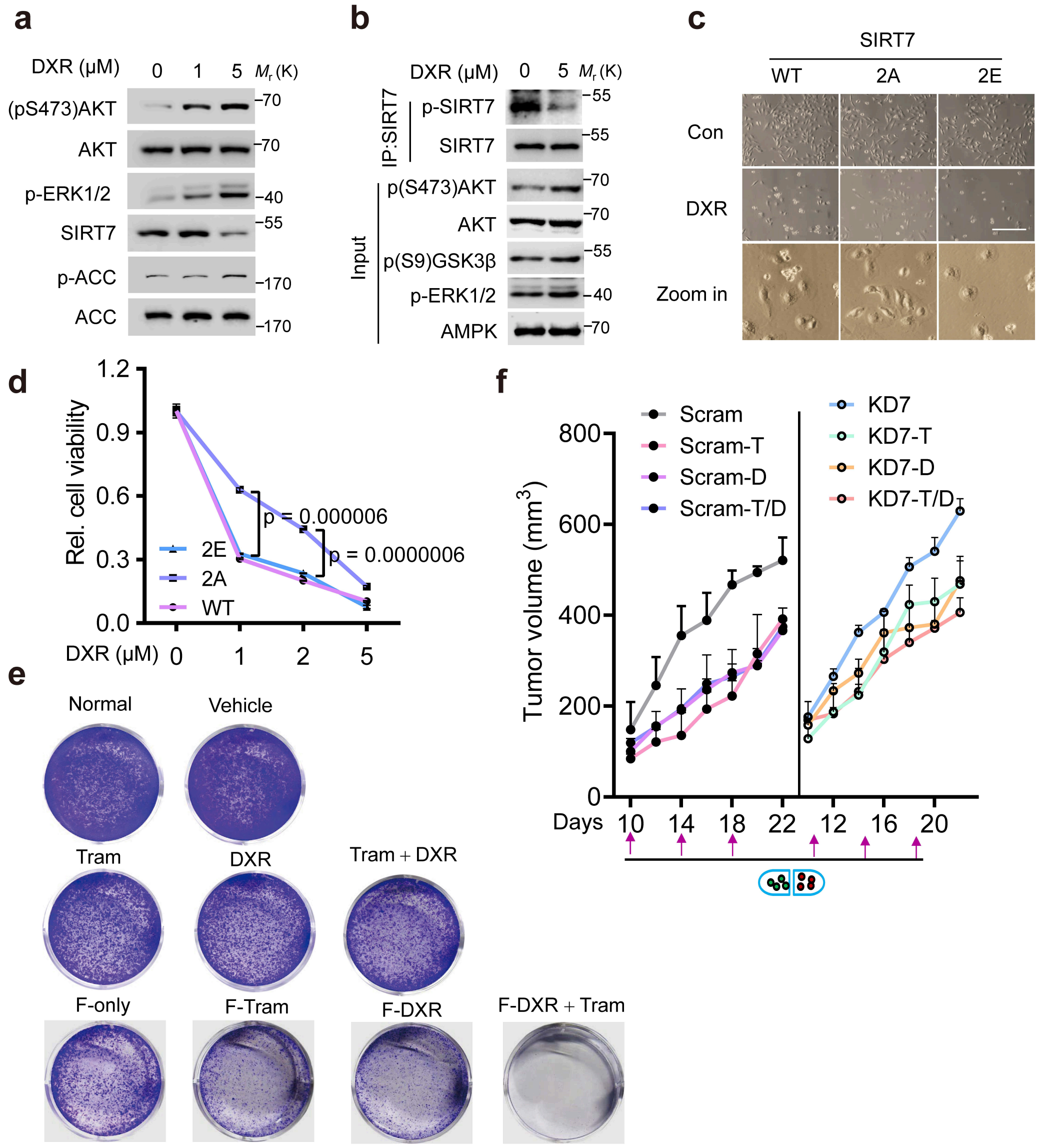

Supplementary Fig. 14 GSK3-SIRT7 axis underlies chemotherapy resistance

a–b Immunoblots showing levels of the indicated proteins in MDA-231 cells exposed to doxorubicin (DXR) for 24 h. **c–d** Representative images (c) showing the morphology of MDA-231 breast cancer cells expressing the indicated SIRT7s with or without DXR treatment for 48 h. Scale bar, 200 μm. The relative cell viability was measured using the CCK8 assay as shown in (d, n = 3 biologically independent samples). **e** Representative images showing the growth of 4T1 cells treated as indicated, F, fasting; Tram, trametinib; DXR, doxorubincin. **f** Growth curves of tumors generated from Scram or *Sirt7* KD 4T1 cells treated as indicated, n = 3 mice per group; arrows indicate drug application. Representative results were observed from at least three independent experiments.

Data represent means ± SEM (d and f). *P*-values were determined by two-way ANOVA analysis (d). Source data are provided as a Source Data file.

Supplementary Table 1 Antibodies used in this study		
Antibody	Source	Dilutions (WB, Western blotting; IP, immunoprecipitation)
SIRT7	Santa Cruz (sc-365344)	WB/IP (1:3,000/1:100)
SIRT7	EMD Millipore (ABE103)	IHC (1:50)
(pSer9)-GSK3β	CST (#9322)	WB (1:1,000)
GSK3β	CST (#12456)	WB (1:1,000)
GSK3α/β	CST (#5676)	WB (1:100)
(pSer473)-AKT	CST (#4060S)	WB (1:1,000)
(pThr308)-AKT	CST (#13038)	WB (1:1,000)
AKT	CST (#4691)	WB (1:1,000)
(pThr202/Tyr204) ERK1/2	CST(#4370S)	WB (1:1,000)
ERK1/2	CST (#4695S)	WB (1:1,000)
(pThr172)-AMPK	CST (#50081)	WB (1:1,000)
AMPKα1/2	Abcam (ab80039)	WB (1:2,000)
(pSer79)-ACC	CST(#3661)	WB (1:1,000)
ACC	CST (#3676)	WB (1:1,000)
Phosphoserine/threonine	ECM biosciences (PM3801)	WB (1:1,000)
His-tag	Proteintech (66005-1)	WB/IP (1:5,000/1:500)
HA-tag	Sigma–Aldrich (H3663)	WB (1:5,000)
FLAG-tag	Sigma–Aldrich (F3165)	WB(1:5,000)
α-Tubulin	Beyotime (AT819)	WB (1:5,000)
GAPDH	Beyotime (AG019)	WB (1:5,000)
Anti-mouse IgG	Jackson (11-035-003)	WB (1:10,000)
Anti-rabbit IgG	Jackson (15-035-003)	WB (1:10,000)
UBR5	CST (#65344)	WB (1:1,000)
EDD1	Abcam (ab70311)	IP (1:500)
HIF1α	Novus Biologicals (NB100-105)	WB (1:500)
SKP2	CST (#2652)	WB (1:1,000)
SKP1	CST (#12248)	WB (1:1,000)
CUL1	CST (#4995)	WB (1:1,000)
FKBP51	CST (#12210)	WB (1:1,000)
K63-linkage Specific Polyubiquitin	CST (#12930)	WB (1:500)
K48-linkage Specific Polyubiquitin	CST (#12805)	WB (1:500)

Supplementary Table 2 Oligonucleotides used in this study		
**Targets**	**Sequence (5'-3')**	**Purpose**
h*SIRT7*	CUCACCGUAUUUCUACUACUA	siRNA
h*GSK3β*-2	GUAUUGCAGGACAAGAGAU	siRNA
h*GSK3β*-1	GGACAAGAGAUUUAAGAAU	siRNA
h*UBR5*-1	AACUUAGAUCUCCUGAA	siRNA
h*UBR5*-2	AGACAAAUCUCGGACUUGA	siRNA
h*MEK1*	GUGAAUAAAUGCUUAAUAA	siRNA
h*MEK2*	GCAUUUGCAUGGAACACAU	siRNA
h*ERK1*	CGUCUAAUAUAUAA-AUAUA	siRNA
h*ERK2*	GUUCGAGUAGCUAUCAAGA	siRNA
h*GSK3α*-1	GUUCAAGUUCCCUCAGAUUAA	siRNA
h*GSK3α*-2	ACUAGAGGGCAGAGGUAAAU	siRNA
m*Vegfa*-F	GGAGAGCAGAAGTCCCATGA	qRT-PCR
m*Vegfa*-R	ACTCCAGGGCTTCATCGTTA	qRT-PCR
h*Hif1α*-F	CCATTAGAAAGCAGTTCCGC	qRT-PCR
h*Hif1α*-R	TGGGTAGGAGATGGAGATGC	qRT-PCR
m*Hif1α*-F	TGGCTCCCTATATCCCAATG	qRT-PCR
m*Hif1α*-R	GGTCTGCTGGAACCCAGTAA	qRT-PCR
h*GLUT1*-F	CTTTGTGGCCTTCTTTGAAGT	qRT-PCR
h*GLUT1*-R	CCACACAGTTGCTCCACAT	qRT-PCR
h*β-Actin*-F	AGAGCTAGCTGCCTGAC	qRT-PCR
h*β-Actin*-R	GGATGCCACAGGACTCCA	qRT-PCR
h*GAPDH*-F	AGAAGGCTGGGGCTCATTTG	qRT-PCR
h*GAPDH*-R	AGGGGCCATCCACAGTCTTC	qRT-PCR
h*SIRT7*-F	ATGAGCAGAAGCTGGTGC	qRT-PCR
h*SIRT7*-R	CTGTCTGGTGTCTGTGGA	qRT-PCR
h*HK2*-F	TGGAGATGGAGAATCAGA	qRT-PCR
h*HK2*-R	CCAGGAAACTCTCGTCTA	qRT-PCR
h*PKM2*-F	TCGGAGGTTTGATGAAAT	qRT-PCR
h*PKM2*-R	TCTCCAGCATCTGAGTAG	qRT-PCR
SIRT7-111Ser-Ala-F	TCTACACAGGCGCGGGAATCGCCACGGCAGCGTCTATC	Site-mutation
SIRT7-111Ser-Ala-R	GATAGACGCTGCCGTGGCGATTCCCGCGCCTGTGTAGA	Site-mutation
SIRT7-284Thr-Ala-F	CCCACGCCTCTGGTGCATGGCCAAGCCCCCTAGCCGGC	Site-mutation
SIRT7-284Thr-Ala-R	GCCGGCTAGGGGGCTTGGCCATGCACCAGAGGCGTGGG	Site-mutation
SIRT7-359Ser-Ala-F	AGGCAGCCACAGTCGGAAGGCGCTGTGCAGAAGCAG	Site-mutation
SIRT7-359Ser-Ala-R	CTGCTTCTGCACAGCGCCTTCCGACTGTGGCTGCCT	Site-mutation
SIRT7-255Thr-Ala-F	TGGGAAGCGGCGGCCGAGGCTGCCAGCAG	Site-mutation
SIRT7-255Thr-Ala-R	CTGCTGGCAGCCTCGGCCGCCGCTTCCCA	Site-mutation
SIRT7-259Ser-Ala-F	CGACCGAGGCTGCCGCCAGAGCAGACACC	Site-mutation
SIRT7-259Ser-Ala-R	GGTGTCTGCTCTGGCGGCAGCCTCGGTCG	Site-mutation

Targets	Sequence (5'-3')	Purpose
SIRT7-263Thr-Ala-F	CCAGCAGAGCAGACGCCATCCTGTGTCTAGG	Site-mutation
SIRT7-263Thr-Ala-R	CCTAGACACAGGATGGCGTCTGCTCTGCTGG	Site-mutation
SIRT7-255Thr-Glu-F	TGGGAAGCGGCGGAAGAGGCTGCCAGCAG	Site-mutation
SIRT7-255Thr-Glu-R	CTGCTGGCAGCCTCTTCCGCCGCTTCCCA	Site-mutation
SIRT7-259Ser-Glu-F	CGACCGAGGCTGCCGAGAGAGCAGACACC	Site-mutation
SIRT7-259Ser-Glu-R	GGTGTCTGCTCTCTCGGCAGCCTCGGTCG	Site-mutation
SIRT7-263Thr-Asp-F	GGCTGCCAGCAGAGCAGACGACATCCTGTGTCTAGGGTC	Site-mutation
SIRT7-263Thr-Asp-R	GACCCTAGACACAGGATGTCGTCTGCTCTGCTGGCAGCC	Site-mutation
GSK3β-9-Ser-Ala-F	GGCCCAGAACCACCGCCTTTGCGGAGAGCTG	Site-mutation
GSK3β-9-Ser-Ala-R	CAGCTCTCCGCAAAGGCGGTGGTTCTGGGCC	Site-mutation
GSK3β-96-Arg-Ala-F	CAAGAGATTTAAGAATGCAGAGCTCCAGATC	Site-mutation
GSK3β-96-Arg-Ala-R	GATCTGGAGCTCTGCATTCTTAAATCTCTTG	Site-mutation
GSK3β-K85M/K86I-F	GAACTGGTCGCCATCATGATAGTATTGCAGG	Site-mutation
GSK3β-K85M/K86I-R	CCTGCAATACTATCATGATGGCGACCAGTTC	Site-mutation
