# Supplementary material for: Combined intermittent fasting and ERK inhibition enhance the anti-tumor effects of chemotherapy via the GSK3β-SIRT7 axis
Source: Nat Commun. 2021 Aug 25;12:5058. doi: 10.1038/s41467-021-25274-3 (PMC8387475; doi:10.1038/s41467-021-25274-3)
Supplement: Supplementary file 2 — Reporting Summary [file 41467_2021_25274_MOESM2_ESM.pdf]

nature research

Corresponding author(s): Baohua Liu

Last updated by author(s): Jul 6, 2021

Reporting Summary

Nature Research wishes to improve the reproducibility of the work that we publish. This form provides structure for consistency and transparency in reporting. For further information on Nature Research policies, see our Editorial Policies and the Editorial Policy Checklist.

Statistics

For all statistical analyses, confirm that the following items are present in the figure legend, table legend, main text, or Methods section.

n/a	Confirmed	
☐	☒	The exact sample size (*n*) for each experimental group/condition, given as a discrete number and unit of measurement
☐	☒	A statement on whether measurements were taken from distinct samples or whether the same sample was measured repeatedly
☐	☒	The statistical test(s) used AND whether they are one- or two-sided<br>*Only common tests should be described solely by name; describe more complex techniques in the Methods section.*
☒	☐	A description of all covariates tested
☒	☐	A description of any assumptions or corrections, such as tests of normality and adjustment for multiple comparisons
☐	☒	A full description of the statistical parameters including central tendency (e.g. means) or other basic estimates (e.g. regression coefficient) AND variation (e.g. standard deviation) or associated estimates of uncertainty (e.g. confidence intervals)
☐	☒	For null hypothesis testing, the test statistic (e.g. *F*, *t*, *r*) with confidence intervals, effect sizes, degrees of freedom and *P* value noted<br>*Give P values as exact values whenever suitable.*
☒	☐	For Bayesian analysis, information on the choice of priors and Markov chain Monte Carlo settings
☒	☐	For hierarchical and complex designs, identification of the appropriate level for tests and full reporting of outcomes
☒	☐	Estimates of effect sizes (e.g. Cohen's *d*, Pearson's *r*), indicating how they were calculated

Our web collection on statistics for biologists contains articles on many of the points above.

Software and code

Policy information about availability of computer code

Data collection	Immunoblots data were collected by ChemiDoc Touch Imaging System (BIO-RAD).<br>IHC images were collected by BX43 (OLYMPUS).<br>Real-time PCR data were collected by BIO-RAD CFX Connect (BIO-RAD).<br>Cell viability measured by CCK8 kit assay was collected by EnSpire (Perkin Elmer Life Sciences).
Data analysis	Image Lab Software V5.2.1 build 11 (Bio-Rad) was used for immunoblotting analyses.<br>Excel (Microsoft Office) were used for statistical analyses.<br>Image J software was used for quantification analyses (V1.52)

For manuscripts utilizing custom algorithms or software that are central to the research but not yet described in published literature, software must be made available to editors and reviewers. We strongly encourage code deposition in a community repository (e.g. GitHub). See the Nature Research guidelines for submitting code & software for further information.

Data

Policy information about availability of data

All manuscripts must include a data availability statement. This statement should provide the following information, where applicable:

- Accession codes, unique identifiers, or web links for publicly available datasets
- A list of figures that have associated raw data
- A description of any restrictions on data availability

Patient's survival data were obtained from the online tool (https://kmplot.com/analysis). GSEA analysis was based on the published data GSE99596. SIRT7 phosphorylation residues were predicated by GPS 5.0 (http://gps.biocuckoo.cn/). SIRT7 missense and insertion mutations were identified from COSMIC database (https://cancer.sanger.ac.uk/cosmic/search?q=sirt7). The other data supporting the findings of this study are available in the main figures, supplementary

information and the source data file. Source data are provided with this paper.

Field-specific reporting

Please select the one below that is the best fit for your research. If you are not sure, read the appropriate sections before making your selection.

- [x] Life sciences
- [ ] Behavioural & social sciences
- [ ] Ecological, evolutionary & environmental sciences

For a reference copy of the document with all sections, see nature.com/documents/nr-reporting-summary-flat.pdf

Life sciences study design

All studies must disclose on these points even when the disclosure is negative.

Sample size	No particular statistical methods were used to predetermine sample size. Our chosen sample sizes were similar to those generally employed and accepted in the field (Wei Wang et al., 2021; Yi Bao et al., 2020; Stephanie P. et al., 2021). All sample sizes are shown in the figures legends or in the methods section of our manuscript, respectively.
Data exclusions	Some dead animals owing to experiment-independent causes (e.g. fasting) at early stage in the experiment were removed.
Replication	All experiments were performed with at least three biological replicates and confirmed from more than two independent experiments.
Randomization	For analysis from cell culture, we plated cells in a random distribution and randomly assigned them to experimental groups. For in vivo analysis from animal model, all mice initially were randomized into different experimental groups.
Blinding	The data collection and analysis were not performed blind due to obvious differences between groups. However, two observers performed the experiments and independently analyzed the data.

Reporting for specific materials, systems and methods

We require information from authors about some types of materials, experimental systems and methods used in many studies. Here, indicate whether each material, system or method listed is relevant to your study. If you are not sure if a list item applies to your research, read the appropriate section before selecting a response.

Materials & experimental systems

n/a	Involved in the study	
☐	☒	Antibodies
☐	☒	Eukaryotic cell lines
☒	☐	Palaeontology and archaeology
☐	☒	Animals and other organisms
☒	☐	Human research participants
☒	☐	Clinical data
☒	☐	Dual use research of concern

Methods

n/a	Involved in the study	
☒	☐	ChIP-seq
☒	☐	Flow cytometry
☒	☐	MRI-based neuroimaging

Antibodies

Antibodies used

SIRT7, Santa Cruz (sc-365344) , WB/IP (1:3,000/1:100);
SIRT7, EMD Millipore (ABE103), IHC (1:50);
(pSer9)-GSK3β, CST (#9322), WB (1:1,000);
GSK3β, CST (#12456), WB (1:1,000);
GSK3α/β, CST (#5676), WB (1:100);
(pSer473)-AKT, CST (#4060S), WB (1:1,000);
(pThr308)-AKT , CST (#13038), WB (1:1,000);
AKT, CST (#4691), WB (1:1,000);
(pThr202/Tyr204) ERK1/2, CST(#4370S) , WB (1:1,000);
ERK1/2, CST (#4695S), WB (1:1,000);
(pThr172)-AMPK, CST (#50081), WB (1:1,000);
AMPKα1/2, Abcam (ab80039), WB (1:2,000);
(pSer79)-ACC, CST(#3661), WB (1:1,000);
ACC, CST (#3676), WB (1:1,000);
Phospho-serine/threonine, ECM biosciences (PM3801) , WB (1:1,000);
His-tag, Proteintech (66005-1), WB/IP (1:5,000/1:500);
HA-tag, Sigma–Aldrich (H3663), WB (1:5,000);
FLAG-tag, Sigma–Aldrich (F3165), WB(1:5,000);

α-Tubulin, Beyotime (AT819) , WB (1:5,000);
GAPDH, Beyotime (AG019), WB (1:5,000);
Anti-rabbit IgG, Jackson (11-035-003), WB (1:10,000);
Anti-mouse IgG, Jackson (15-035-003), WB (1:10,000);
UBR5, CST (#65344), WB (1:1,000);
EDD1, Abcam (ab70311), IP (1:500);
HIF1α, Novus Biologicals (NB100-105), WB (1:500);
SKP2, CST (#2652), WB (1:1,000);
SKP1, CST (#12248), WB (1:1,000);
CUL1, CST (#4995), WB (1:1,000);
FKBP51, CST (#12210), WB (1:1,000);
K63-linkage Specific Polyubiquitin, CST (#12930), WB (1:500);
K48-linkage Specific Polyubiquitin, CST (#12805), WB (1:500).

Validation

All these antibodies below are commercially available and all validated by the producers.
SIRT7, Santa Cruz (sc-365344) , WB/IP (1:3,000/1:100);
https://www.scbt.com/p/sirt7-antibody-c-3;
SIRT7, EMD Millipore (ABE103), IHC (1:50);
https://www.merckmillipore.com/CN/zh/product/Anti-SIRT7-Antibody,MM_NF-ABE103?ReferrerURL=https%3A%2F%2Faether.shuu.cf%2F;
(pSer9)-GSK3β, CST (#9322), WB (1:1,000);
https://www.cellsignal.cn/products/primary-antibodies/gsk-3b-d5c5z-xp-rabbit-mab/12456;
GSK3β, CST (#12456), WB (1:1,000);
https://www.cellsignal.cn/products/primary-antibodies/gsk-3b-d5c5z-xp-rabbit-mab/12456;
GSK3α/β, CST (#5676), WB (1:100);
https://www.cellsignal.cn/products/primary-antibodies/gsk-3a-b-d75d3-rabbit-mab/5676?site-search-type=Products&N=4294956287&Ntt=5676&fromPage=plp&_requestid=3120249;
(pSer473)-AKT, CST (#4060S), WB (1:1,000);
https://www.cellsignal.cn/products/primary-antibodies/phospho-akt-ser473-d9e-xp-rabbit-mab/4060?site-search-type=Products&N=4294956287&Ntt=4060s&fromPage=plp&_requestid=3120290;
(pThr308)-AKT , CST (#13038), WB (1:1,000);
https://www.cellsignal.cn/products/primary-antibodies/phospho-akt-thr308-d25e6-xp-rabbit-mab/13038?site-search-type=Products&N=4294956287&Ntt=13038&fromPage=plp&_requestid=3120312;
AKT, CST (#4691), WB (1:1,000);
https://www.cellsignal.cn/products/primary-antibodies/akt-pan-c67e7-rabbit-mab/4691?site-search-type=Products&N=4294956287&Ntt=4691&fromPage=plp&_requestid=3105784;
(pThr202/Tyr204) ERK1/2, CST(#4370S) , WB (1:1,000);
https://www.cellsignal.cn/products/primary-antibodies/phospho-p44-42-mapk-erk1-2-thr202-tyr204-d13-14-4e-xp-rabbit-mab/4370?site-search-type=Products&N=4294956287&Ntt=4370&fromPage=plp;
ERK1/2, CST (#4695S), WB (1:1,000);
https://www.cellsignal.cn/products/primary-antibodies/p44-42-mapk-erk1-2-137f5-rabbit-mab/4695?site-search-type=Products&N=4294956287&Ntt=4695&fromPage=plp;
(pThr172)-AMPK, CST (#50081), WB (1:1,000);
https://www.cellsignal.cn/products/primary-antibodies/phospho-ampka-thr172-d4d6d-rabbit-mab/50081?site-search-type=Products&N=4294956287&Ntt=50081&fromPage=plp&_requestid=3120470;
AMPKα1/2, Abcam (ab80039), WB (1:2,000);
https://www.abcam.com/ampk-alpha-1-ampk-alpha-2-antibody-342-ab80039.html;
(pSer79)-ACC, CST(#3661), WB (1:1,000);
https://www.cellsignal.cn/products/primary-antibodies/phospho-acetyl-coa-carboxylase-ser79-antibody/3661?site-search-type=Products&N=4294956287&Ntt=3661&fromPage=plp&_requestid=3120534;
ACC, CST (#3676), WB (1:1,000);
https://www.cellsignal.cn/products/primary-antibodies/acetyl-coa-carboxylase-c83b10-rabbit-mab/3676?site-search-type=Products&N=4294956287&Ntt=3676&fromPage=plp&_requestid=3120567;
Phospho-serine/threonine, ECM biosciences (PM3801), WB (1:1,000);
https://ecmbio.com/products/pm3801;
His-tag, Proteintech (66005-1), WB/IP (1:5,000/1:500);
https://www.ptglab.com/products/His-Tag-Antibody-66005-1-Ig.htm;
HA-tag, Sigma–Aldrich (H3663), WB (1:5,000);
https://www.sigmaaldrich.cn/CN/en/product/sigma/h3663?context=product;
FLAG-tag, Sigma–Aldrich (F3165), WB(1:5,000);
https://www.sigmaaldrich.cn/CN/en/product/sigma/f3165?context=product;
α-Tubulin, Beyotime (AT819) , WB (1:5,000);
https://m.beyotime.com/mobilegoods.do?method=code&code=AT819;
GAPDH, Beyotime (AG019), WB (1:5,000);
https://m.beyotime.com/mobilegoods.do?method=code&code=AG019;
Anti-rabbit IgG, Jackson (11-035-003), WB (1:10,000);
https://www.jacksonimmuno.com/catalog/products/111-035-003;
Anti-mouse IgG, Jackson (15-035-003), WB (1:10,000);
https://www.jacksonimmuno.com/catalog/products/115-035-003;
UBR5, CST (#65344), WB (1:1,000);

https://www.cellsignal.cn/products/primary-antibodies/ubr5-d6o8z-rabbit-mab/65344?site-search-type=Products&N=4294956287&Ntt=65344&fromPage=plp&_requestid=3121453;
EDD1, Abcam (ab70311), IP (1:500);
https://www.abcam.com/edd-antibody-ab70311.html;
HIF1α, Novus Biologicals (NB100-105), WB (1:500);
https://www.novusbio.com/products/hif-1-alpha-antibody-h1alpha67_nb100-105;
SKP2, CST (#2652), WB (1:1,000);
https://www.cellsignal.cn/products/primary-antibodies/skp2-d3g5-xp-rabbit-mab/2652?site-search-type=Products&N=4294956287&Ntt=2652&fromPage=plp&_requestid=3121573;
SKP1, CST (#12248), WB (1:1,000);
https://www.cellsignal.cn/products/primary-antibodies/skp1-d3j4n-rabbit-mab/12248?site-search-type=Products&N=4294956287&Ntt=12248&fromPage=plp&_requestid=3121601;
CUL1, CST (#4995), WB (1:1,000);
https://www.cellsignal.cn/products/primary-antibodies/cul1-antibody/4995?site-search-type=Products&N=4294956287&Ntt=4995&fromPage=plp&_requestid=3121623;
FKBP51, CST (#12210), WB (1:1,000);
https://www.cellsignal.cn/products/primary-antibodies/fkbp5-d5g2-rabbit-mab/12210?site-search-type=Products&N=4294956287&Ntt=12210&fromPage=plp&_requestid=3121648;
K63-linkage Specific Polyubiquitin, CST (#12930), WB (1:500);
https://www.cellsignal.cn/products/antibody-conjugates/k63-linkage-specific-polyubiquitin-d7a11-rabbit-mab-hrp-conjugate/12930?site-search-type=Products&N=4294956287&Ntt=12930&fromPage=plp&_requestid=3121721;
K48-linkage Specific Polyubiquitin, CST (#12805), WB (1:500);
https://www.cellsignal.cn/products/antibody-conjugates/k48-linkage-specific-polyubiquitin-d9d5-rabbit-mab-hrp-conjugate/12805?site-search-type=Products&N=4294956287&Ntt=12805&fromPage=plp&_requestid=3121746.
We generated the SIRT7 (p-T255/S259) antibody which was validated in the manuscript shown in supplementary Fig. 3d and 3e.

Eukaryotic cell lines

Policy information about cell lines

Cell line source(s)	HeLa, HEK293, Bt-549, MDA-MB-231, 4T1, MCF-7 and A549 cells were obtained from American Type Culture Collection. NCI-H1975 cells were purchased from Chinese Academy of Sciences Committee Type Culture Collection Cell Bank.
Authentication	All cell lines were routinely authenticated by analysis of cell growth rate and morphology.
Mycoplasma contamination	All cell lines were routinely tested to ensure the free of mycoplasma contamination.
Commonly misidentified lines (See ICLAC register)	None.

Animals and other organisms

Policy information about studies involving animals; ARRIVE guidelines recommended for reporting animal research

Laboratory animals	PyMT;Sirt7+/- mice, C57BL/6J x FVB background, 4 months, females;<br>PyMT;Sirt7+/+ mice, C57BL/6J x FVB background, 4 months, females;<br>SIRT7-transgenic mice, C57BL/6J, 2-6 months, males and females;<br>PyMT;WT, C57BL/6J x FVB background, 4 months, females;<br>PyMT;Sirt7-TG mice, C57BL/6J x FVB background, 4 months, females;<br>Athymic nu/nu mice, BALB/cJ, 6-8 weeks, males;<br>Normal BALB/cJ mice, 6-8 weeks, females.<br>Mice were maintained at 21-23°C, in 40% to 60% humidity, and with a 12h light / 12h dark light cycle.
Wild animals	No wild animals were used in this study.
Field-collected samples	This study did not involve any field-collected samples.
Ethics oversight	All the experimental animals were housed and handled in accordance with protocols approved by the Committee on the Use of Live Animals in Teaching and Research of Shenzhen University.

Note that full information on the approval of the study protocol must also be provided in the manuscript.
