# Supplementary material for: Combined intermittent fasting and ERK inhibition enhance the anti-tumor effects of chemotherapy via the GSK3β-SIRT7 axis
Source: Nat Commun. 2021 Aug 25;12:5058. doi: 10.1038/s41467-021-25274-3 (PMC8387475; doi:10.1038/s41467-021-25274-3)

Fig. 1

Fig. 1a

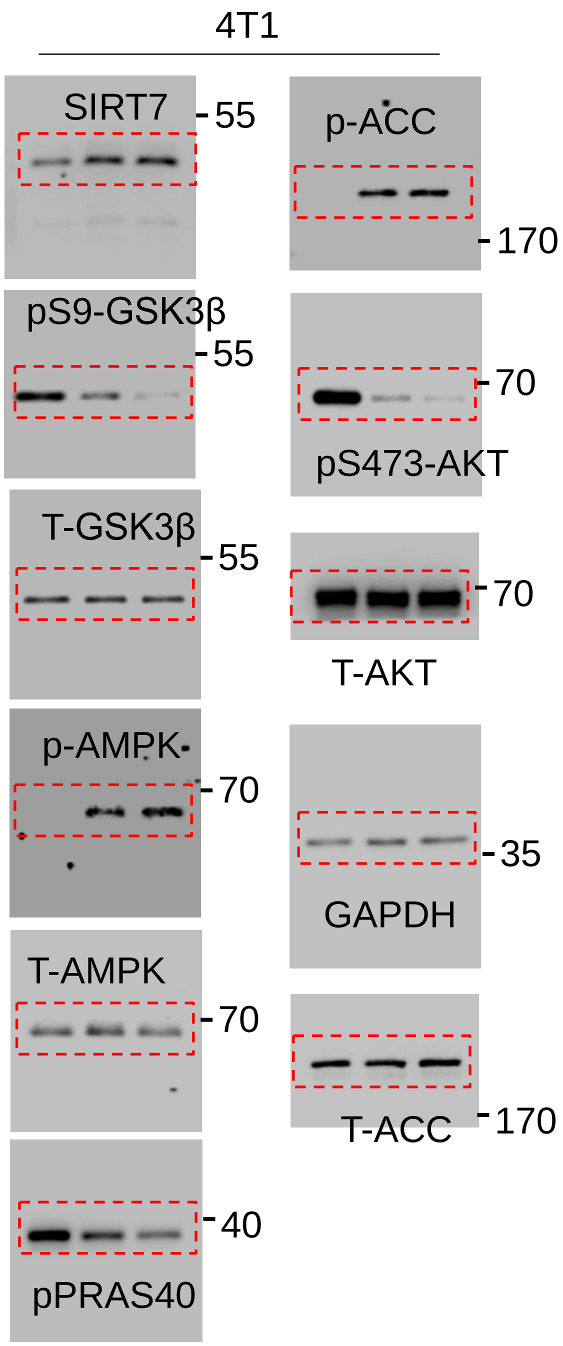

Fig. 1c

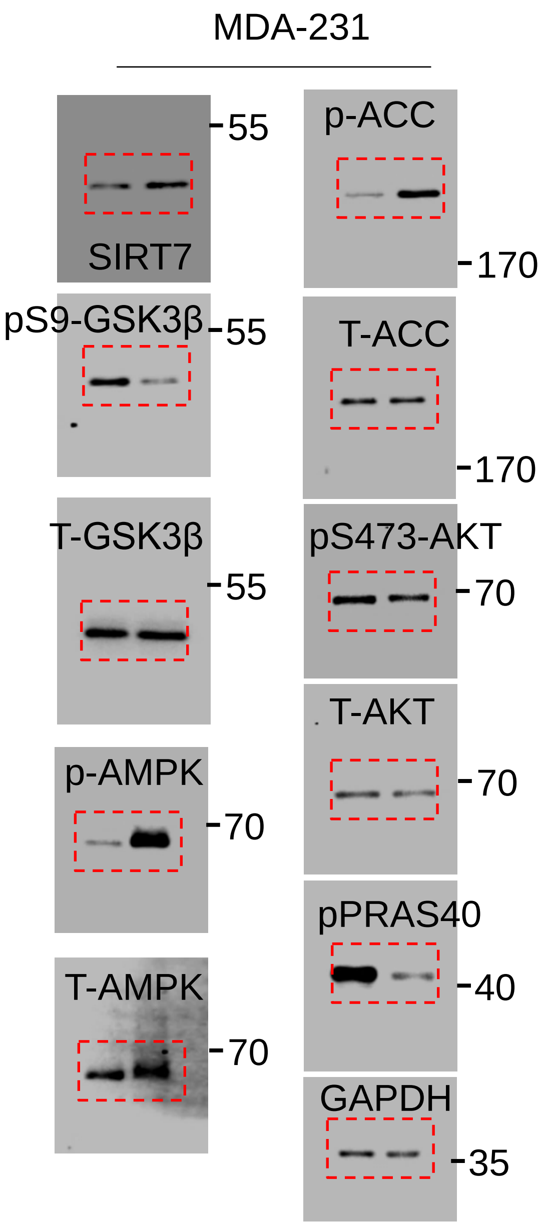

Fig. 1e

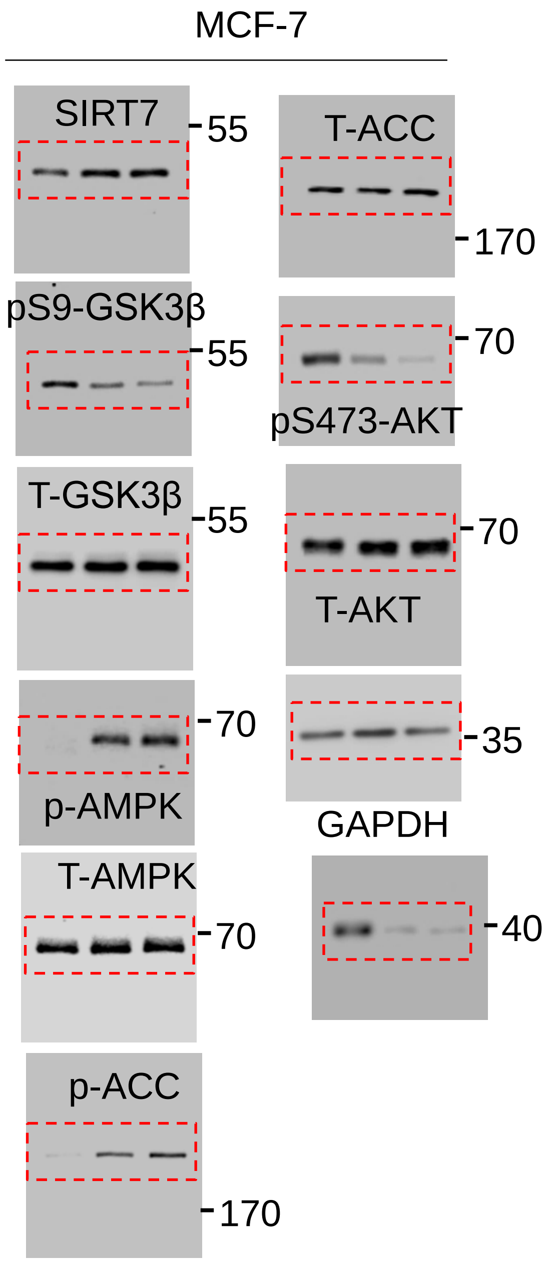

Supplementary Fig. 1

Supplementary Fig. 1a

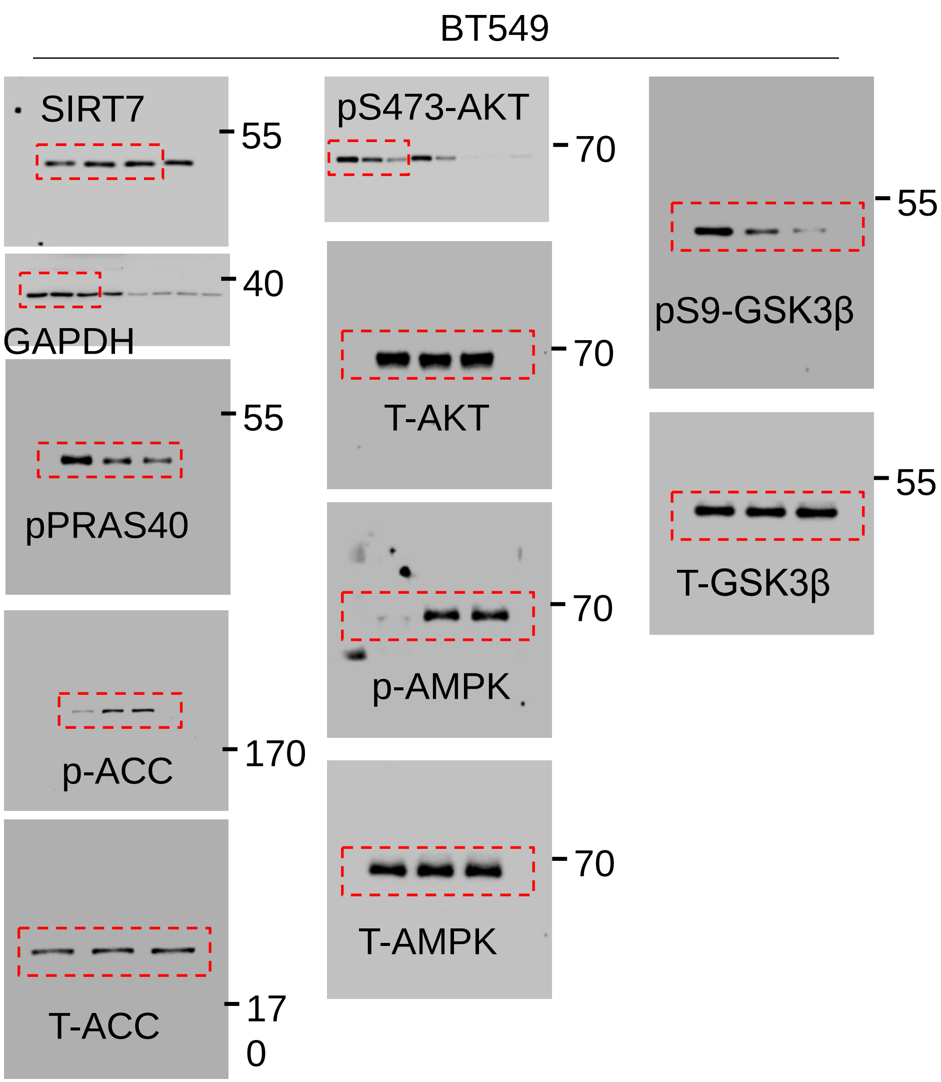

Supplementary Fig. 1b

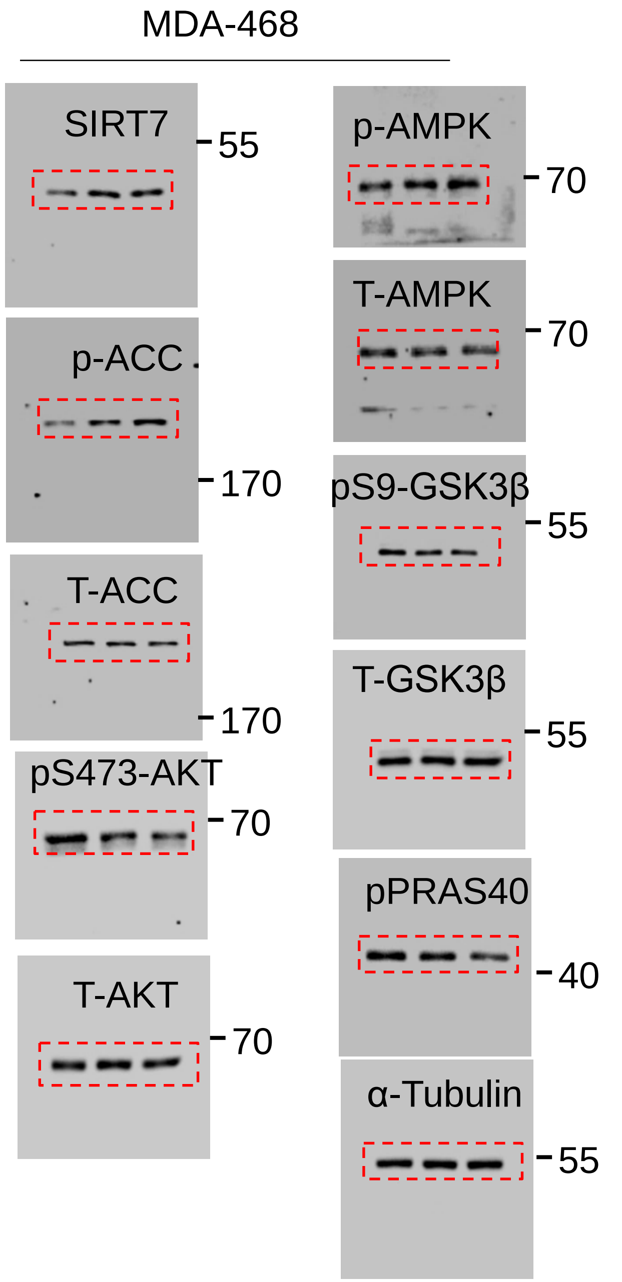

Supplementary Fig. 1c

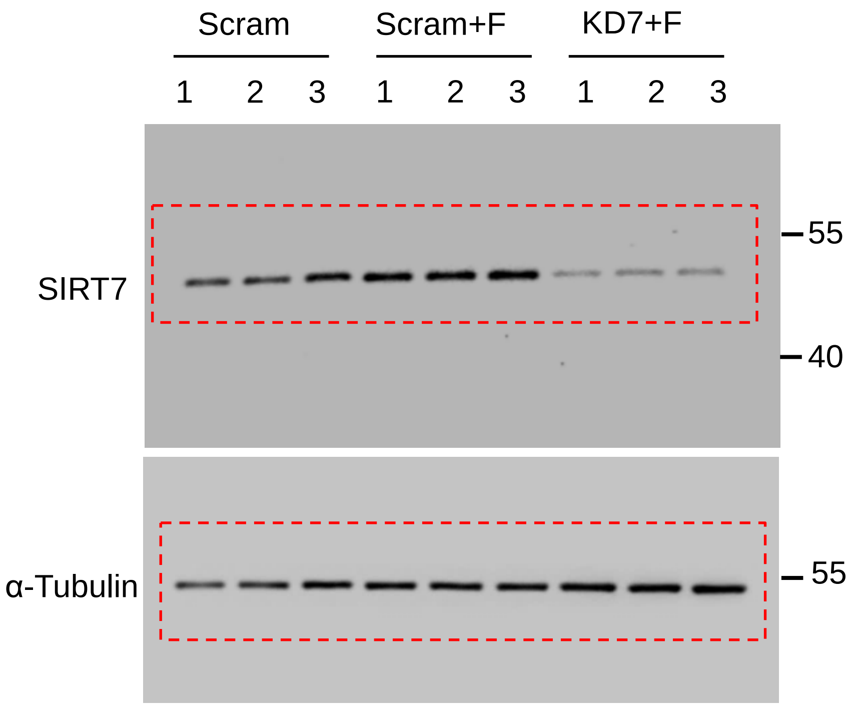

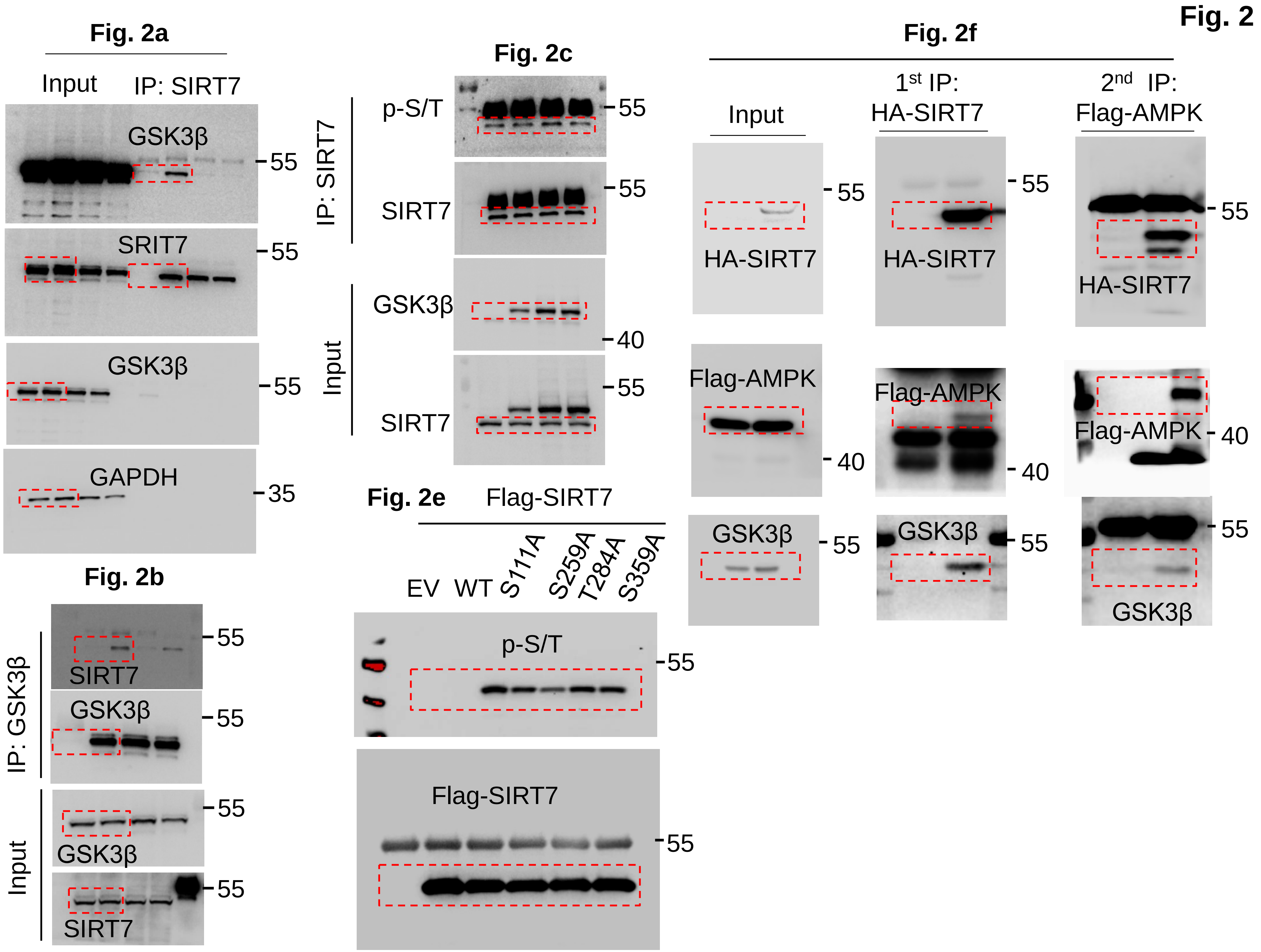
Fig. 2
Fig. 2a
Input
IP: SIRT7
GSK3β
55
SRIT7
55
GSK3β
55
GAPDH
35
Fig. 2b
IP: GSK3β
SIRT7
55
GSK3β
55
Input
GSK3β
55
SIRT7
55
Fig. 2c
IP: SIRT7
p-S/T
55
SIRT7
55
Input
GSK3β
40
SIRT7
55
Fig. 2e
Flag-SIRT7
EV
WT
S111A
S259A
T284A
S359A
p-S/T
55
Flag-SIRT7
55
Fig. 2f
Input
1st IP: HA-SIRT7
2nd IP: Flag-AMPK
HA-SIRT7
55
Flag-AMPK
40
GSK3β
55

Fig. 2

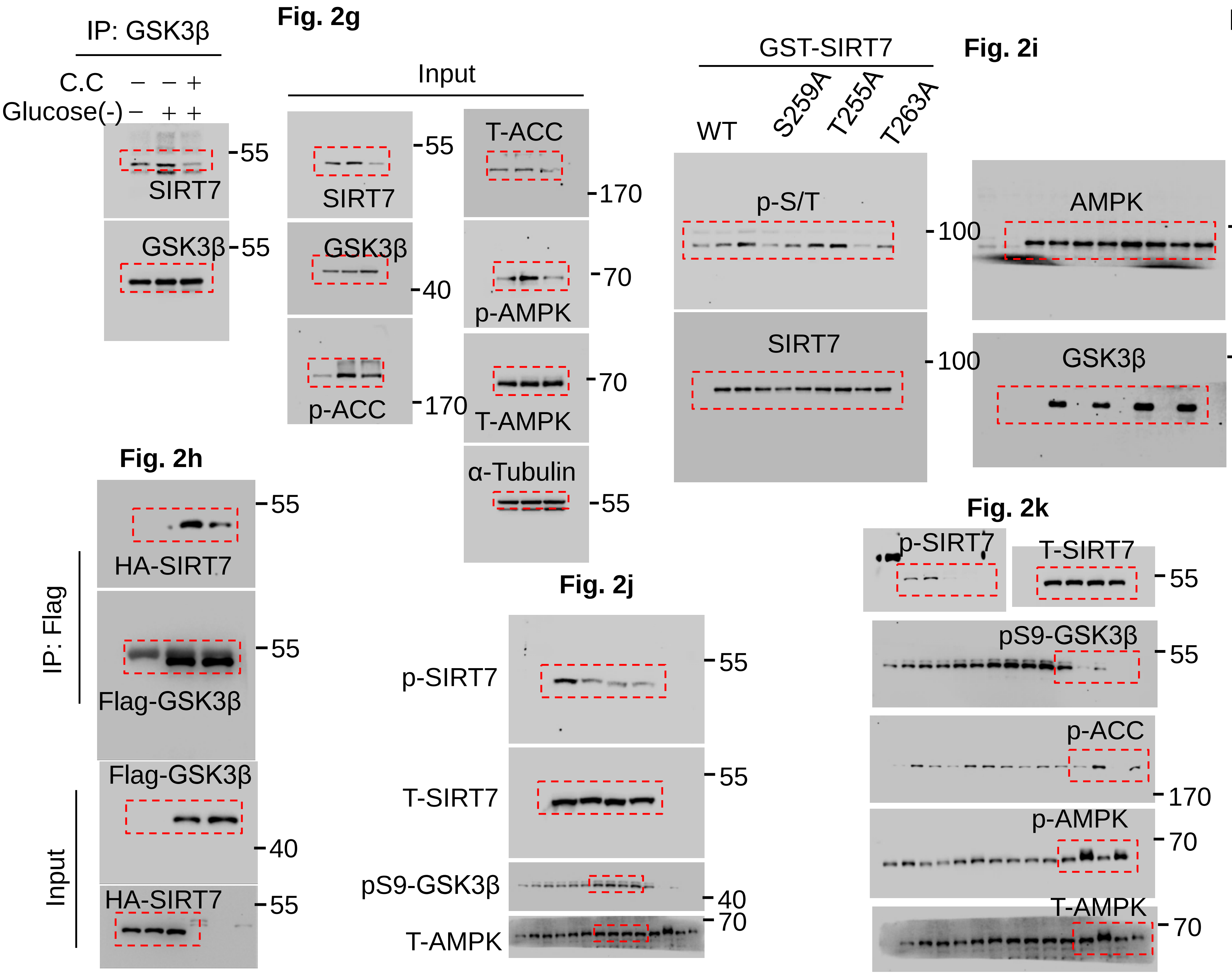

Fig. 2g
IP: GSK3β
C.C
Glucose(-)
SIRT7
GSK3β
55
Input
SIRT7
GSK3β
p-ACC
T-ACC
p-AMPK
T-AMPK
α-Tubulin
55
40
170
70
Fig. 2h
IP: Flag
Input
HA-SIRT7
Flag-GSK3β
Flag-GSK3β
HA-SIRT7
55
40
Fig. 2i
GST-SIRT7
WT
S259A
T255A
T263A
p-S/T
SIRT7
100
AMPK
GSK3β
70
Fig. 2j
p-SIRT7
T-SIRT7
pS9-GSK3β
T-AMPK
55
40
70
Fig. 2k
p-SIRT7
T-SIRT7
pS9-GSK3β
p-ACC
p-AMPK
T-AMPK
55
170
70

Supplementary Fig. 2

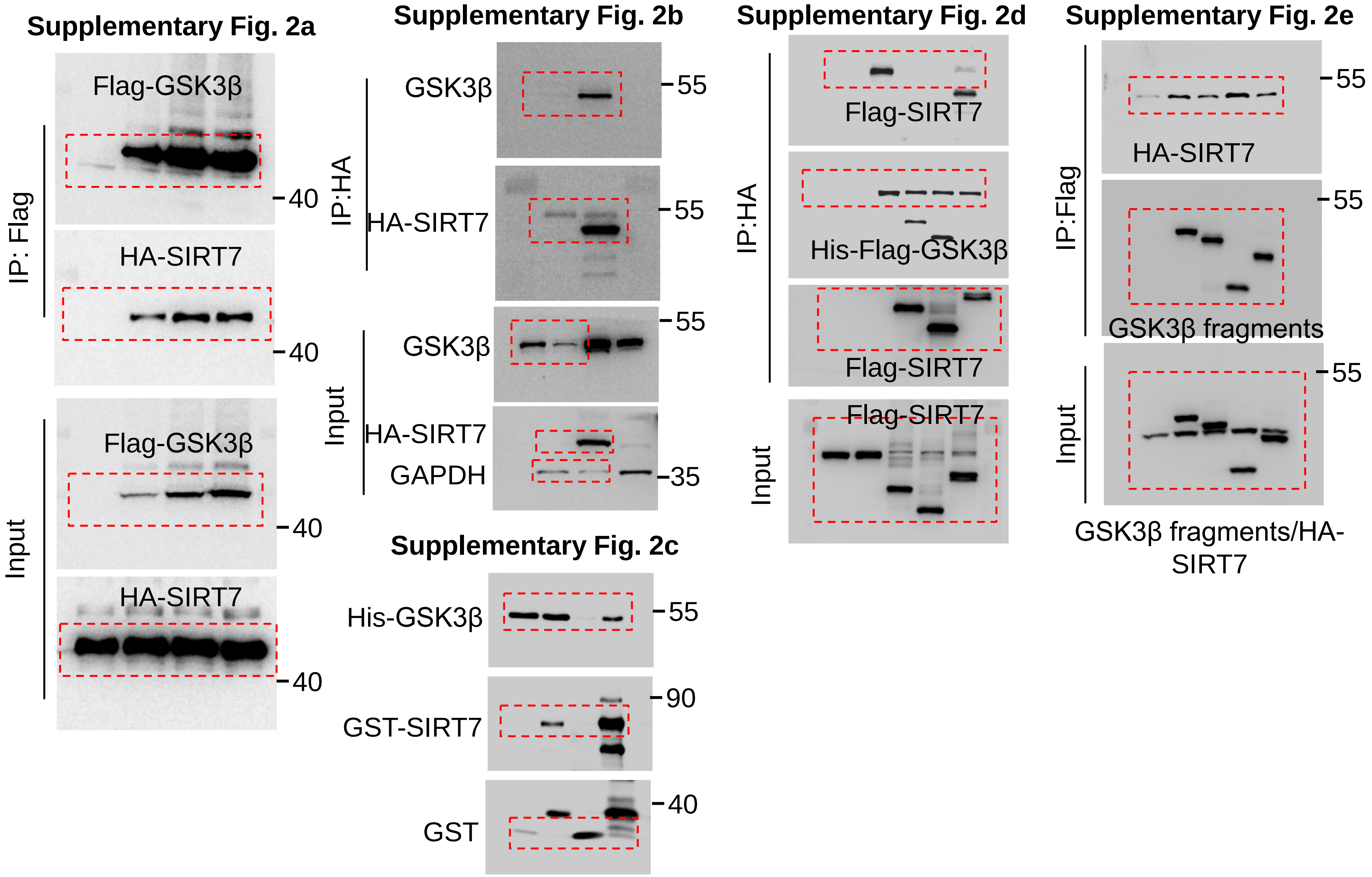

Supplementary Fig. 2

Supplementary Fig. 2f

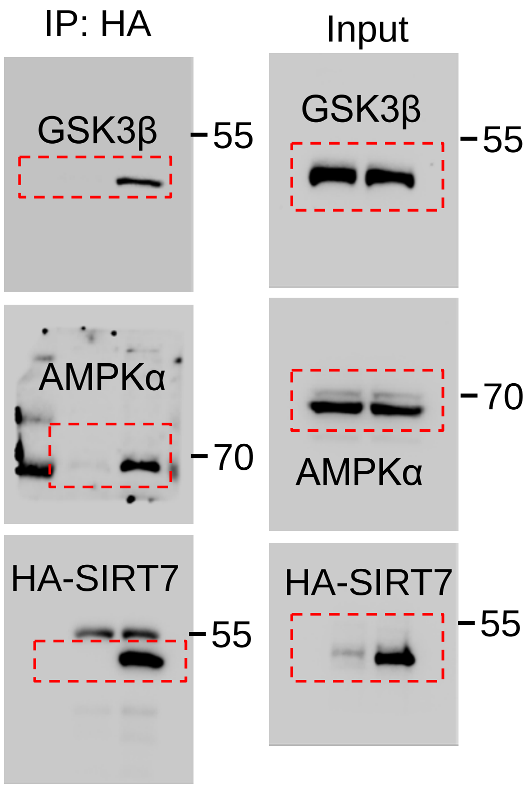

Supplementary Fig. 3

Supplementary Fig. 3d

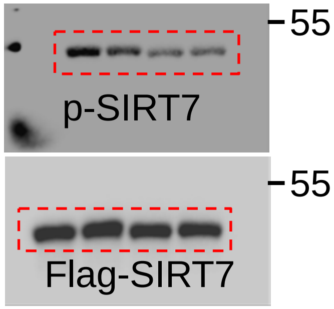

Supplementary Fig. 3e

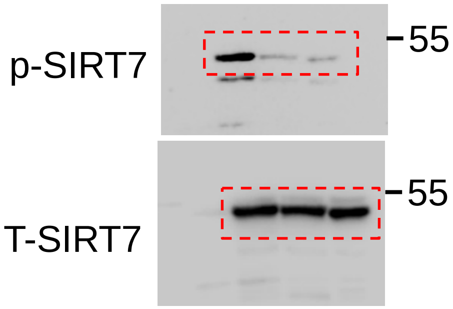

Fig. 3

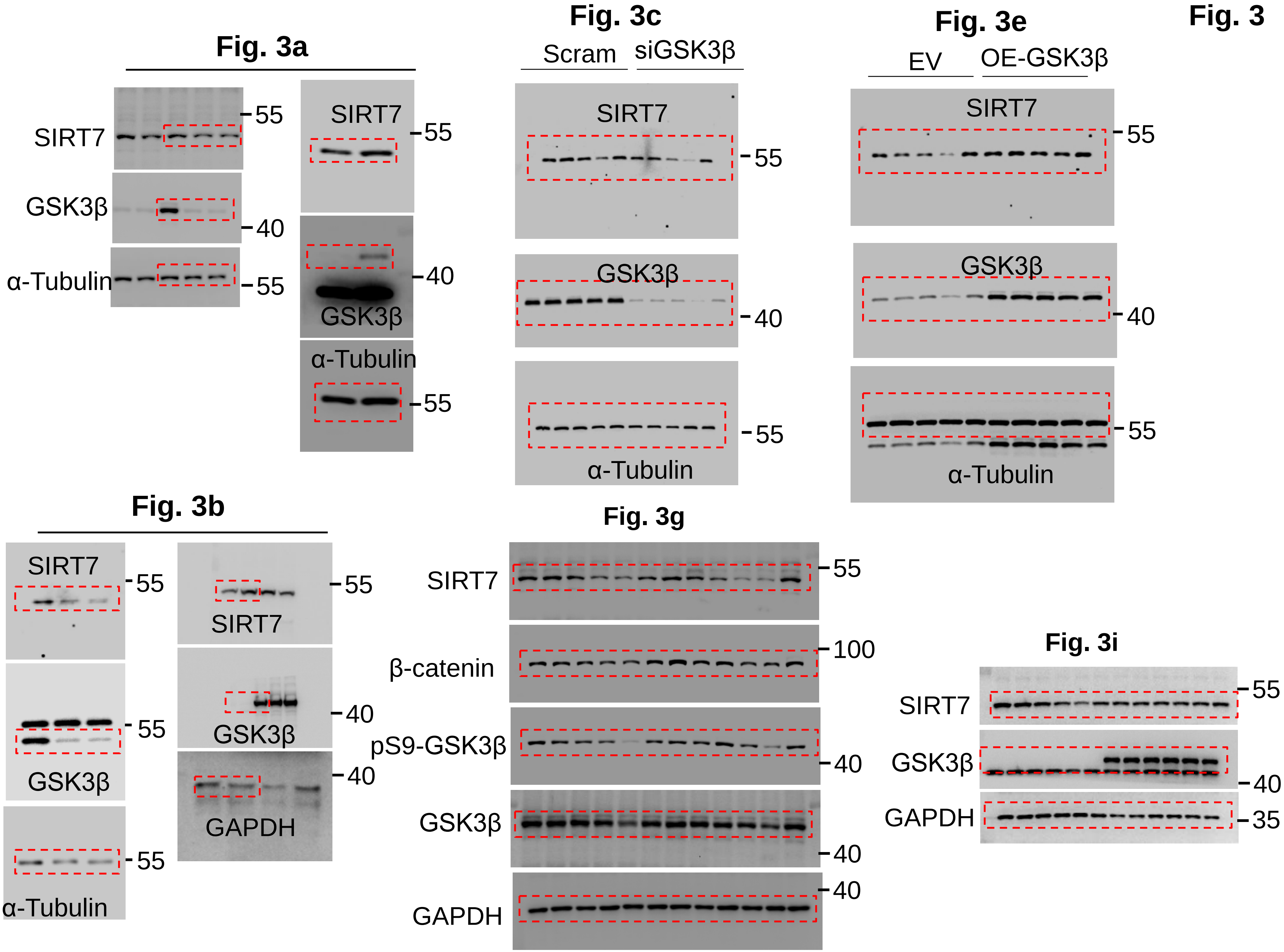
Fig. 3a
SIRT7
GSK3β
α-Tubulin
55
40
55
SIRT7
GSK3β
α-Tubulin
Fig. 3b
SIRT7
GSK3β
α-Tubulin
GAPDH
Fig. 3c
Scram
siGSK3β
SIRT7
GSK3β
α-Tubulin
Fig. 3e
EV
OE-GSK3β
SIRT7
GSK3β
α-Tubulin
Fig. 3g
SIRT7
β-catenin
pS9-GSK3β
GSK3β
GAPDH
100
Fig. 3i
SIRT7
GSK3β
GAPDH
35

Fig. 3

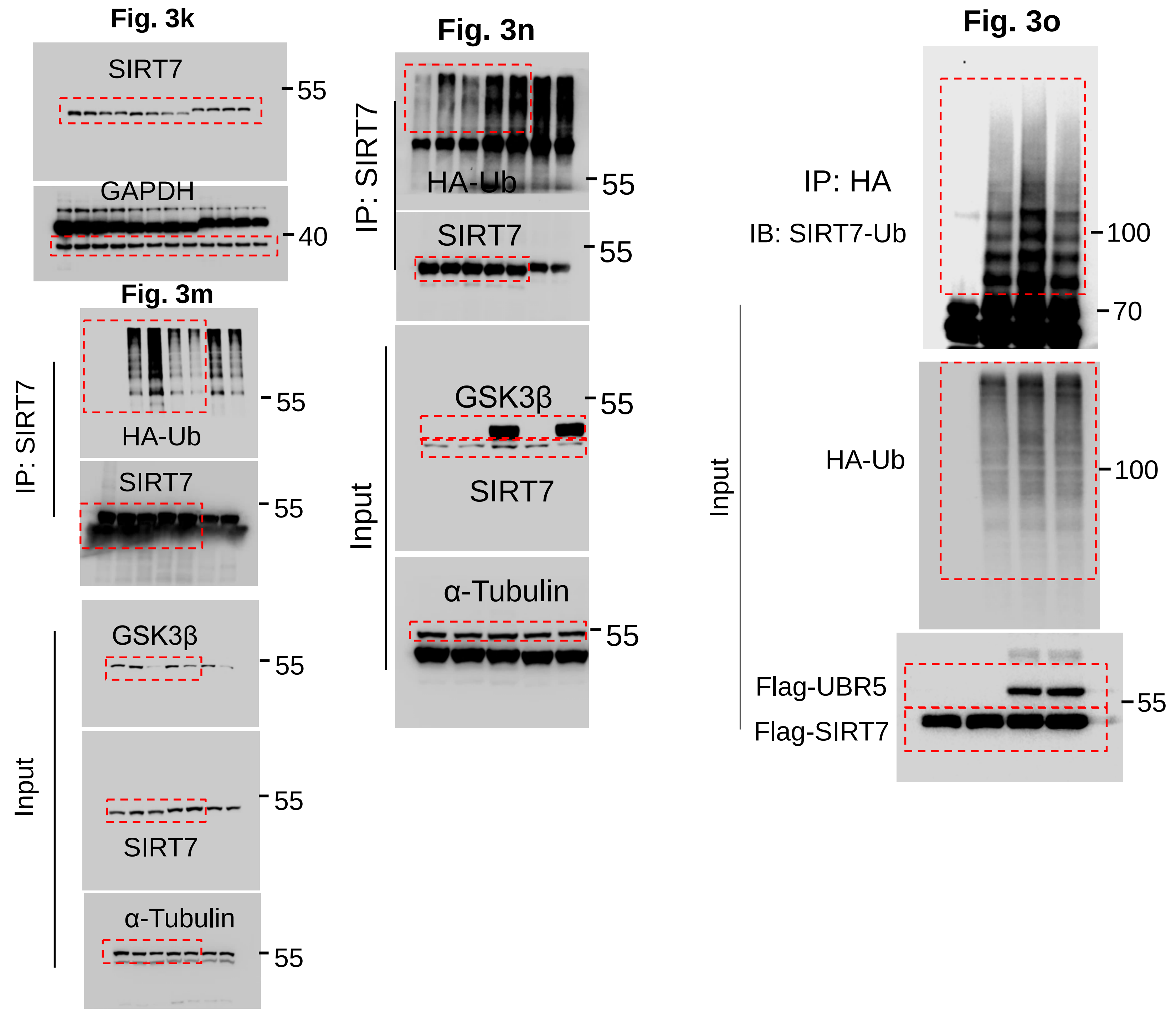

Fig. 3k
SIRT7
55
GAPDH
40
Fig. 3m
IP: SIRT7
HA-Ub
55
SIRT7
55
Input
GSK3β
55
SIRT7
55
α-Tubulin
55
Fig. 3n
IP: SIRT7
HA-Ub
55
SIRT7
55
Input
GSK3β
55
SIRT7
α-Tubulin
55
Fig. 3o
IP: HA
IB: SIRT7-Ub
100
70
Input
HA-Ub
100
Flag-UBR5
Flag-SIRT7
55

Fig. 3

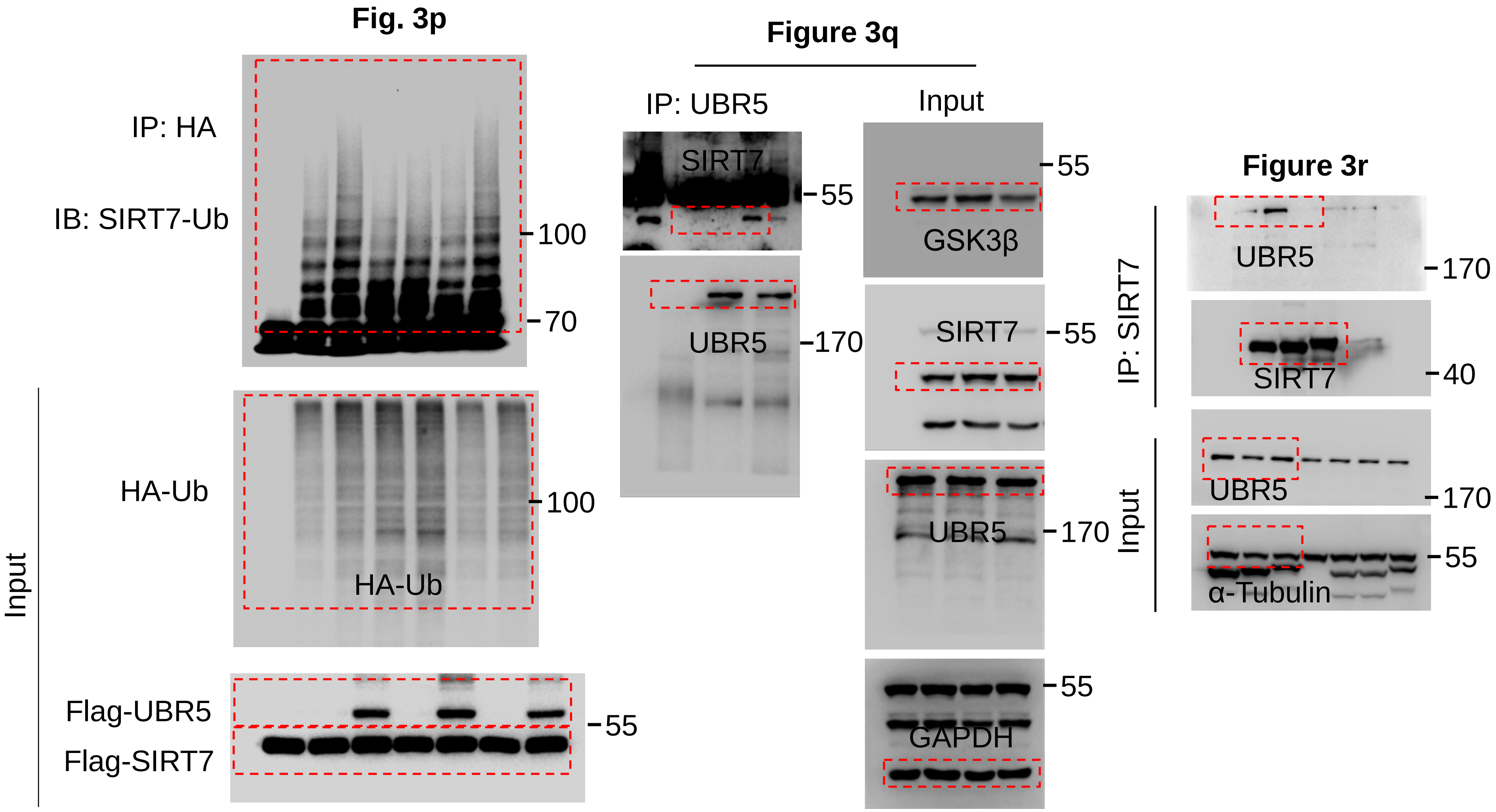
Fig. 3p
IP: HA
IB: SIRT7-Ub
100
70
Input
HA-Ub
HA-Ub
100
Flag-UBR5
Flag-SIRT7
55
Figure 3q
IP: UBR5
Input
SIRT7
55
UBR5
170
55
GSK3β
SIRT7
55
UBR5
170
55
GAPDH
Figure 3r
IP: SIRT7
UBR5
170
SIRT7
40
Input
UBR5
170
α-Tubulin
55

Supplementary Fig. 4

Supplementary Fig. 4a

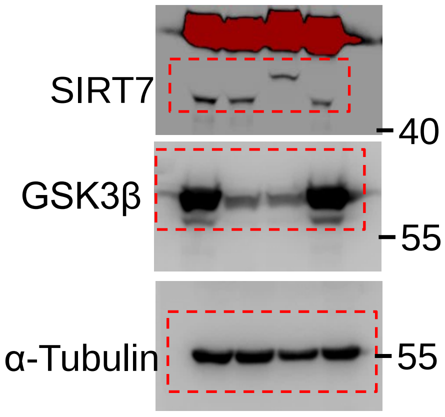

Supplementary Fig. 4e

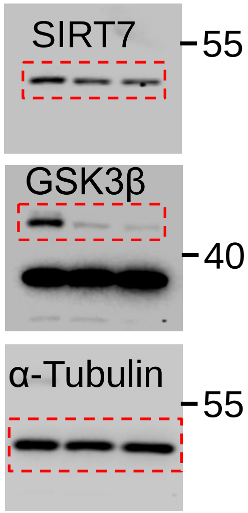

Supplementary Fig. 4f

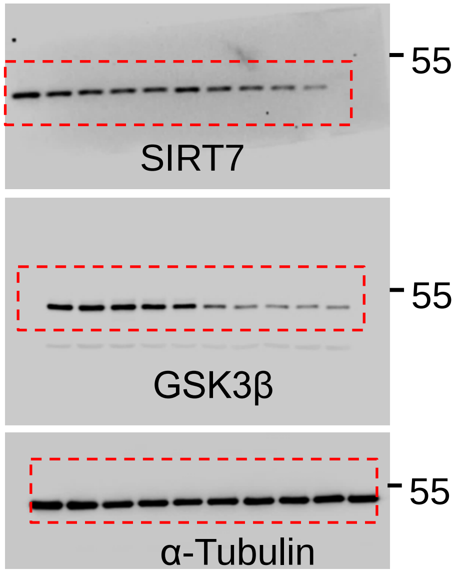

Supplementary Fig. 4c

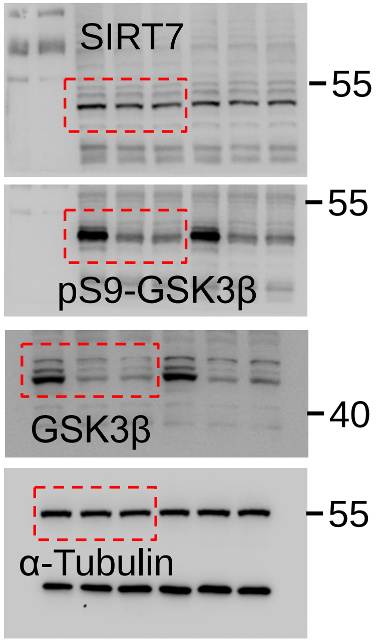

Supplementary Fig. 4g

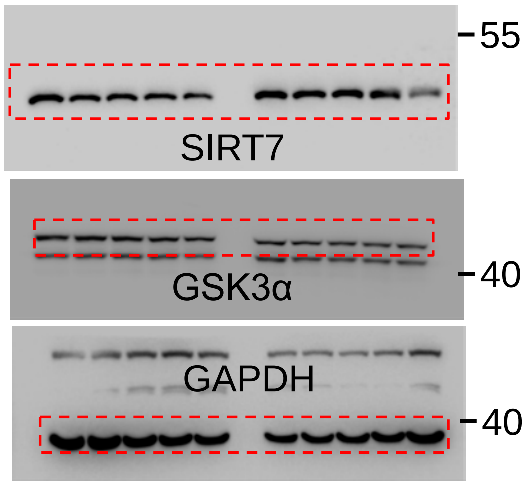

Supplementary Fig. 4h

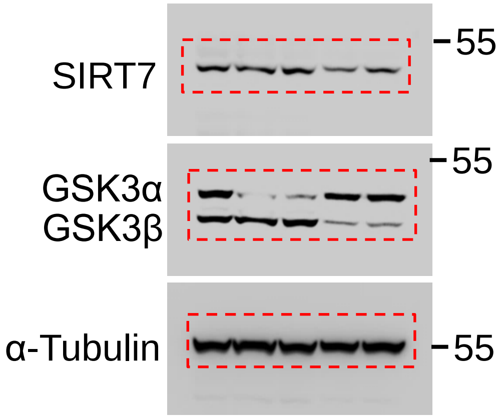

Supplementary Fig. 4

Supplementary Fig. 4i

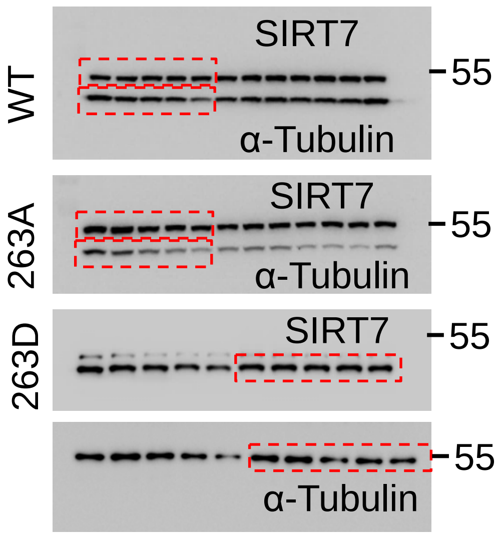

Supplementary Fig. 4l

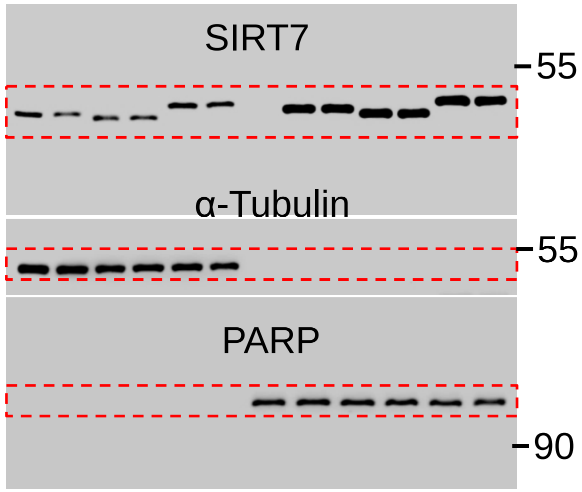

Supplementary Fig. 4m

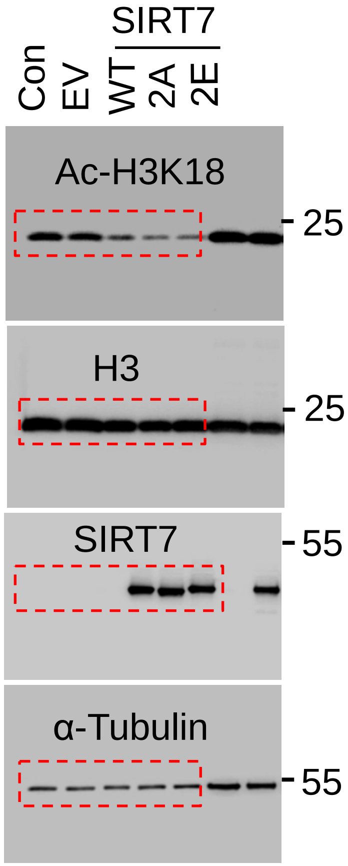

Supplementary Fig. 4k

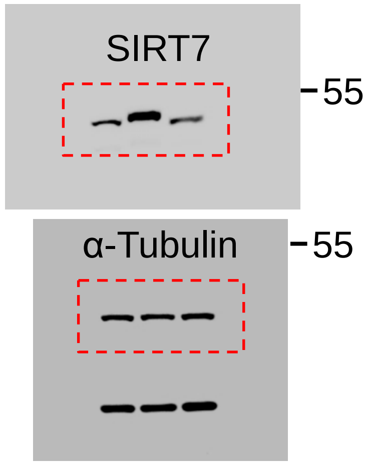

Supplementary Fig. 4n

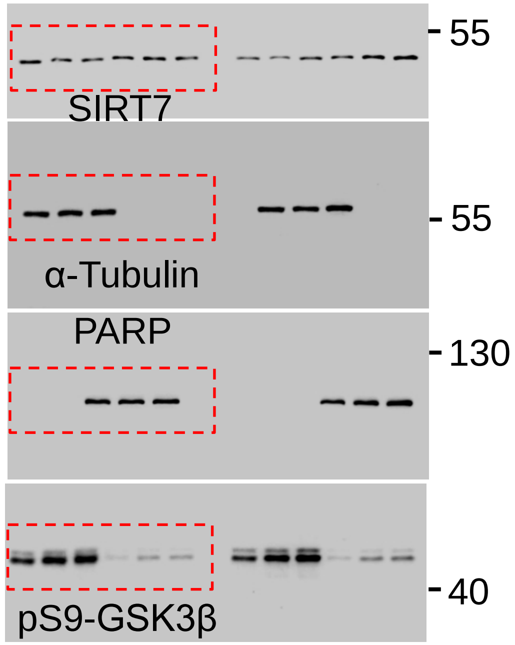

Supplementary Fig. 4o

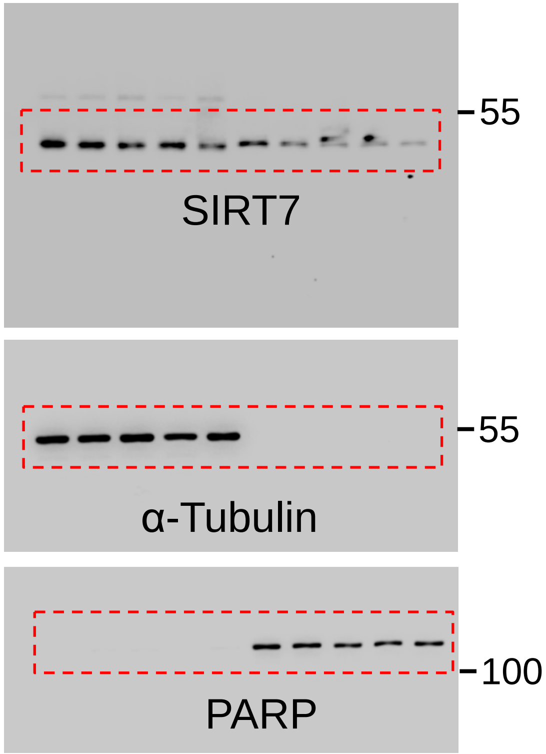

Supplementary Fig. 5

Supplementary Fig. 5a

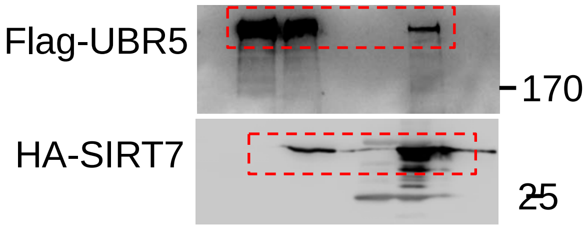

Supplementary Fig. 5b

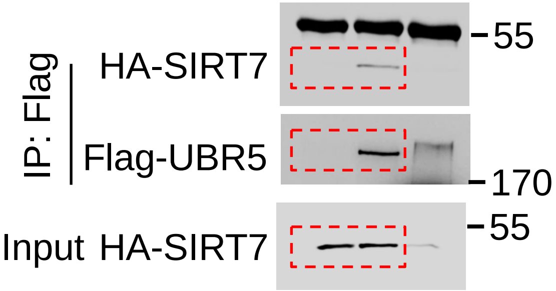

Supplementary Fig. 5e

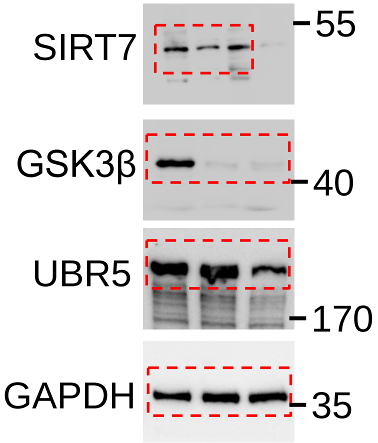

Supplementary Fig. 5c

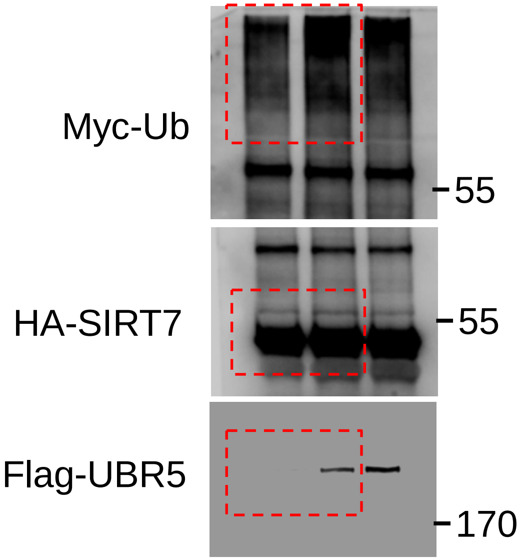

Supplementary Fig. 5g

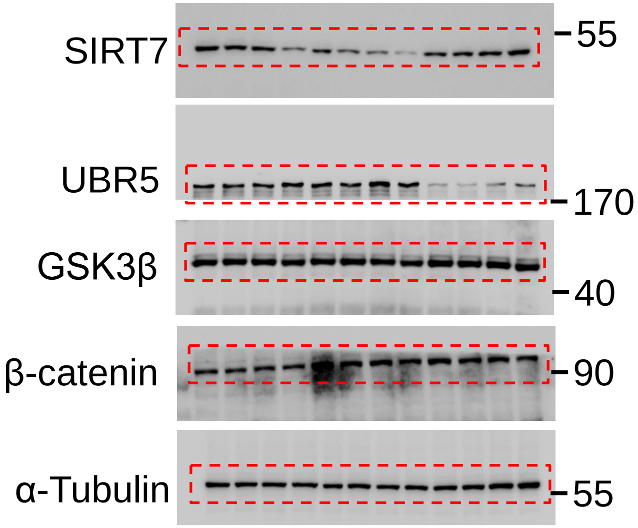

Supplementary Fig. 5d

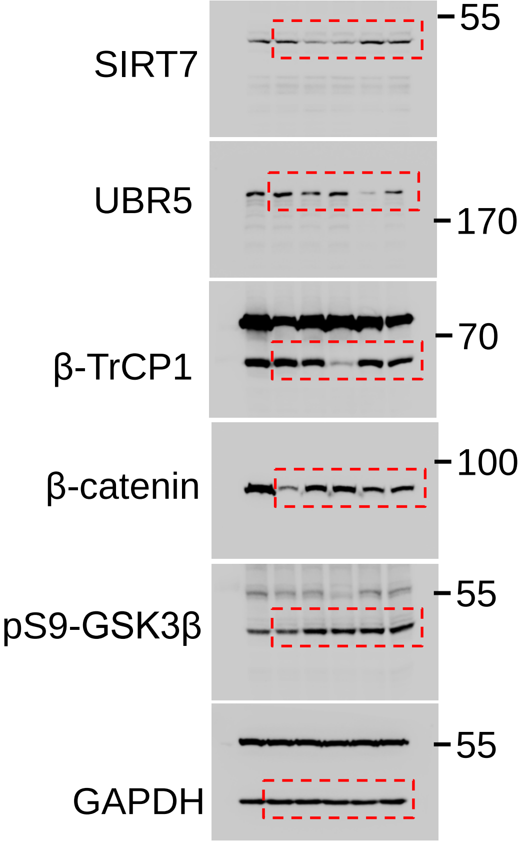

Supplementary Fig. 5

Supplementary Fig. 5i

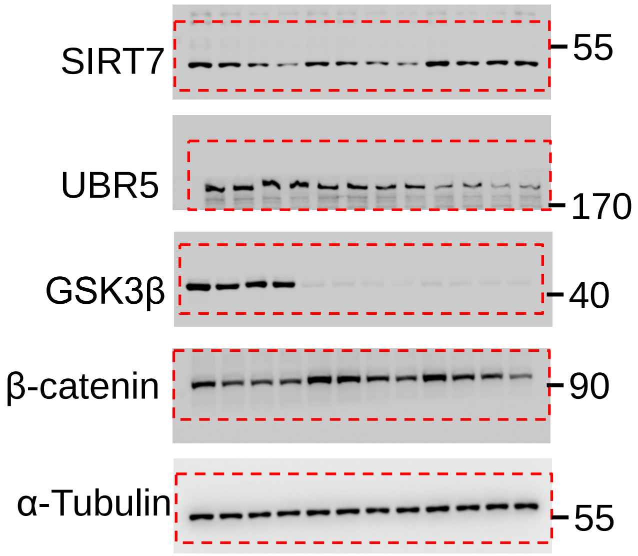

Supplementary Fig. 5k

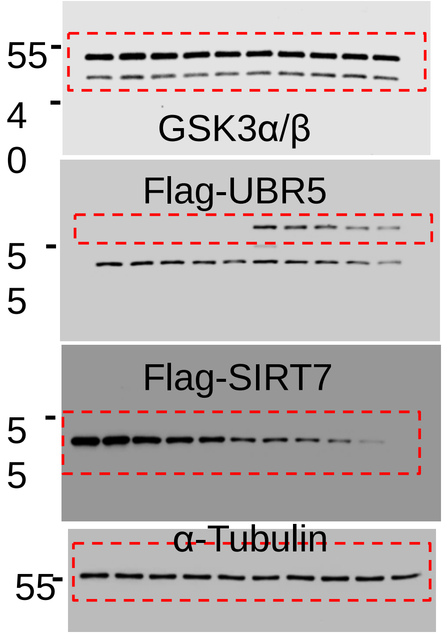

Supplementary Fig. 5l

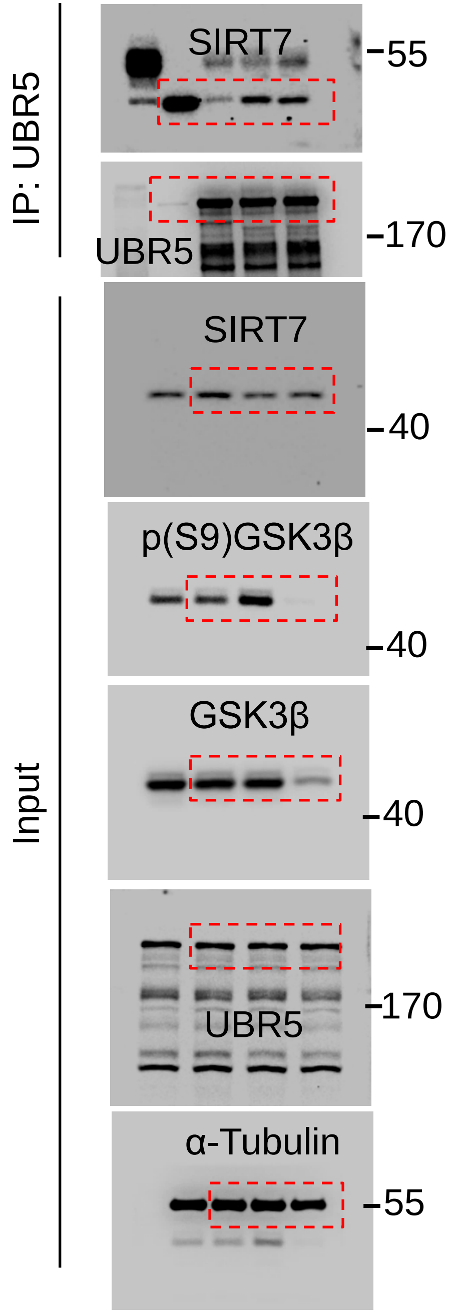

Supplementary Fig. 5j

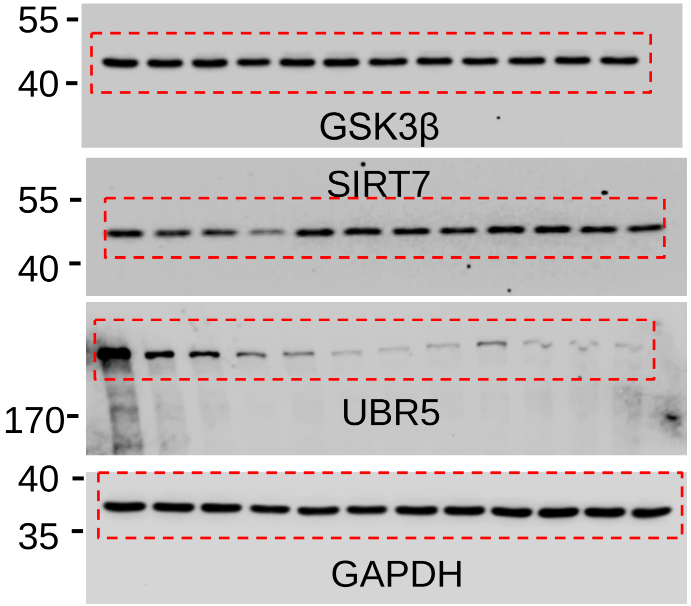

Supplementary Fig. 5m

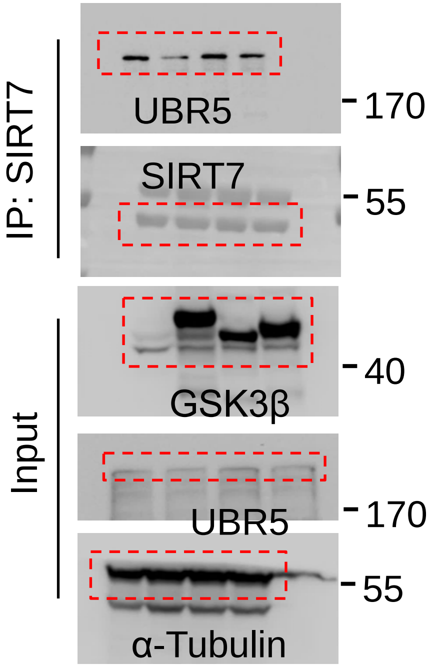

Fig. 4

Fig. 4a

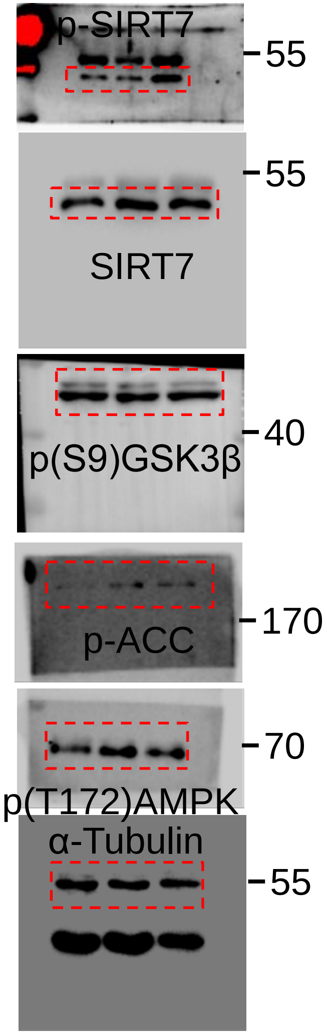

Fig. 4d

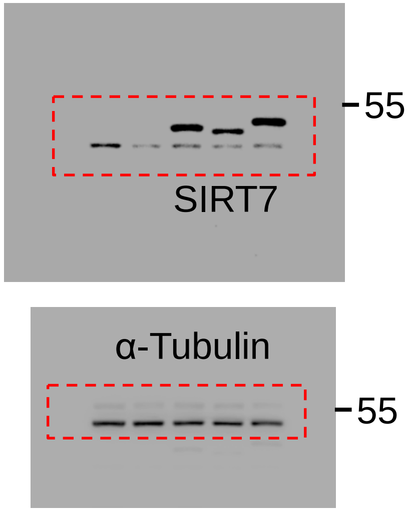

Fig. 4h

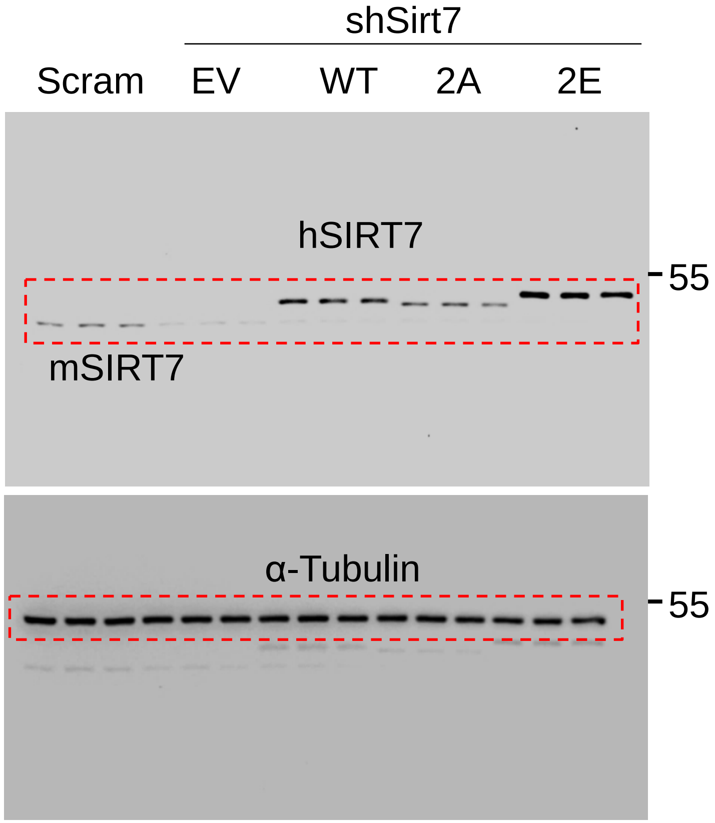

Fig. 5

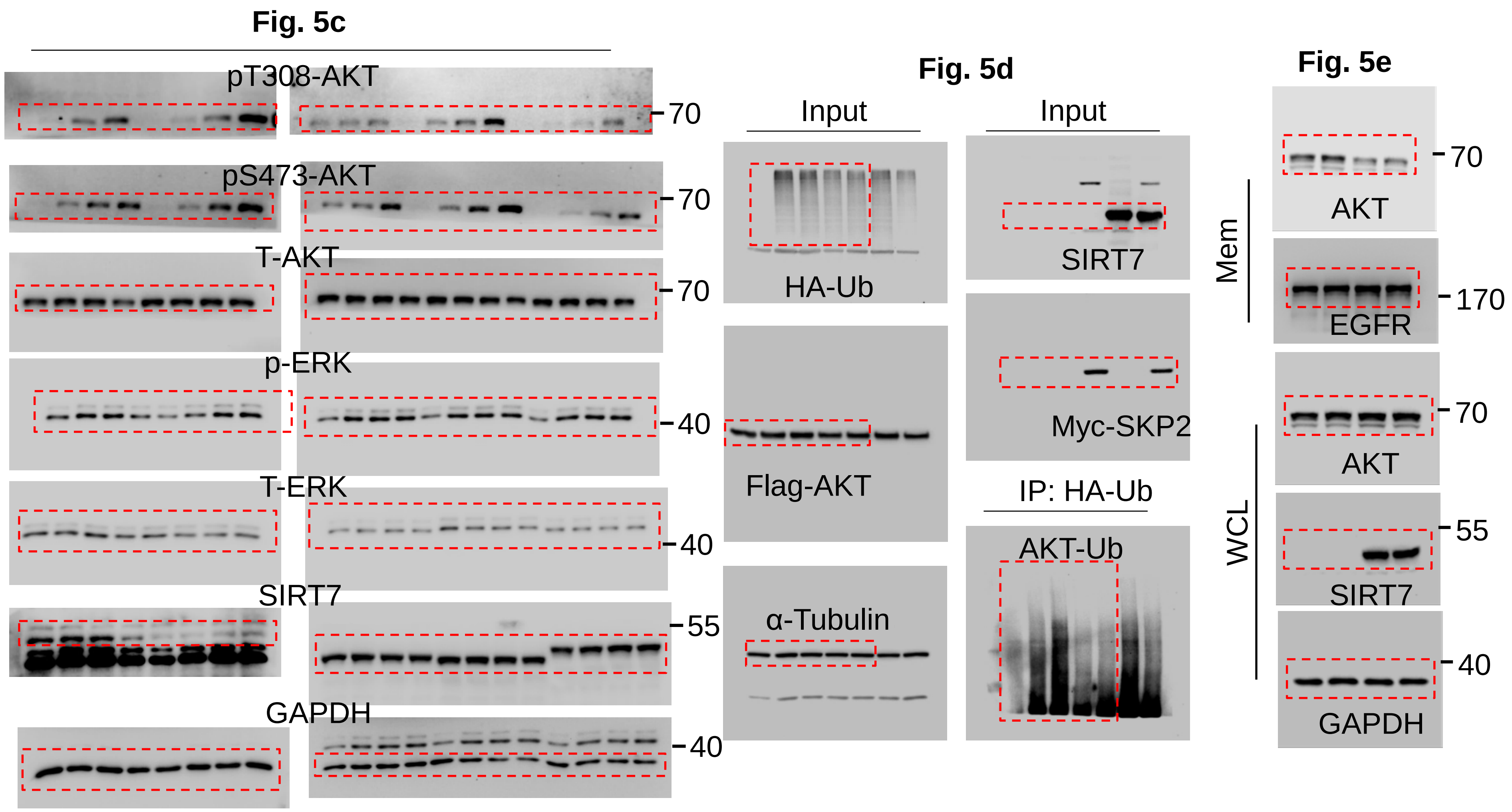
Fig. 5c
pT308-AKT
70
pS473-AKT
70
T-AKT
70
p-ERK
40
T-ERK
40
SIRT7
55
GAPDH
40
Fig. 5d
Input
HA-Ub
Flag-AKT
α-Tubulin
Input
SIRT7
Myc-SKP2
IP: HA-Ub
AKT-Ub
Fig. 5e
Mem
AKT
70
EGFR
170
WCL
AKT
70
SIRT7
55
GAPDH
40

Fig. 5

Fig. 5f

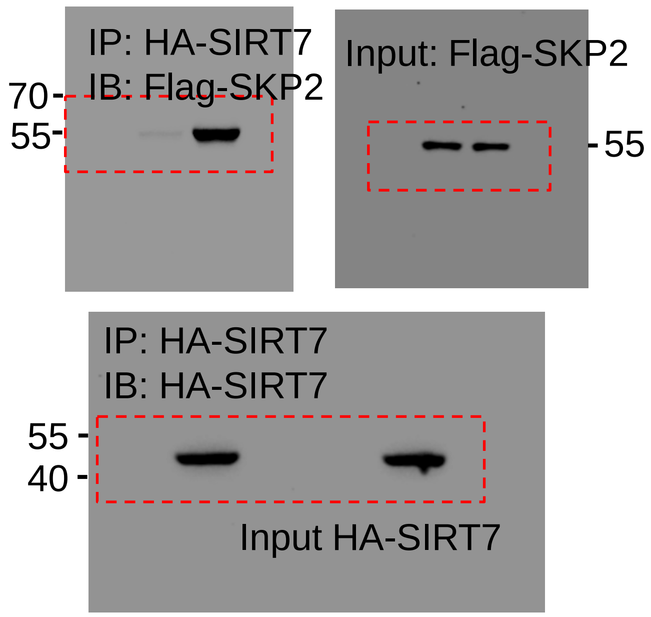

Fig. 5g

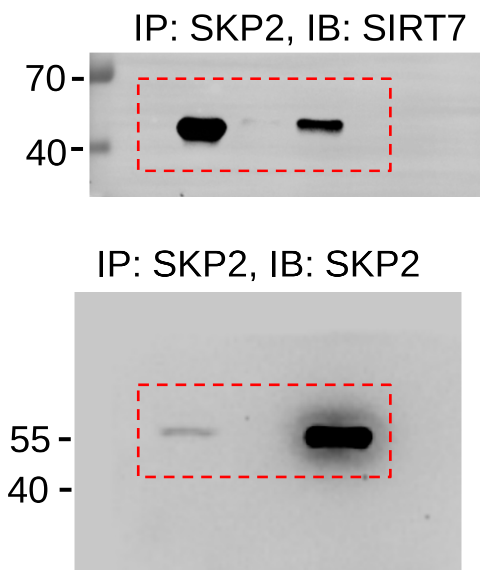

Fig. 5k

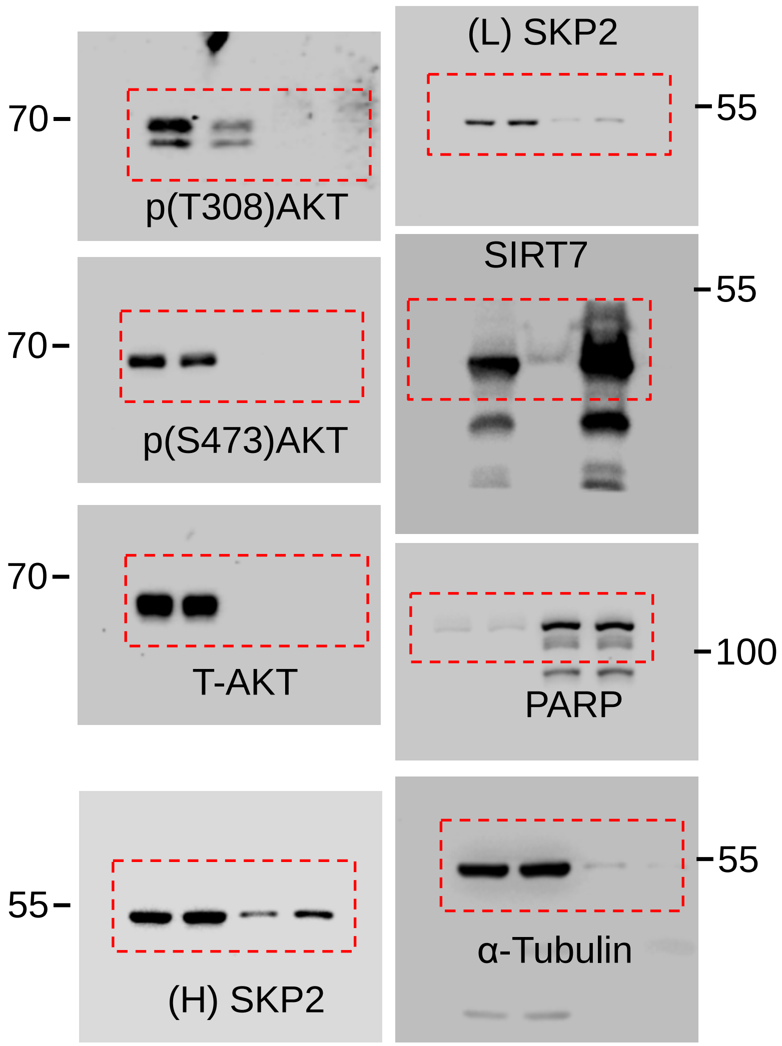

Fig. 5

Fig. 5i

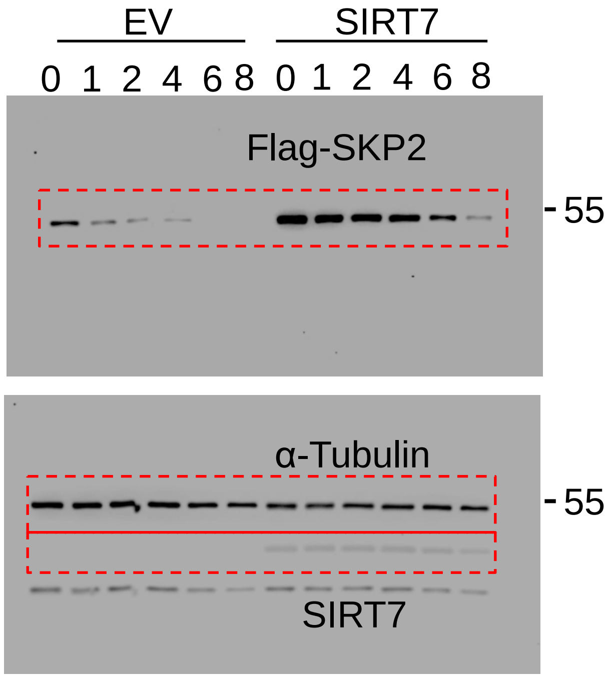

Fig. 5h

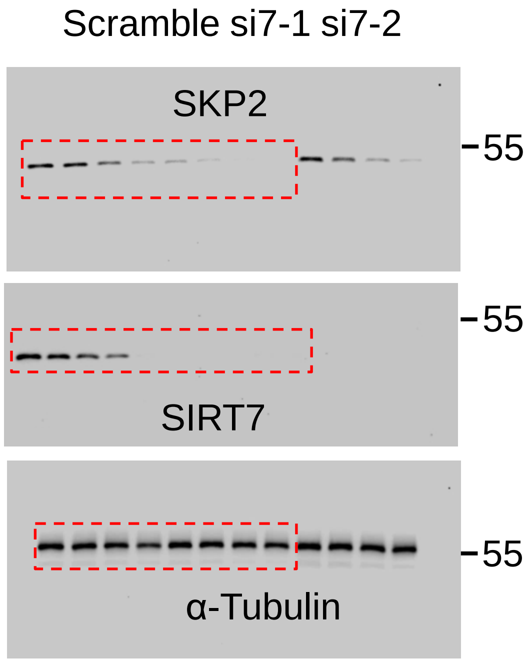

Fig. 5j

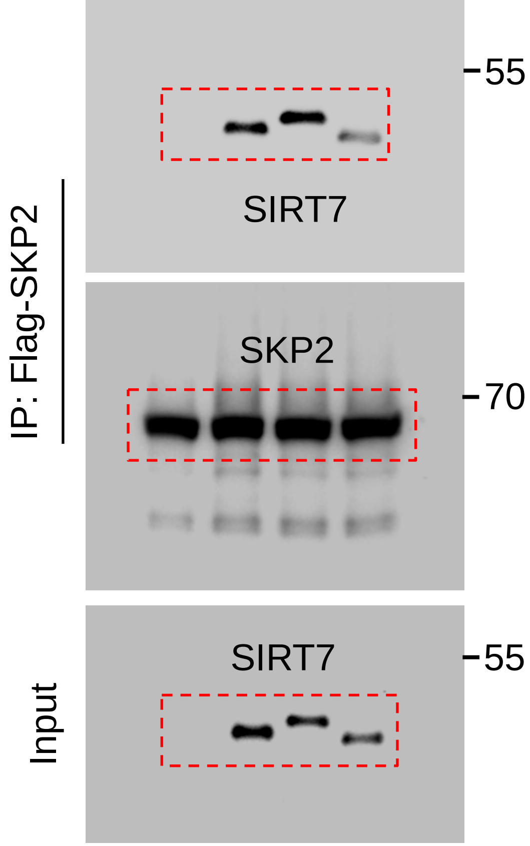

Fig. 5

Fig. 5l

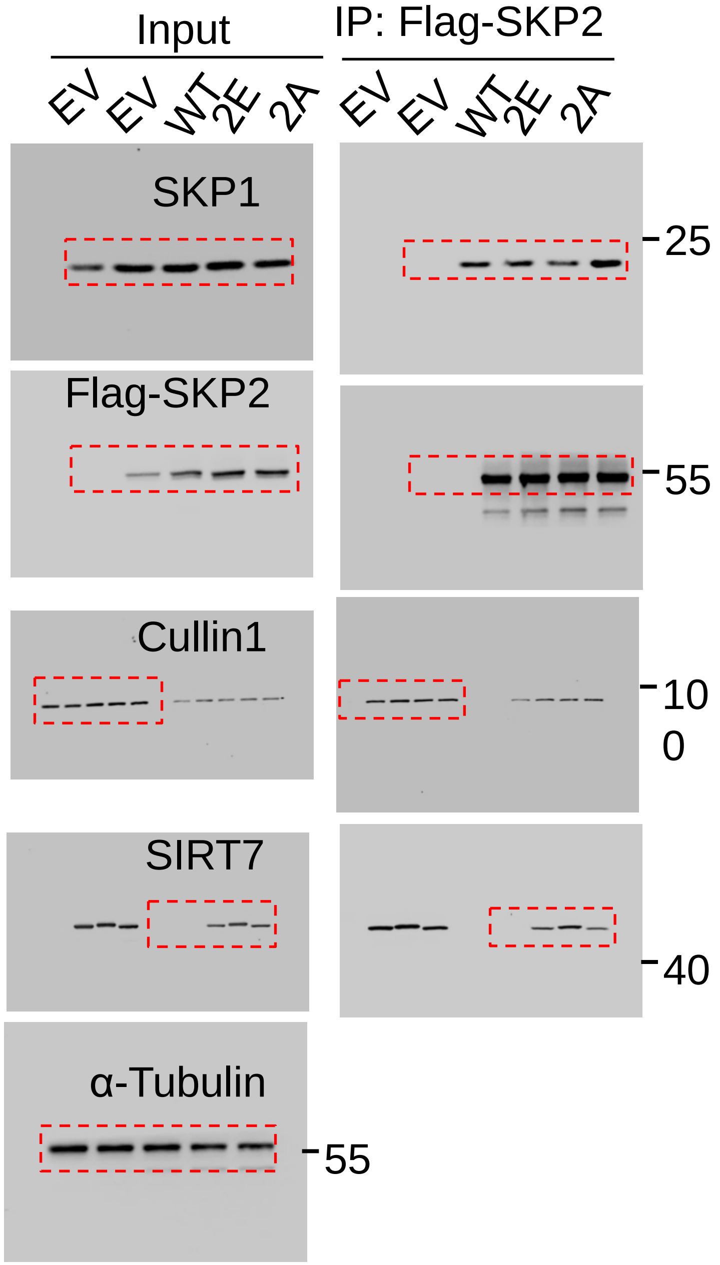

Fig. 5m

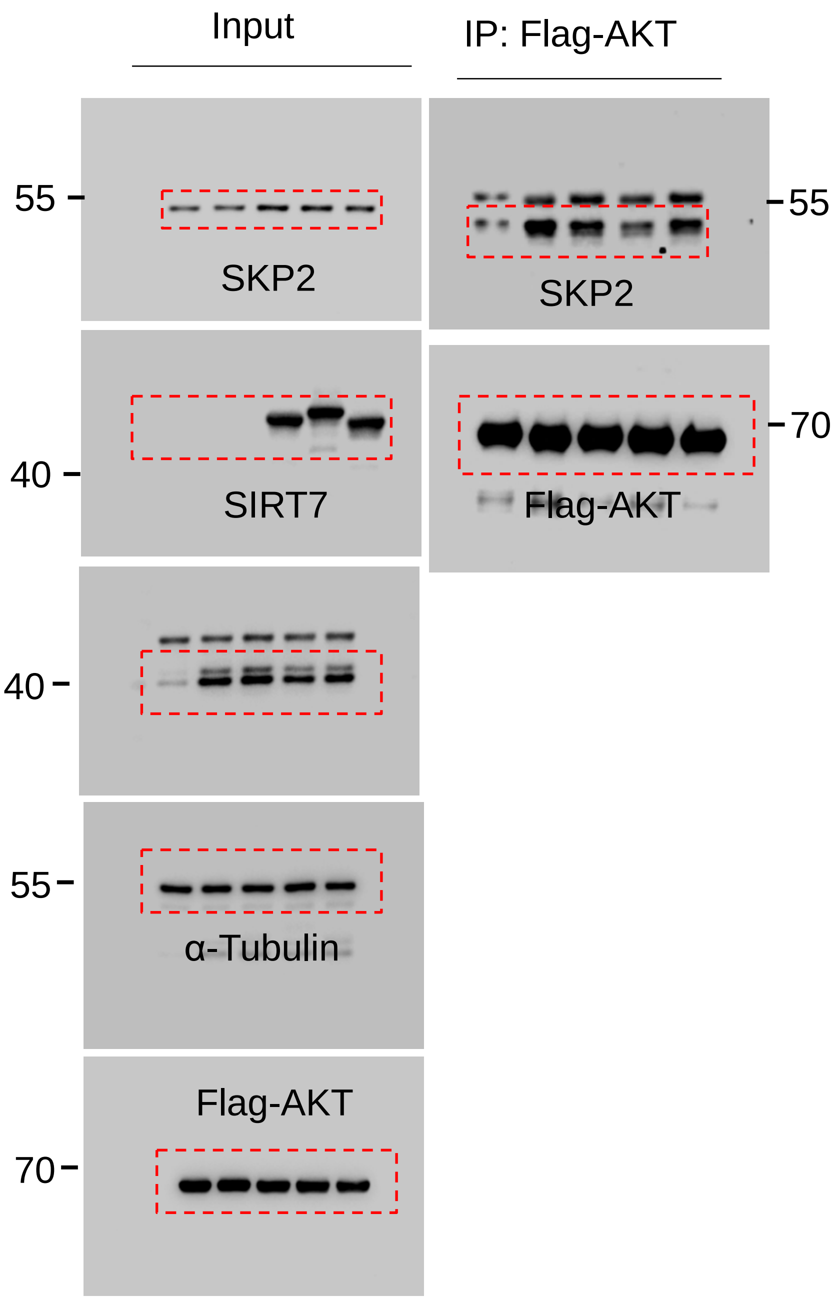

Fig. 5

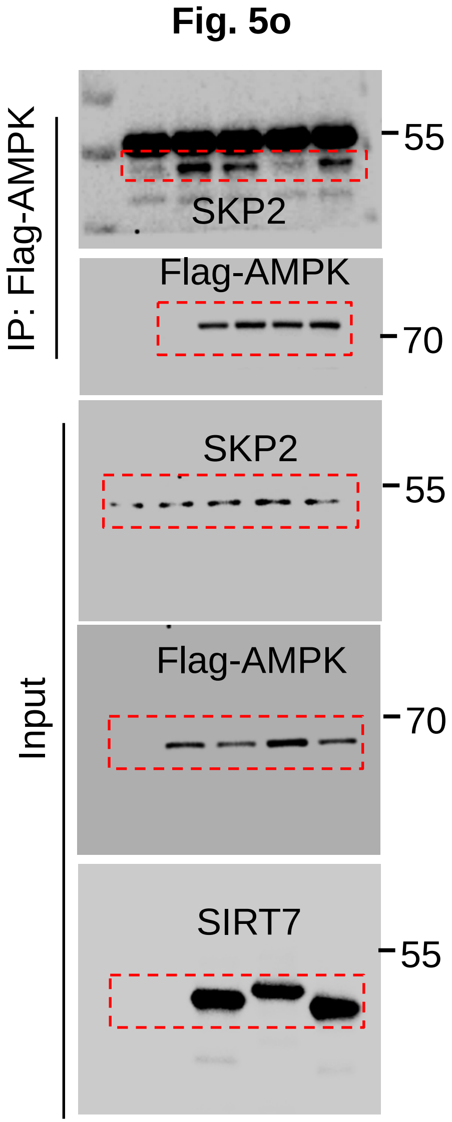

Fig. 5o
IP: Flag-AMPK
SKP2
55
Flag-AMPK
70
Input
SKP2
55
Flag-AMPK
70
SIRT7
55

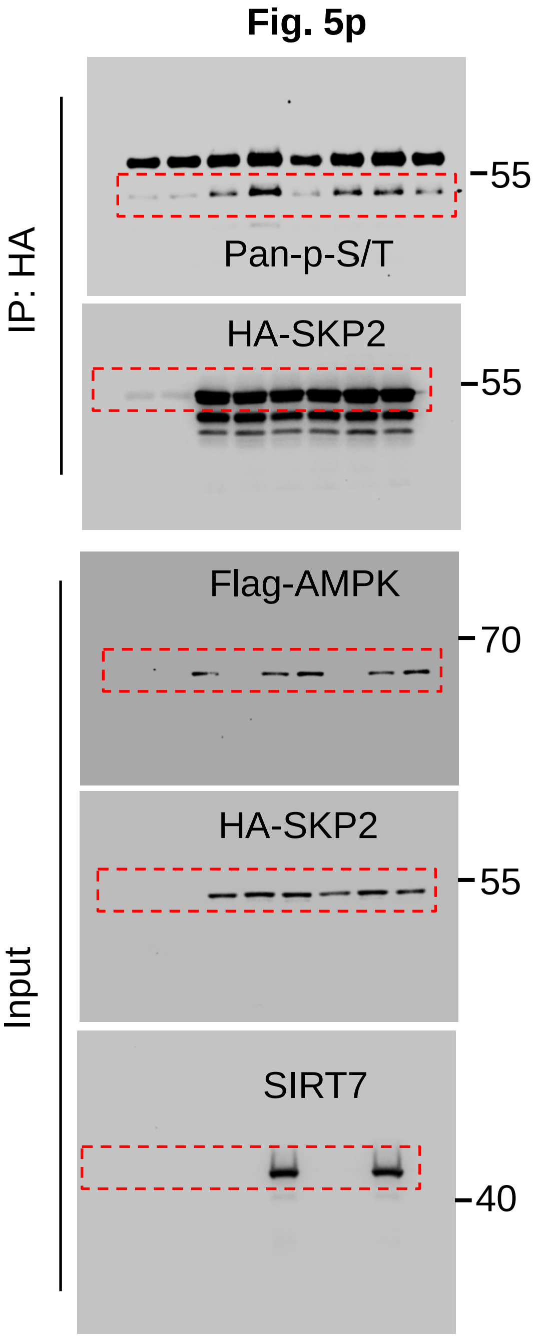

Fig. 5p
IP: HA
55
Pan-p-S/T
HA-SKP2
55
Input
Flag-AMPK
70
HA-SKP2
55
SIRT7
40

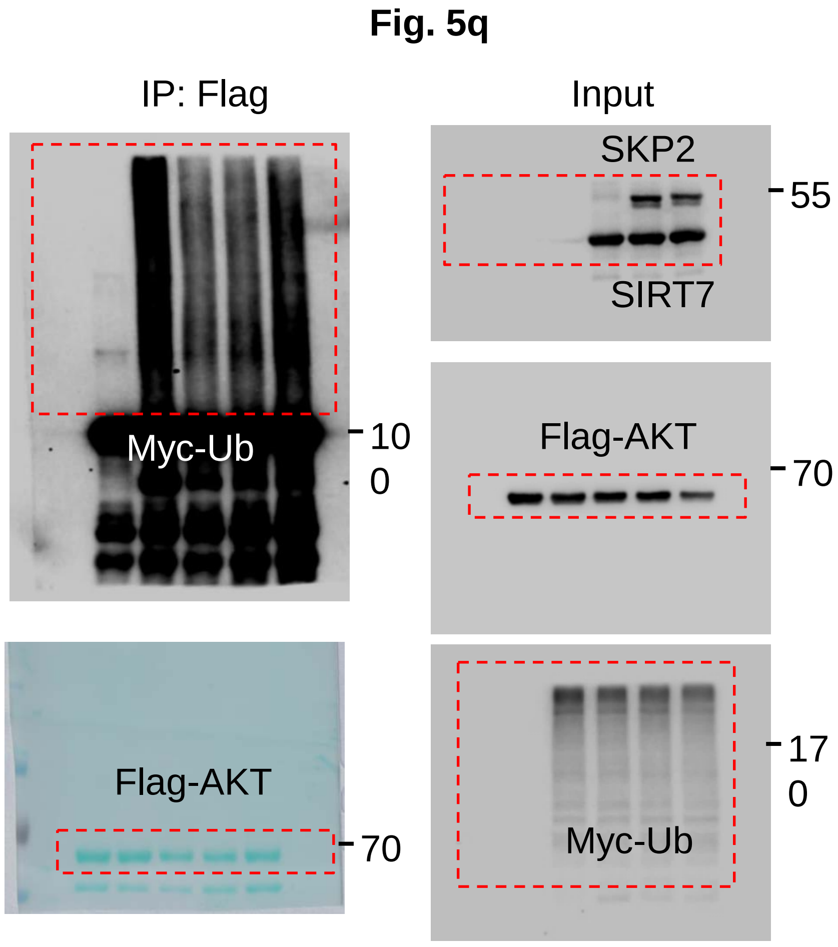

Fig. 5q
IP: Flag
Myc-Ub
10
0
Flag-AKT
70
Input
SKP2
55
SIRT7
Flag-AKT
70
17
0
Myc-Ub

Supplementary Fig. 6

Supplementary Fig. 6a

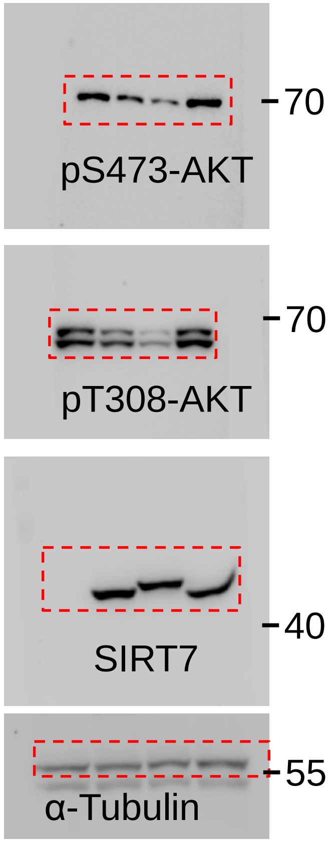

Supplementary Fig. 6b

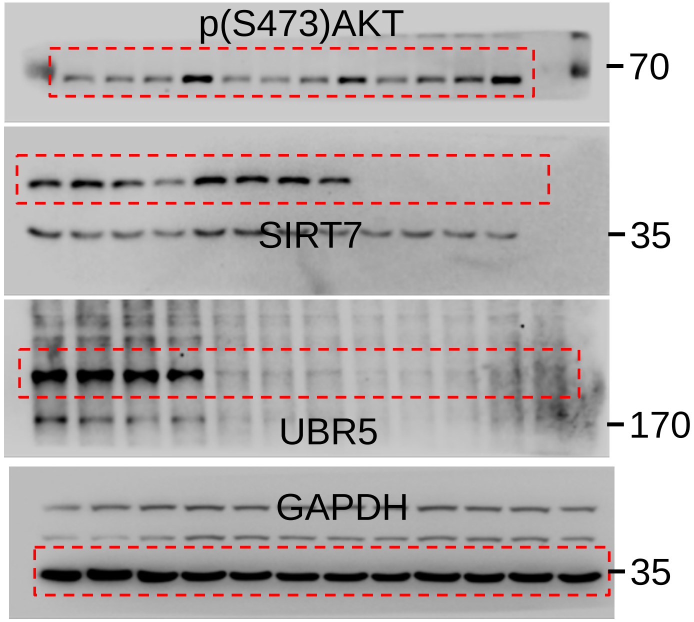

Supplementary Fig. 6c

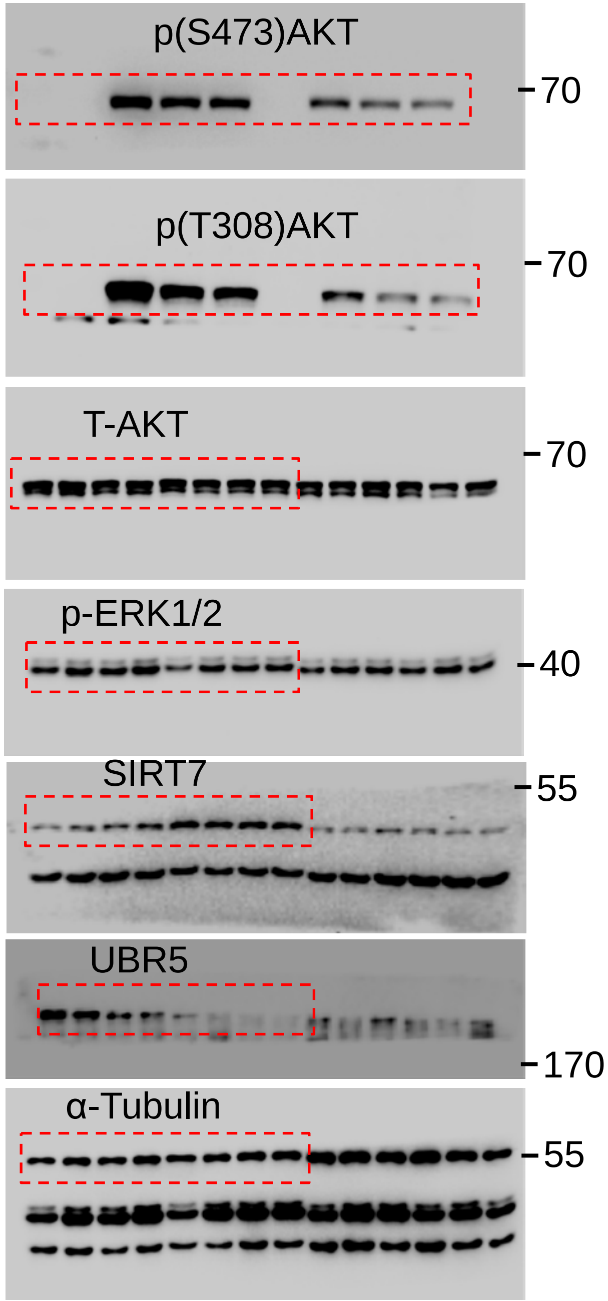

Supplementary Fig. 6

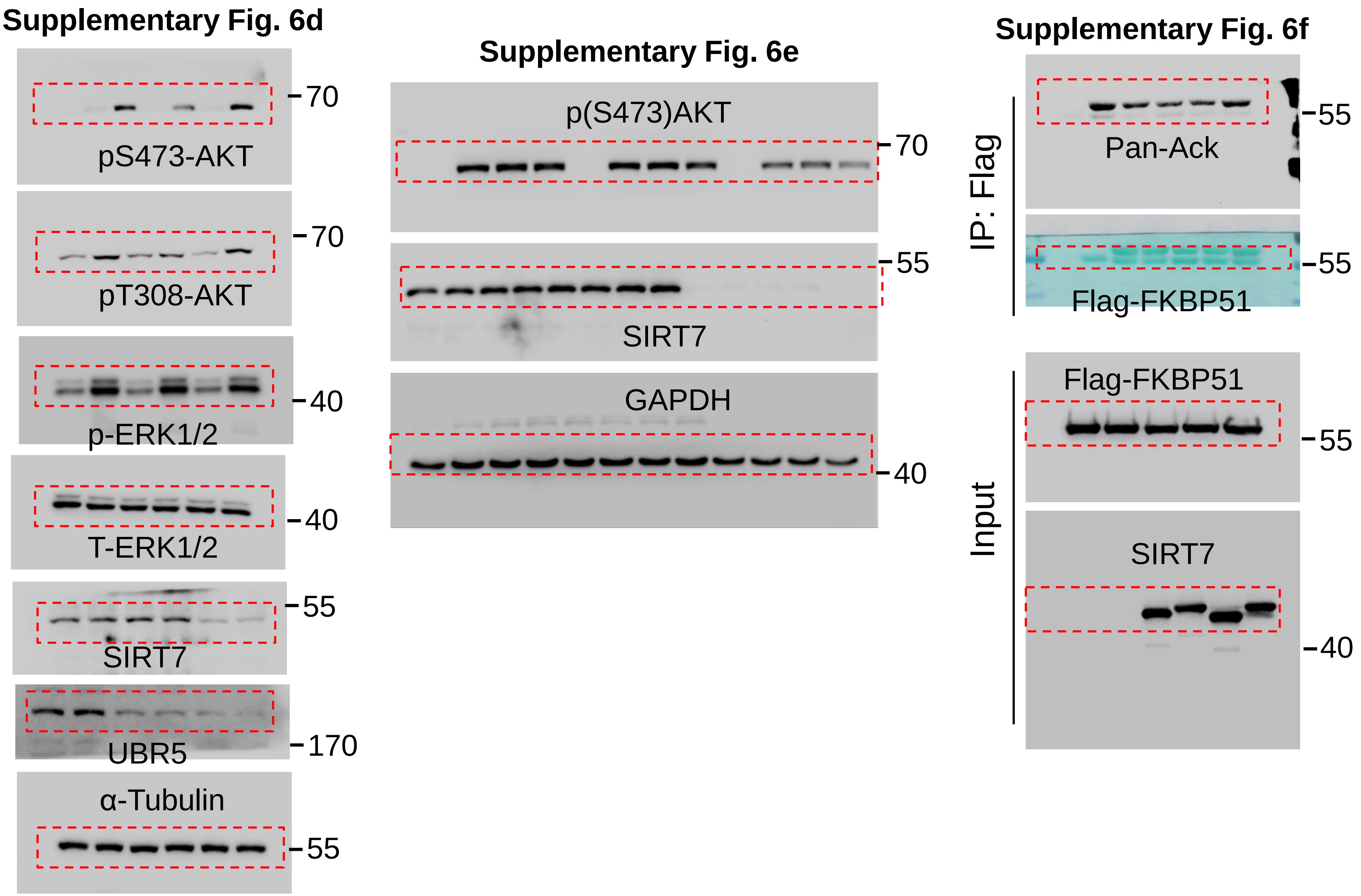

Supplementary Fig. 6

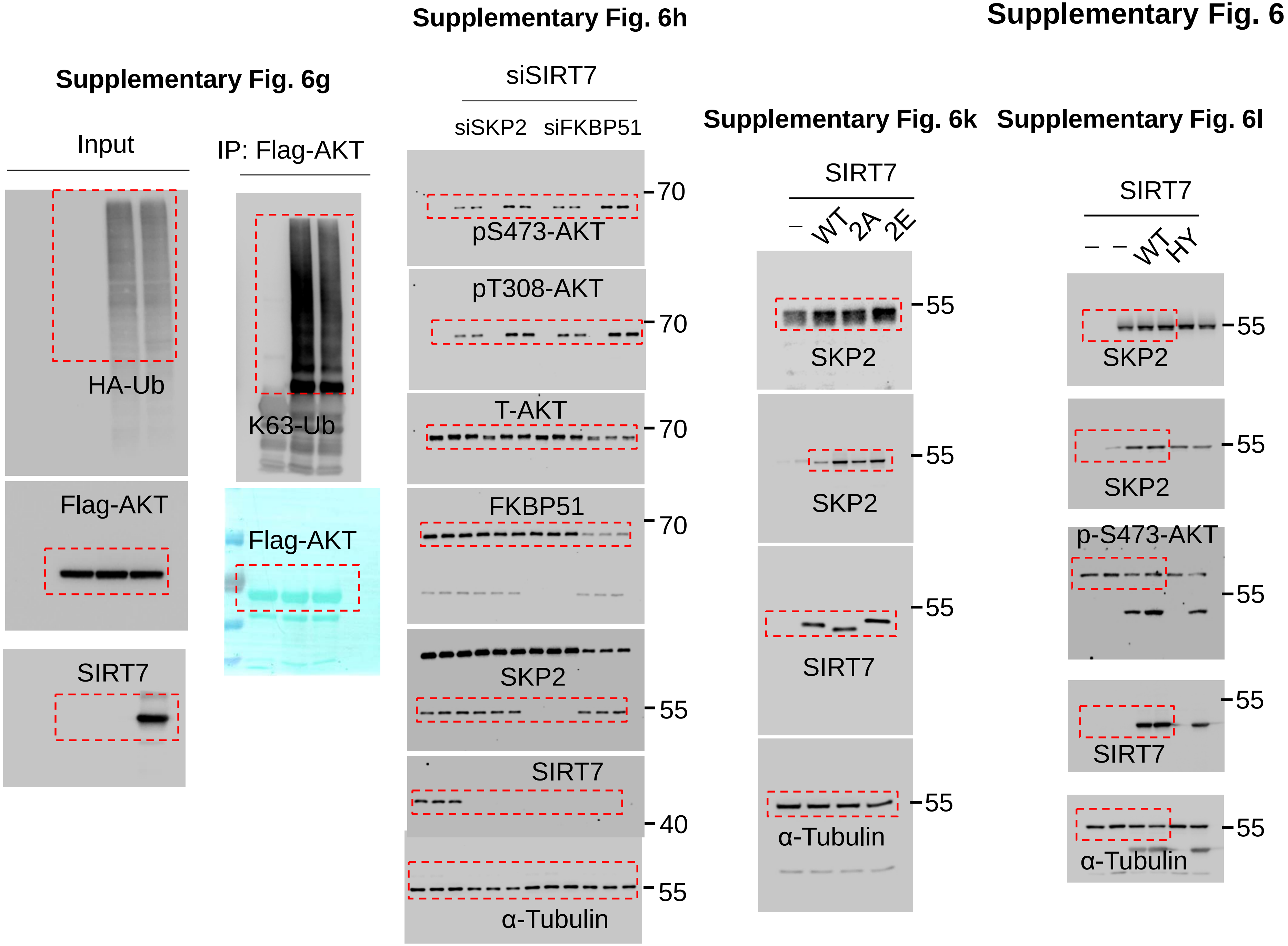

Supplementary Fig. 6m

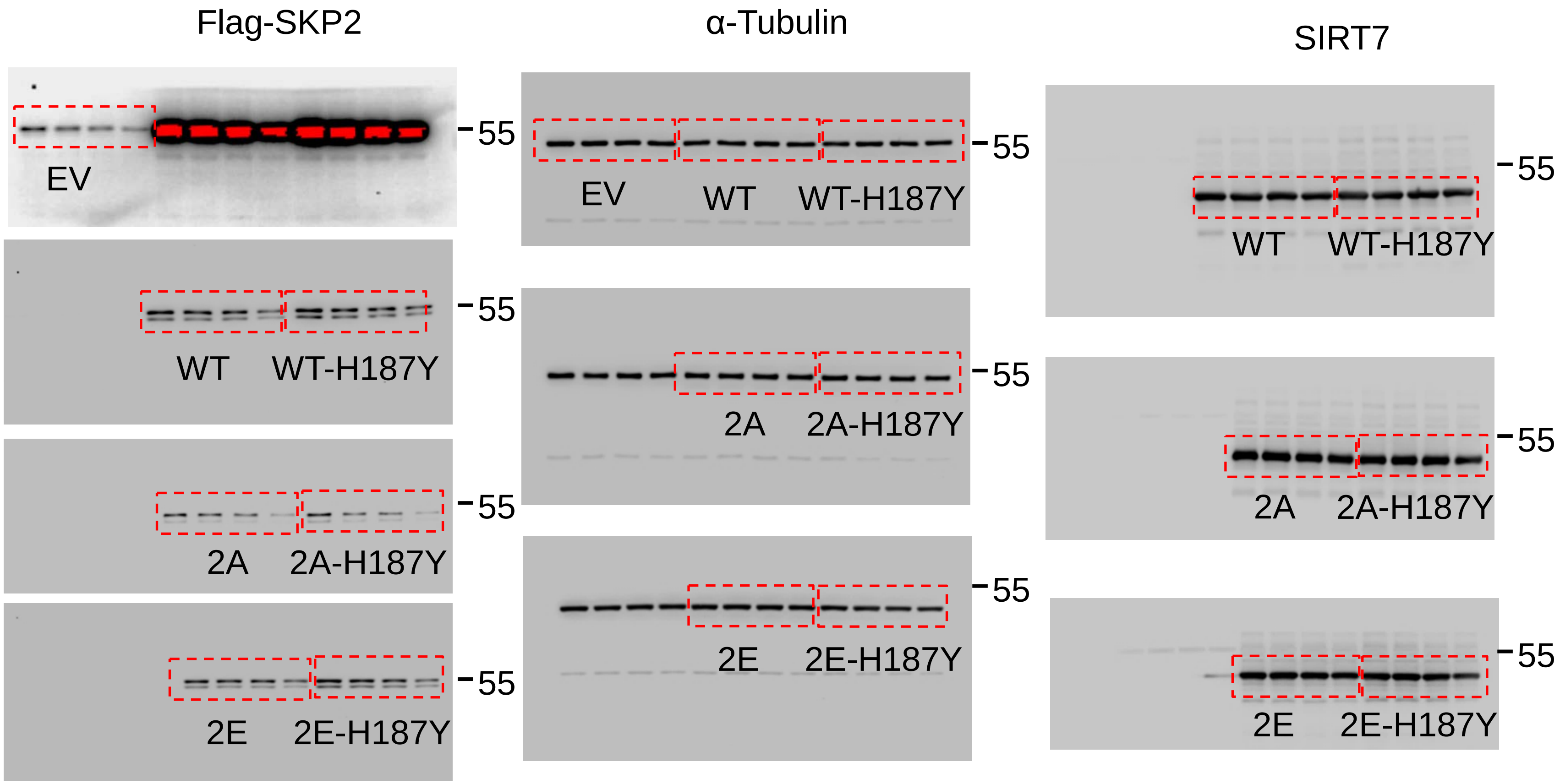

Supplementary Fig. 6n

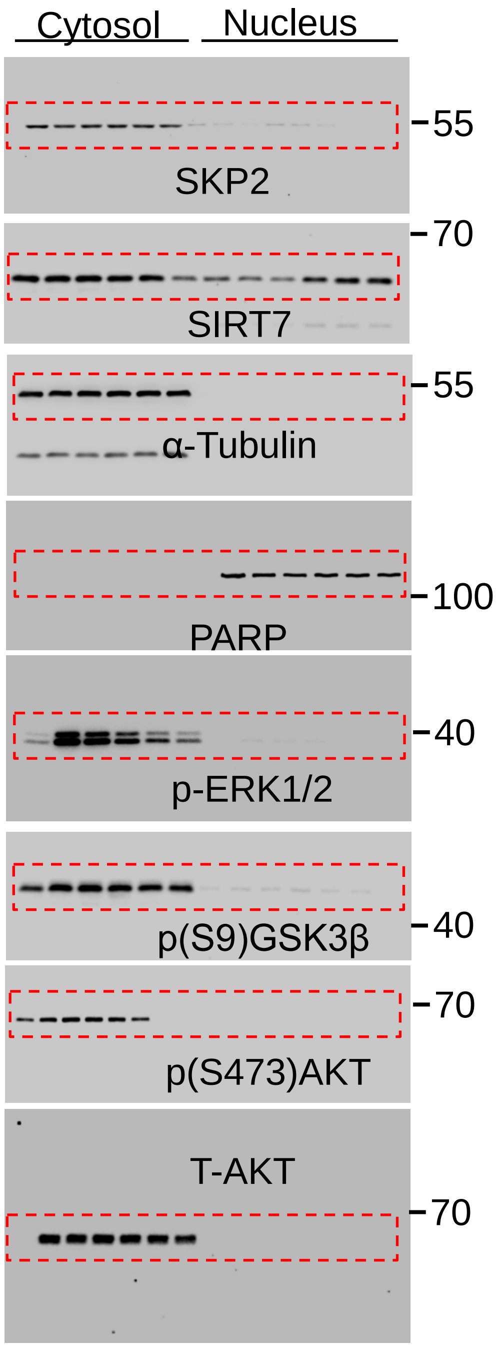

Supplementary Fig. 6p

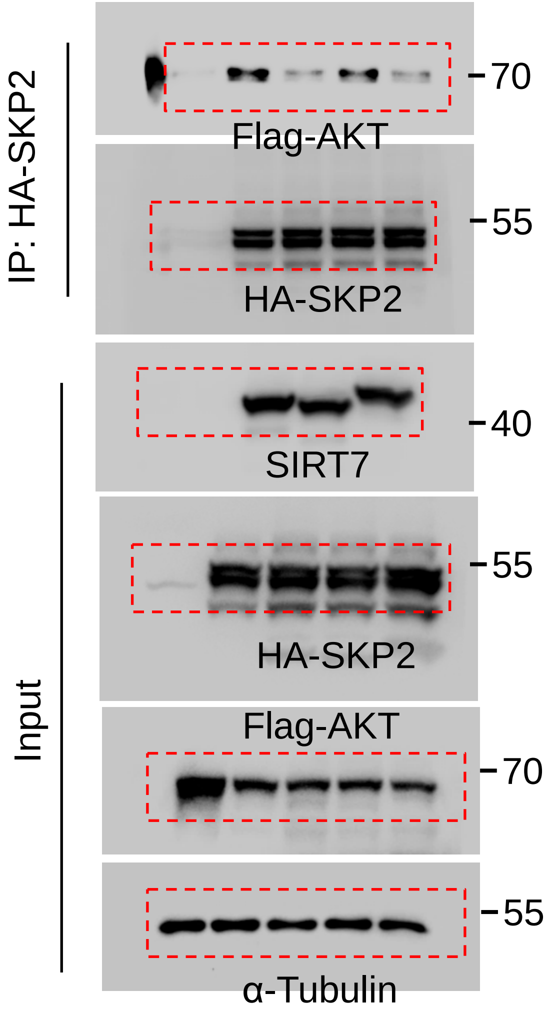

Supplementary Fig. 7

Supplementary Fig. 7a

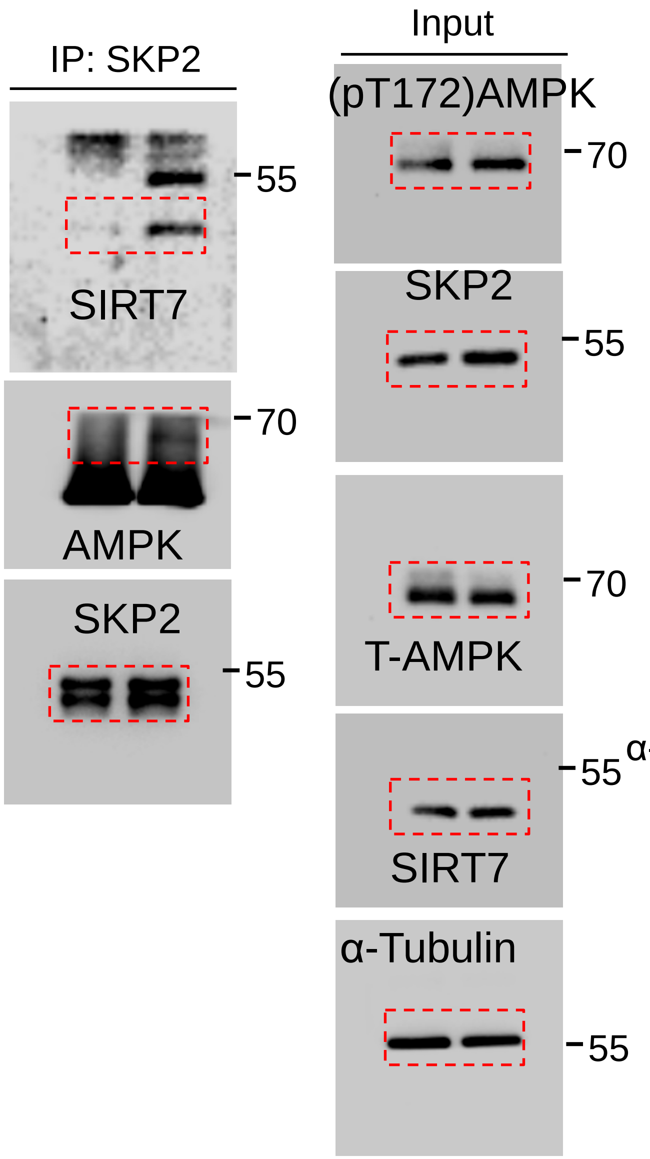

Supplementary Fig. 7b

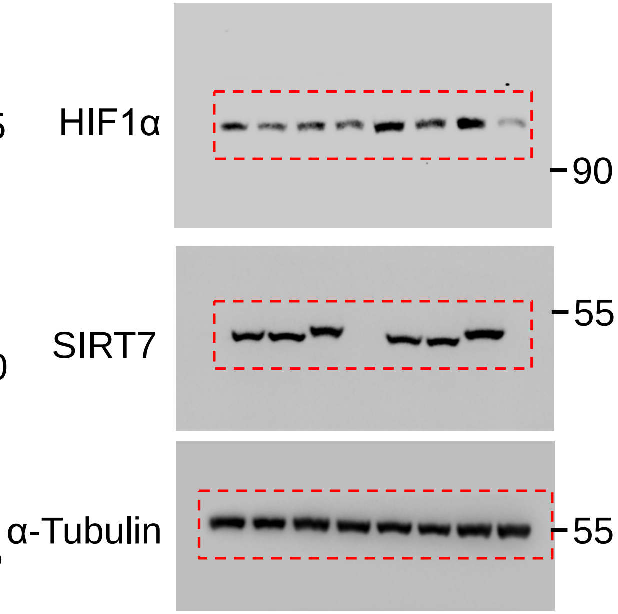

Supplementary Fig. 7c

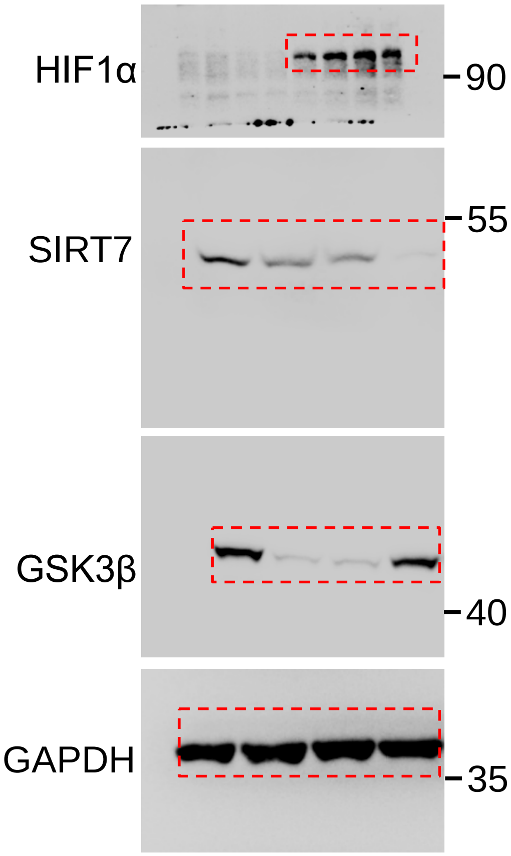

Fig. 6

Fig. 6k

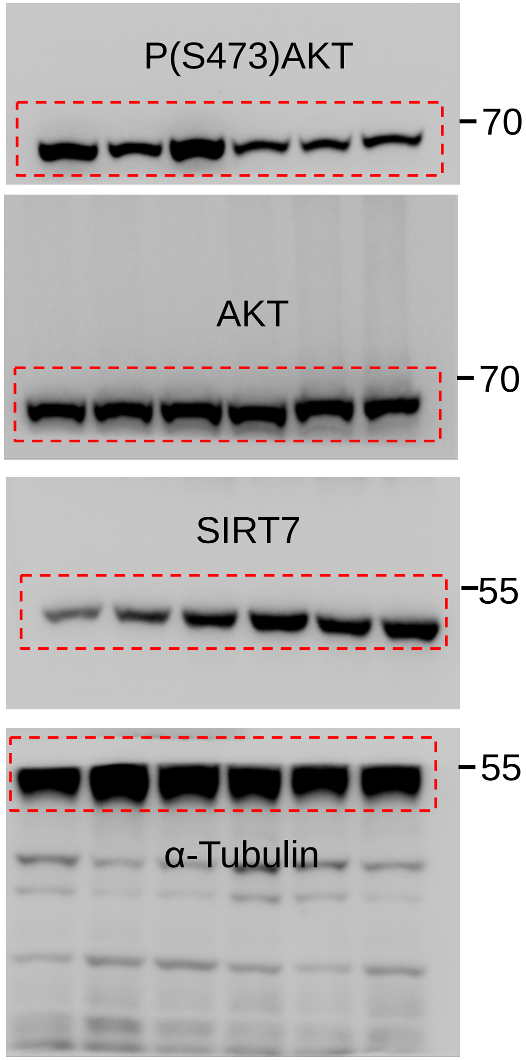

Supplementary Fig. 8

Supplementary Fig. 8a upper

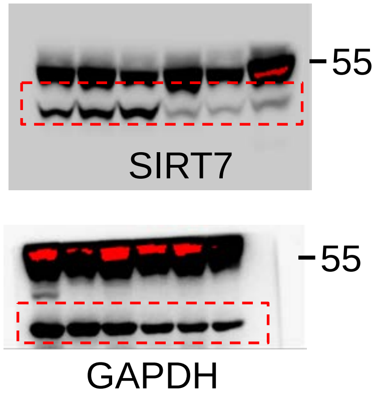

Supplementary Fig. 8a lower

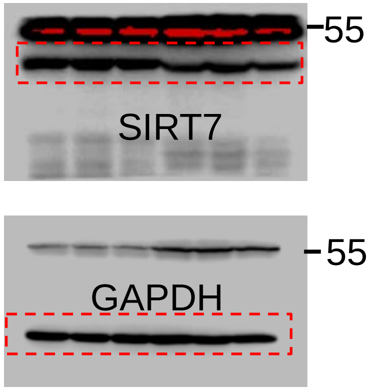

Supplementary Fig. 8d

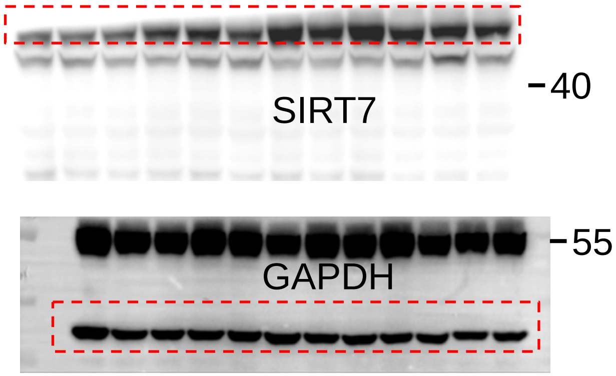

Fig. 7

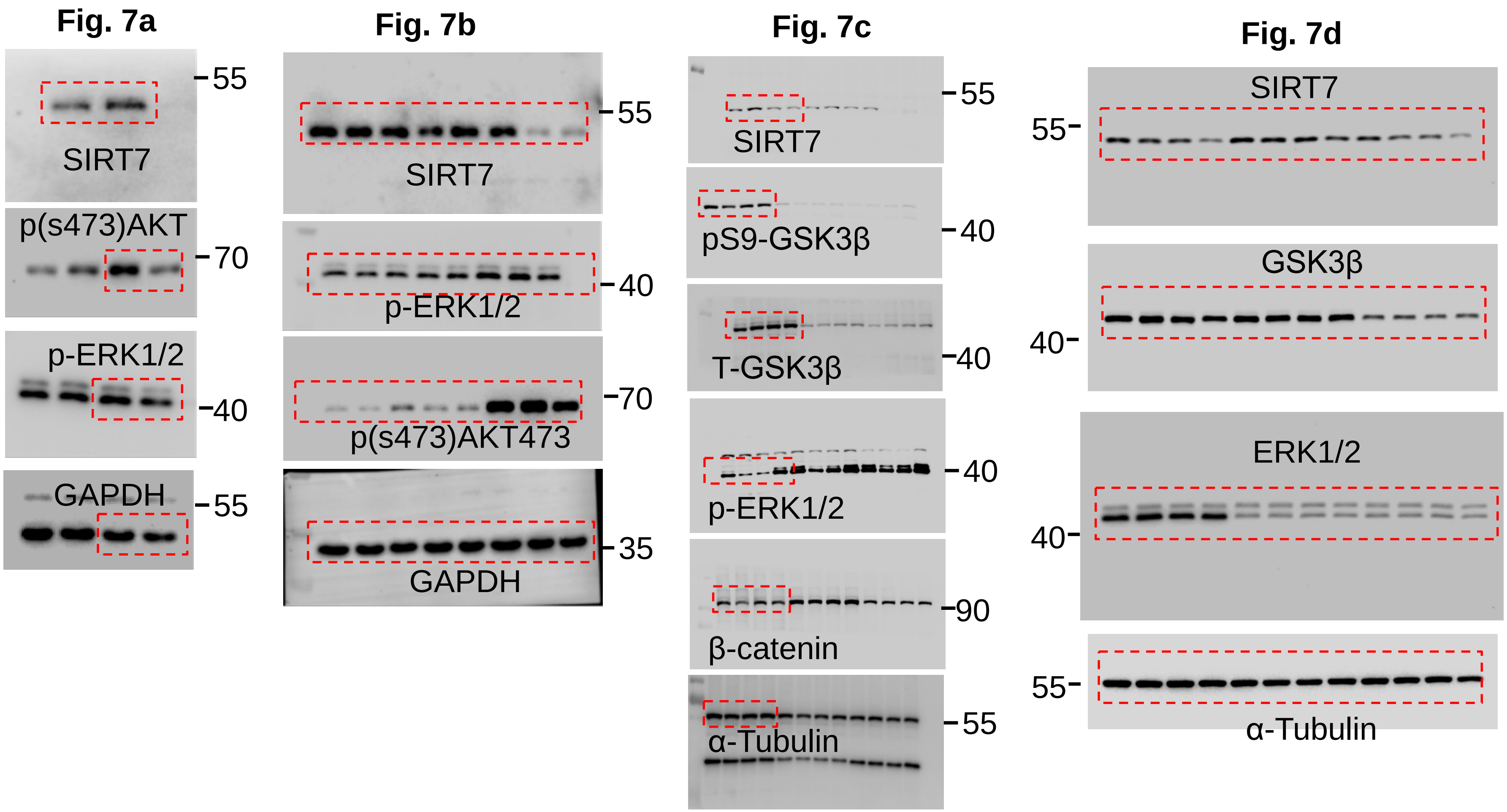

Fig. 7a
SIRT7
55
p(s473)AKT
70
p-ERK1/2
40
GAPDH
55
Fig. 7b
SIRT7
55
p-ERK1/2
40
p(s473)AKT473
70
GAPDH
35
Fig. 7c
SIRT7
55
pS9-GSK3β
40
T-GSK3β
40
p-ERK1/2
40
β-catenin
90
α-Tubulin
55
Fig. 7d
SIRT7
55
GSK3β
40
ERK1/2
40
55
α-Tubulin

Fig. 7

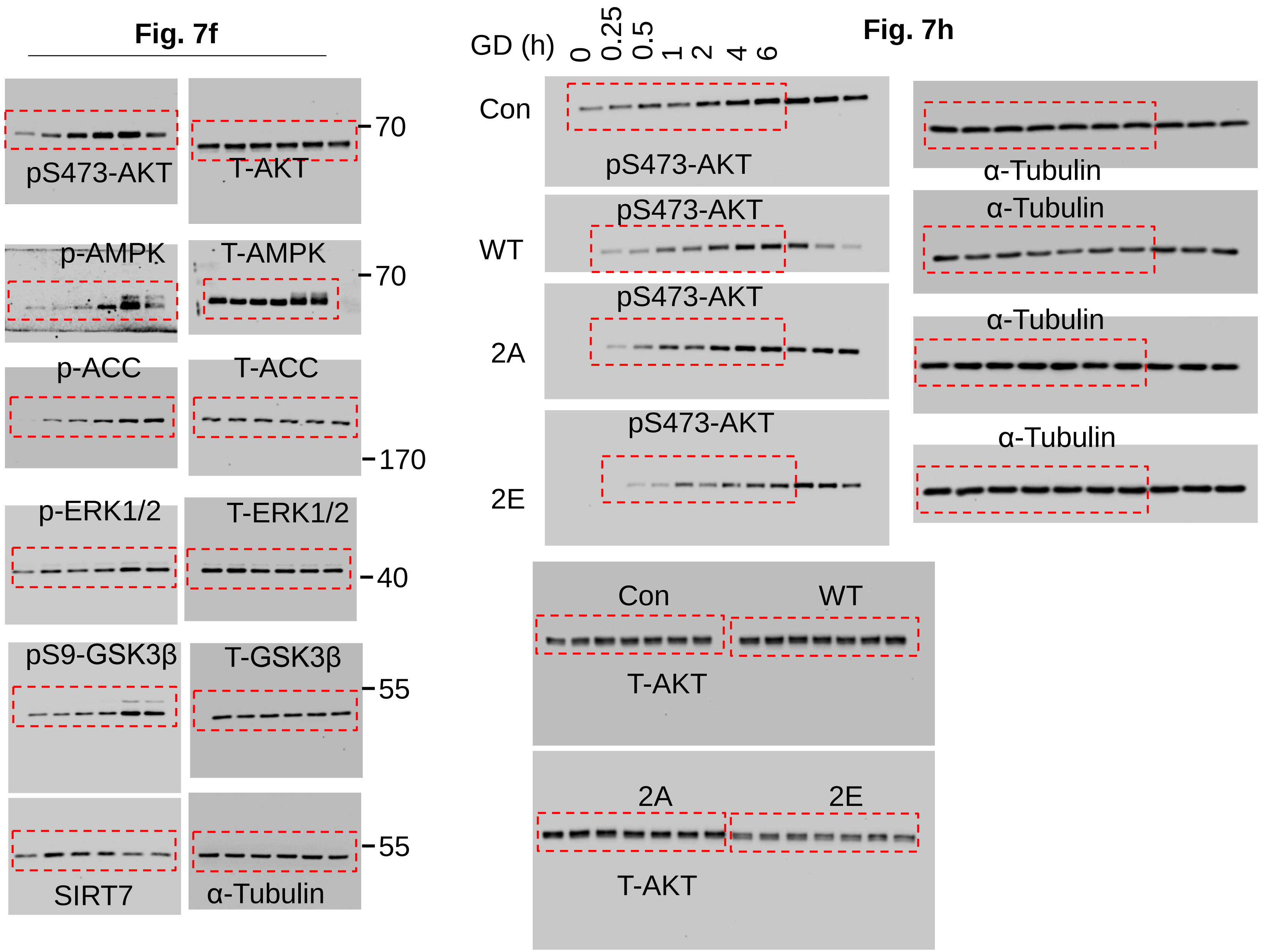
Fig. 7f
pS473-AKT
T-AKT
70
p-AMPK
T-AMPK
70
p-ACC
T-ACC
170
p-ERK1/2
T-ERK1/2
40
pS9-GSK3β
T-GSK3β
55
SIRT7
α-Tubulin
55
Fig. 7h
GD (h)
0
0.25
0.5
1
2
4
6
Con
WT
2A
2E
pS473-AKT
α-Tubulin
T-AKT

Fig. 7

Fig. 7i

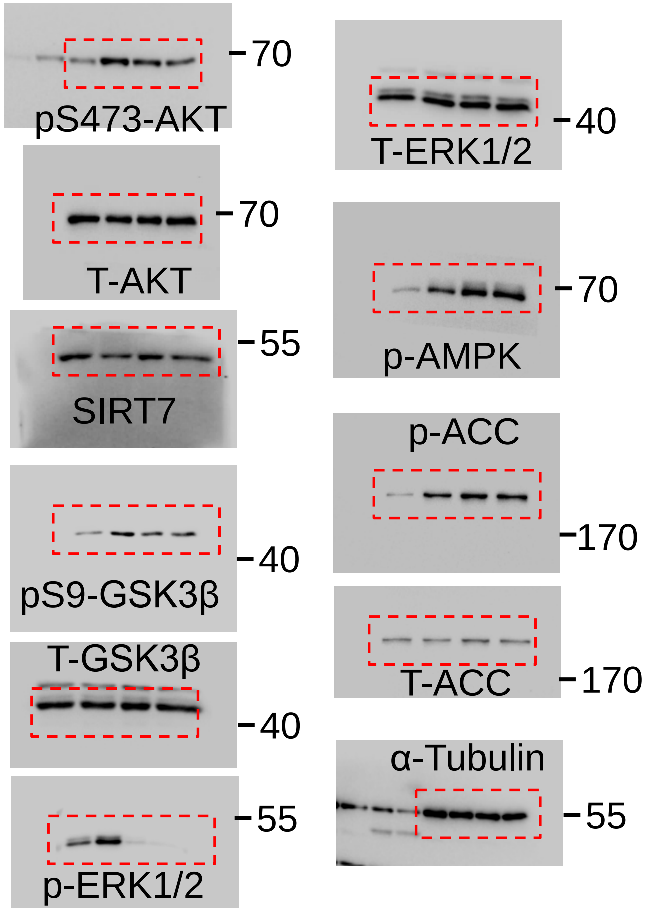

Fig. 7j

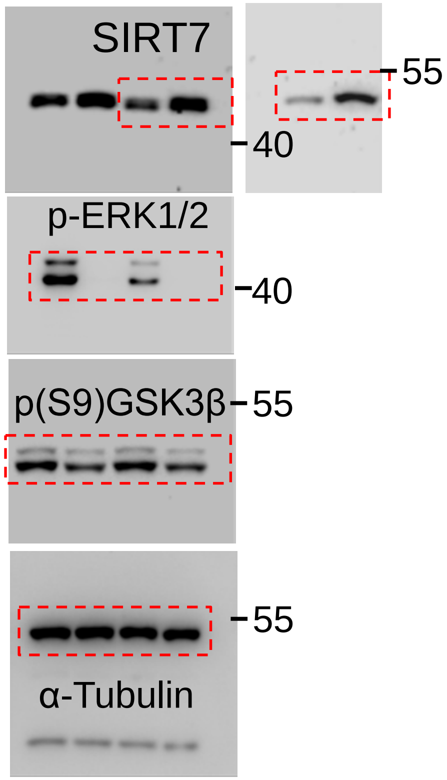

Supplementary Fig. 11

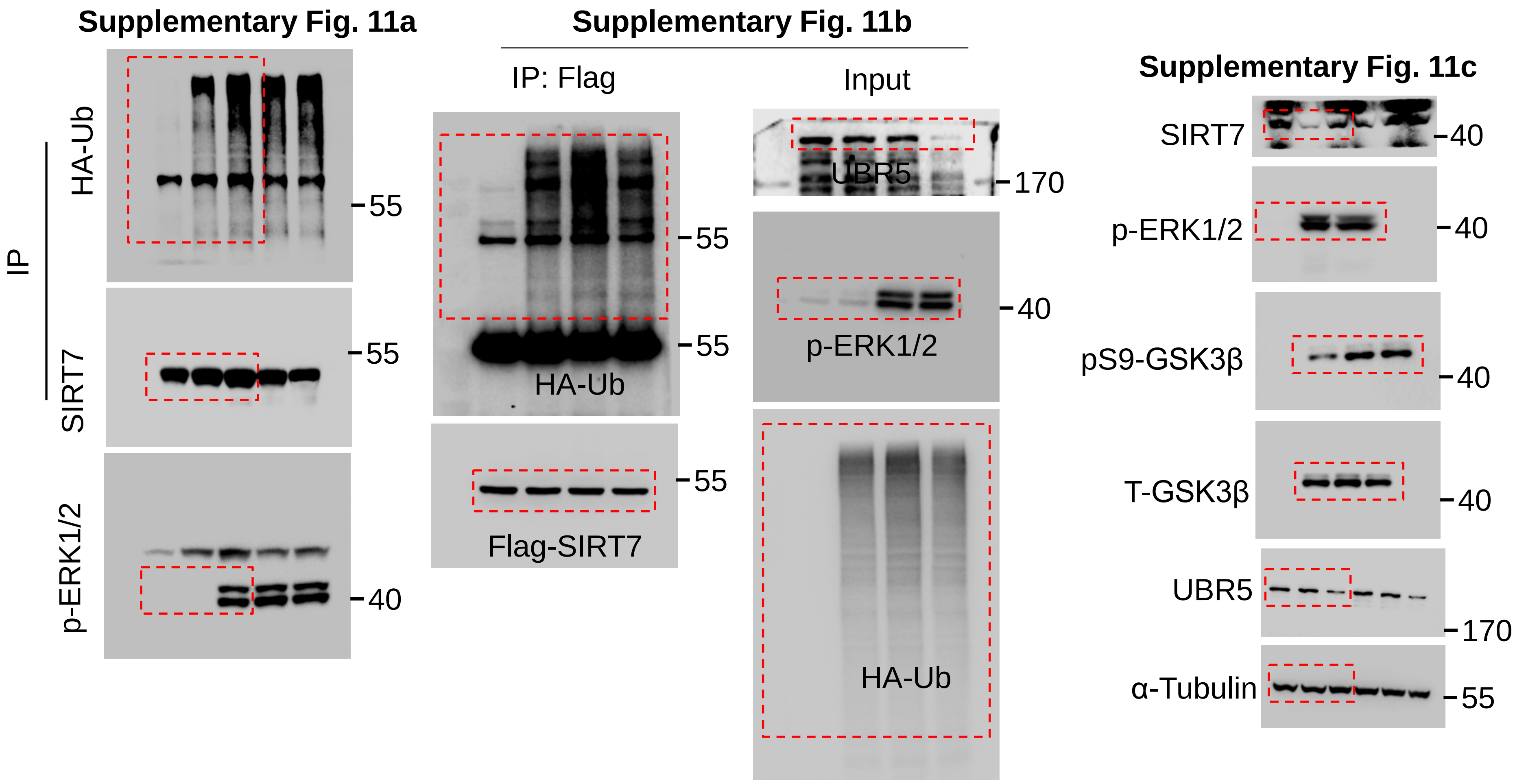

Supplementary Fig. 11

Supplementary Fig. 11d

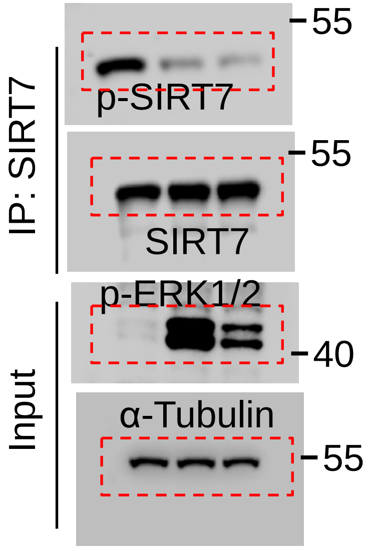

Supplementary Fig. 11e

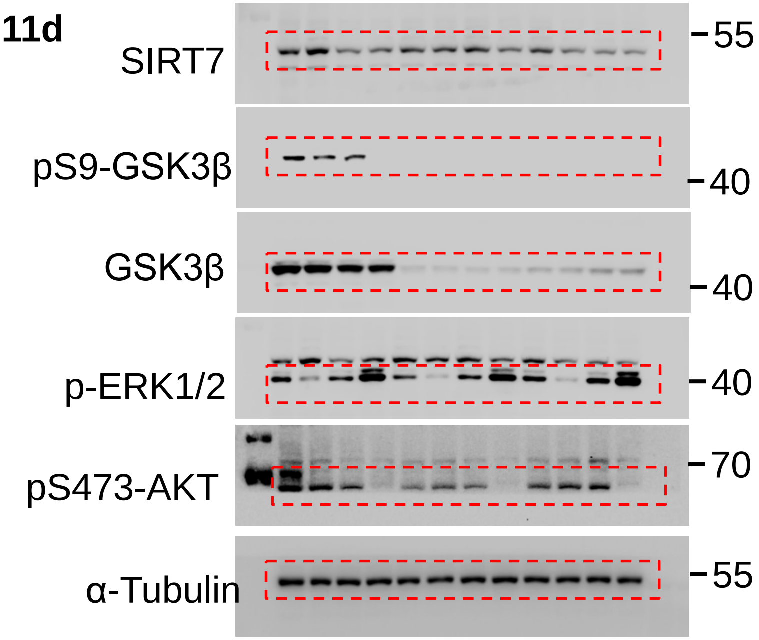

Supplementary Fig. 11f

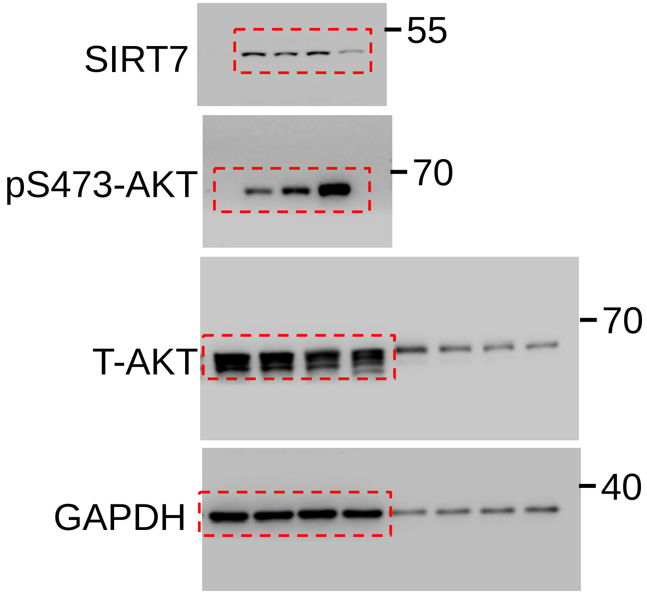

Supplementary Fig. 11

Supplementary Fig. 11i

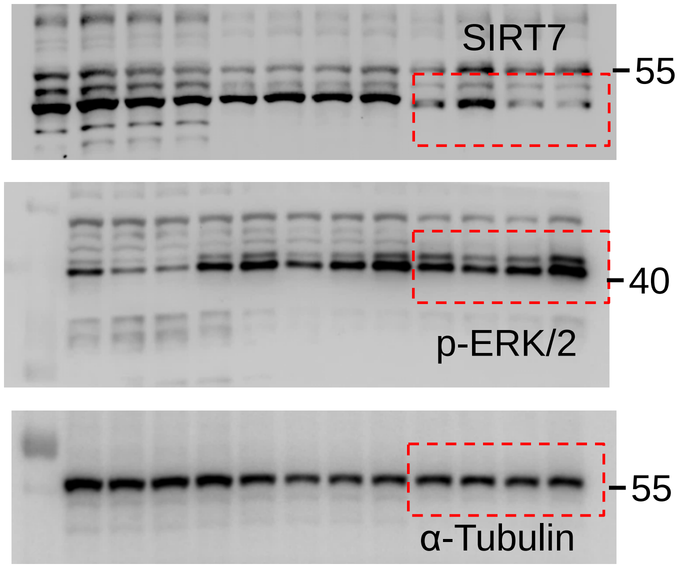

Supplementary Fig. 11j

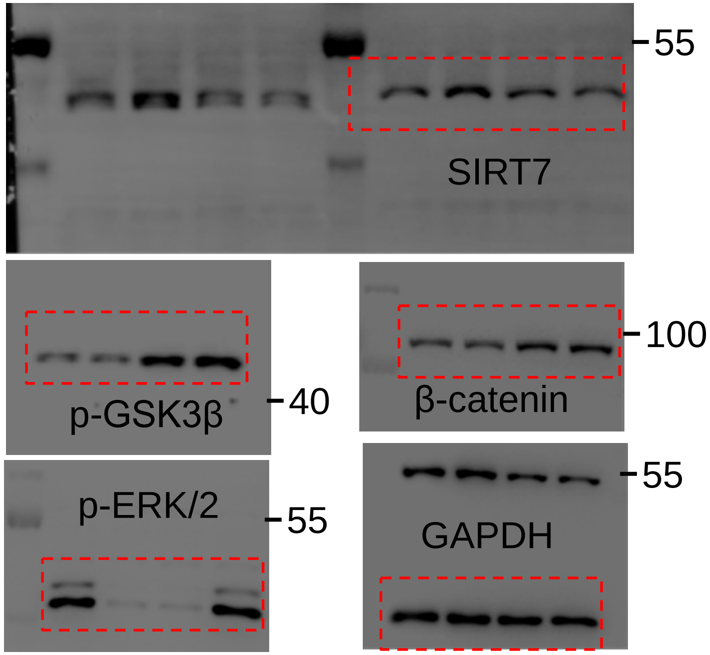

Supplementary Fig. 11g

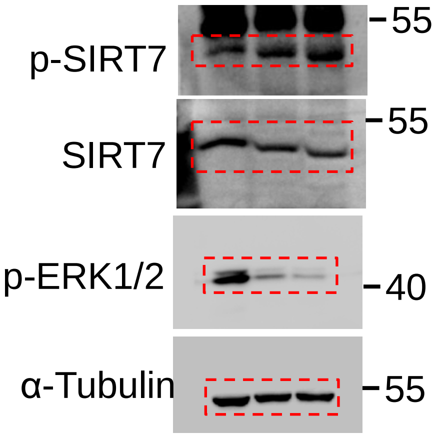

Supplementary Fig. 11h

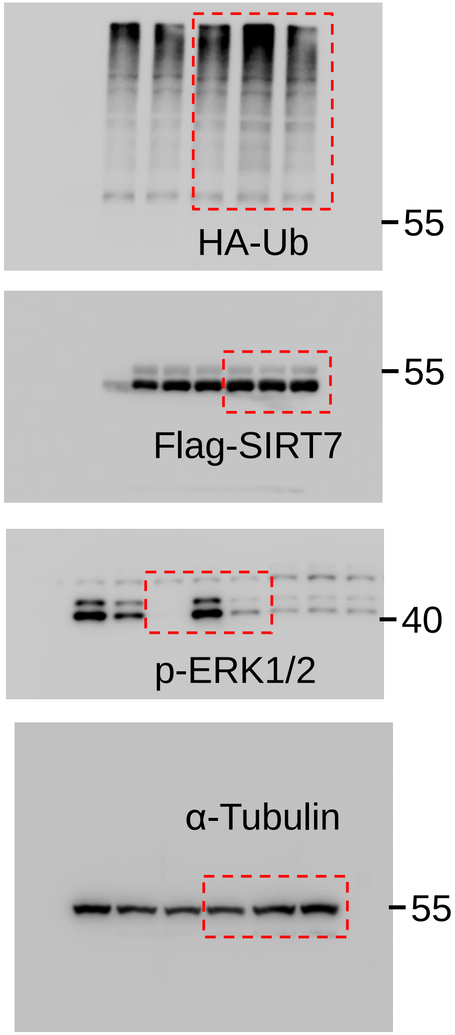

Supplementary Fig. 12

Supplementary Fig. 12a

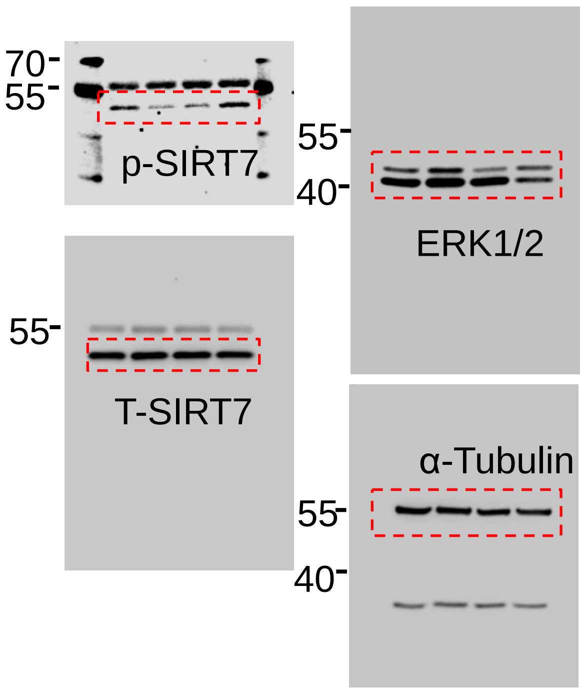

Supplementary Fig. 12c

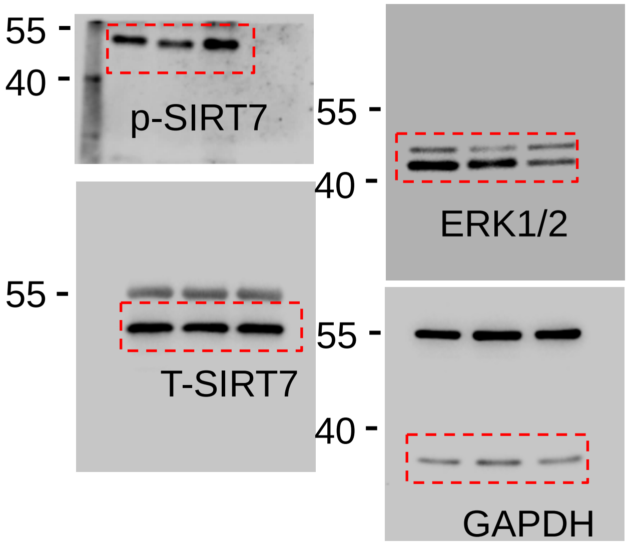

Supplementary Fig. 12e

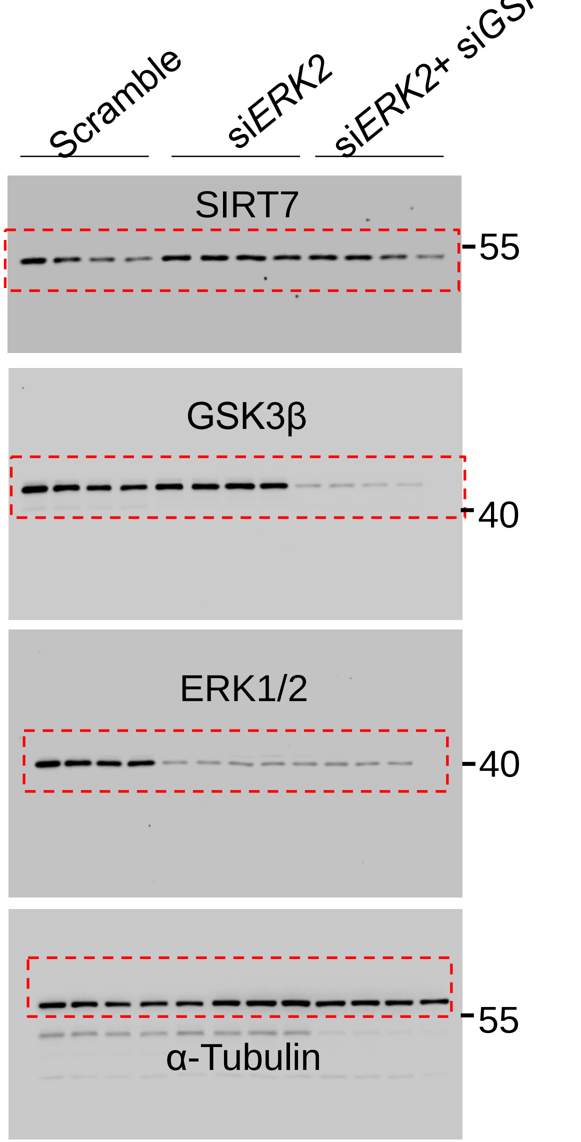

Supplementary Fig. 12

Supplementary Fig. 12g

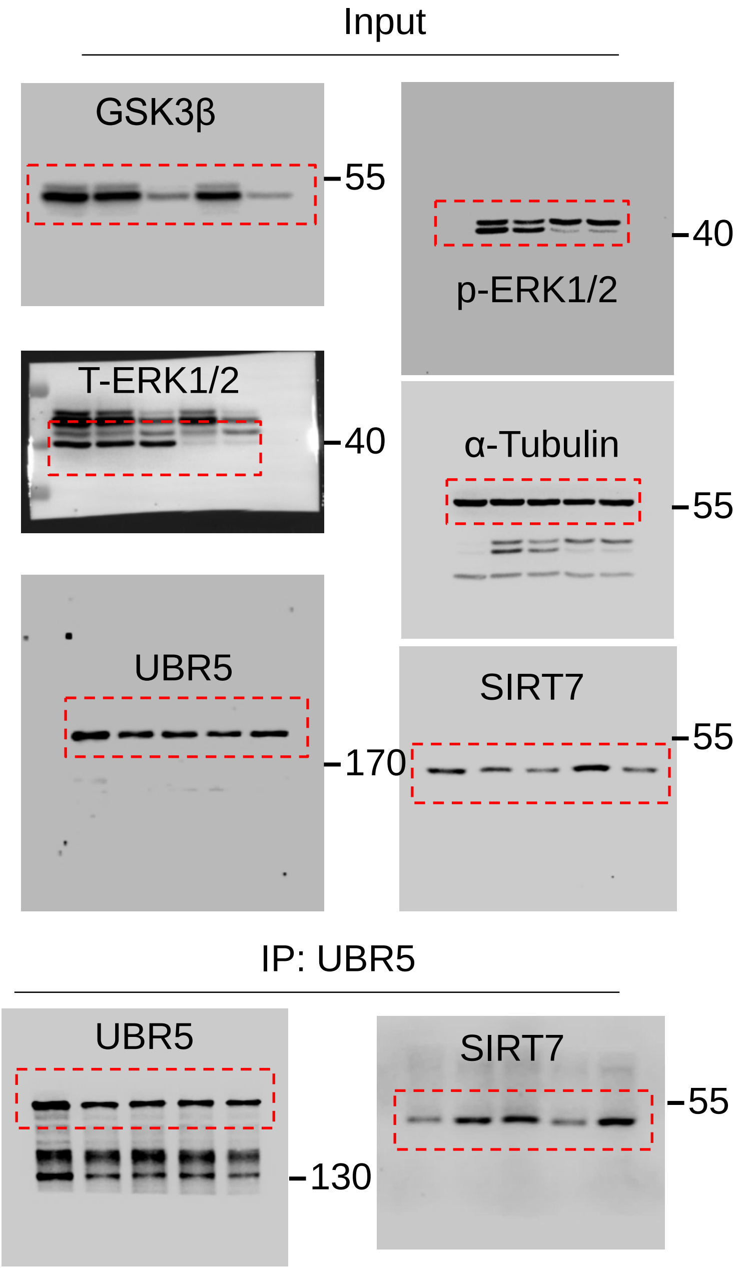

Supplementary Fig. 12h

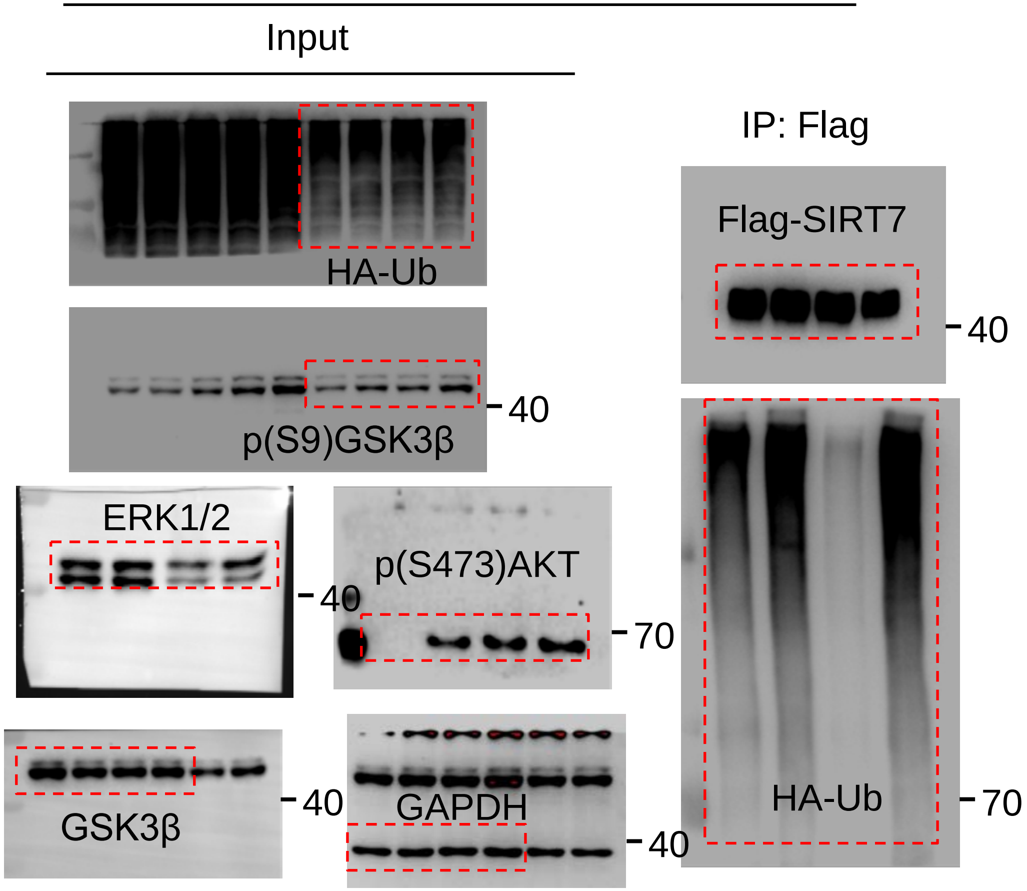

Supplementary Fig. 13

Supplementary Fig. 13a

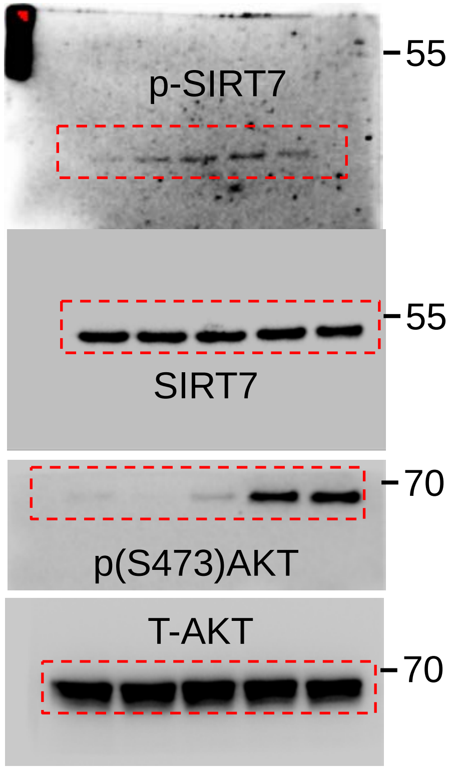

Supplementary Fig. 13d

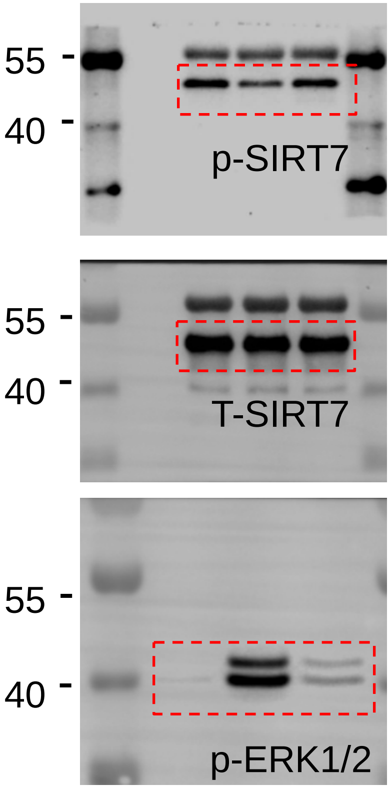

Supplementary Fig. 13e

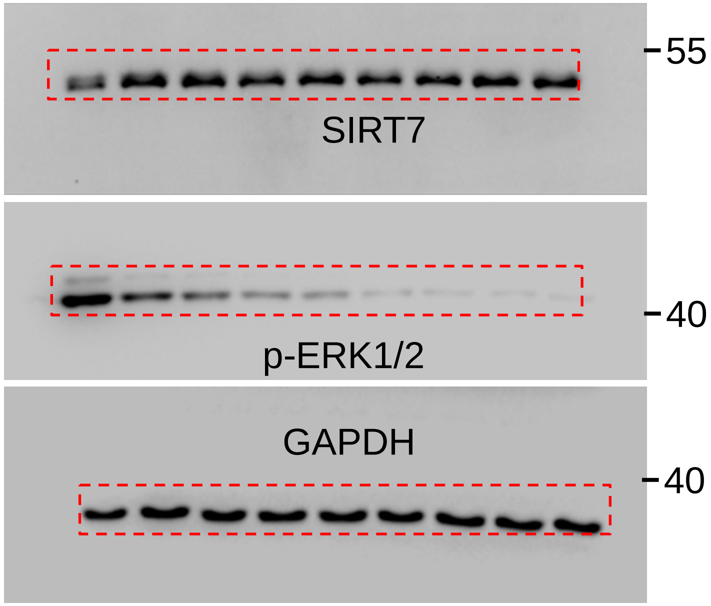

Supplementary Fig. 14

Supplementary Fig. 14a

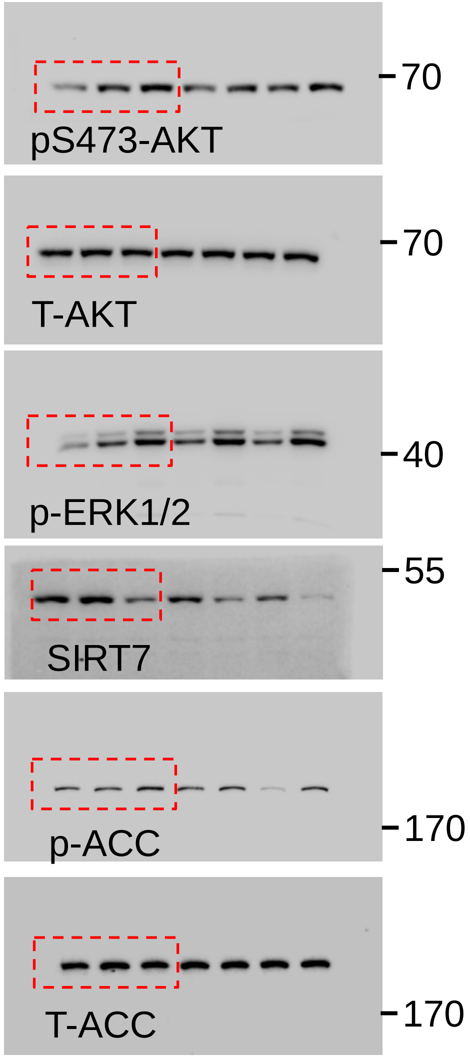

Supplementary Fig. 14b

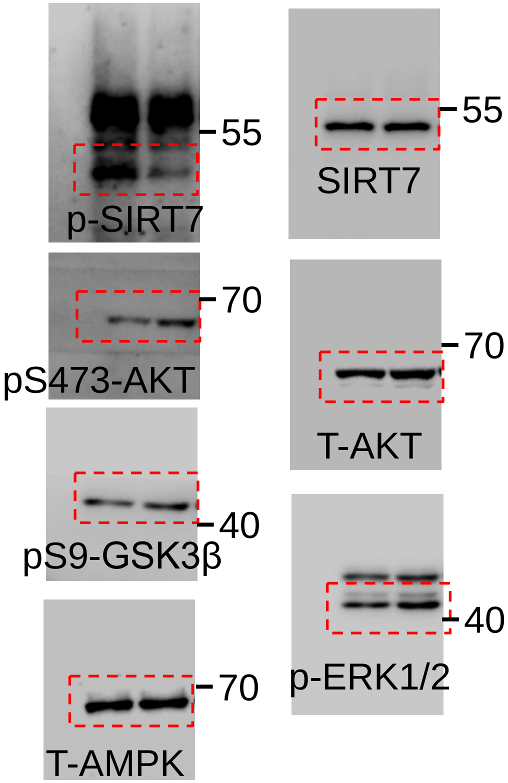

Supplement: Supplementary file 3 — Source Data [file 41467_2021_25274_MOESM3_ESM.zip › Uncropped western blots.pdf]
